# Responsible artificial intelligence in healthcare: a systematic review on the use of ethical principles in the development and deployment of artificial intelligence

**DOI:** 10.1136/bmjdhai-2025-000086

**Published:** 2025-11-13

**Authors:** Imane Ihaddouchen, Stefan Buijsman, Giorgia Pozzi, Davy van de Sande, Andreas Alois Reis, Reggie Townsend, Jeroen van den Hoven, Diederik Gommers, Michel E van Genderen

**Affiliations:** 1 Department of Adult Intensive Care, Erasmus MC University Medical Center, Rotterdam, The Netherlands; 2 Erasmus MC Datahub, Erasmus MC University Medical Center, Rotterdam, The Netherlands; 3 Faculty of Technology, Policy and Management, Delft University of Technology, Delft, The Netherlands; 4 Department of Research for Health, World Health Organization, Geneva, Switzerland; 5 SAS Institute, Cary, North Carolina, USA

**Keywords:** Artificial intelligence, Machine Learning

## Abstract

**Objective:**

As hospitals increasingly adopt artificial intelligence (AI) to manage rising patient volumes, workforce shortages and healthcare costs, concerns about ethical implementation have become prominent. This systematic review aims to assess how hospital-focused AI literature addresses the WHO’s six ethical AI principles—autonomy; well-being and safety; transparency and explainability; responsibility and accountability; inclusiveness and equity; and responsiveness and sustainability.

**Methods and analysis:**

A systematic review (PROSPERO registration: CRD42022347871) was conducted by searching Embase, MEDLINE ALL, Web of Science and the Cochrane Central Register of Controlled Trials from inception to December 2023, supplemented by Google Scholar. English-language studies describing AI (machine learning, deep learning, predictive analytics) relevant to inpatient settings and referencing at least one WHO principle were included. Two reviewers independently screened titles, abstracts and full texts, extracting data on publication year, country, study design, AI type, technology readiness level and ethical considerations. Discrepancies were resolved by consensus.

**Results:**

Of 4770 unique records, 673 were included. Most (83%) originated from high-income countries, with publication volume rising sharply after 2021. Of these, 558 (83%) addressed at least one WHO principle in depth, most frequently inclusiveness and equity (49%), transparency and explainability (45%) and autonomy (42%). Well-being and safety (26%) and responsibility and accountability (29%) were less frequently covered, while responsiveness and sustainability (6%) was rarely explored. Among 44 studies developing AI applications with technology readiness levels 1–6, ethical principles were acknowledged but rarely operationalised.

**Conclusion:**

Hospital-based AI research demonstrates increasing attention to ethical principles but lacks comprehensive application, particularly regarding sustainability. High-income countries dominate this discourse, underscoring the need for broader global engagement. To achieve equitable, safe and sustainable AI in clinical practice, clearer operational guidance and more inclusive collaboration is warranted.

**WHAT IS ALREADY KNOWN ON THIS TOPIC**

- Ethical concerns such as bias, transparency and accountability have been increasingly recognised in artificial intelligence (AI) applications in healthcare. However, no systematic review has previously assessed how the WHO’s ethical AI principles are integrated specifically within hospital-based AI research across different stages of AI readiness.

**WHAT THIS STUDY ADDS**

- This systematic review demonstrates that while ethical principles, particularly inclusiveness (49%) and transparency (45%), are frequently acknowledged in hospital-based AI research, they are rarely operationalised. Responsibility (29%), well-being (26%) and sustainability (only 6%) receive significantly less attention.

**HOW THIS STUDY MIGHT AFFECT RESEARCH, PRACTICE OR POLICY**

- These findings highlight a critical gap in integrating WHO’s ethical AI principles into clinical AI applications, emphasising the urgent need for standardised ethical frameworks, practical guidelines and focused attention on sustainability and accountability in future hospital-based AI research and implementation.

## Introduction

Healthcare systems worldwide face significant challenges, including rising patient volumes, workforce shortages and escalating costs. These challenges threaten the consistent delivery of high-quality care. Artificial intelligence (AI) has the potential to alleviate some of these burdens by improving diagnostic accuracy, streamlining administrative tasks and supporting clinical decision-making. However, the implementation of AI in clinical settings introduces serious ethical challenges and risks.

A growing body of literature shows that AI can exhibit significant biases in healthcare, often reflecting historical and structural inequities.^1–3^ For example, a landmark study by Obermeyer *et al*^4^ demonstrated how algorithmic bias disproportionately affected Black patients, leading to inequitable undertreatment. Similarly, an AI application introduced to detect various skin conditions was trained on a dataset with fewer than 4% of images from individuals with dark skin tones, risking misdiagnosis in underrepresented populations.^5^

In response to these concerns, the field has turned attention to responsible AI design and deployment, leading to the development of numerous AI ethics initiatives.^6–9^ Recently, the Trustworthy & Responsible AI Network Europe (TRAIN-Europe), a consortium of leading European experts in healthcare AI, has been established to help healthcare organisations improve their technical and data infrastructure, assess their AI maturity and determine their next steps.^10^ In addition, the Standards for Data Diversity, Inclusivity and Generalisability (STANDING Together) programme has presented 29 consensus recommendations to enable the identification and mitigation of algorithmic biases that might exacerbate health inequalities.^11^ Despite these efforts, a uniform framework for the responsible use of AI remains lacking.^12^

Recognising the importance of ethical guidance specifically tailored to healthcare, the WHO published a comprehensive guidance document outlining six key principles for AI in healthcare.^13^ While these principles highlight central concerns—such as fairness, transparency and accountability—they remain relatively abstract and can be challenging for stakeholders to operationalise. To advance from principles to practice, it is essential to assess the current state of ethical AI integration within hospital-based AI research and applications. Understanding how existing ethical AI principles are being adopted, where they fall short, and identifying areas for improvement are crucial steps towards developing actionable requirements.

This systematic review aims to evaluate the extent to which the WHO’s ethical principles for AI in healthcare are integrated into hospital-based AI research and deployment. By mapping the current landscape, this review seeks to identify gaps that need to be addressed to enhance the responsible operationalisation of AI in clinical settings.

## Materials and methods

This systematic review adheres to the Preferred Reporting Items for Systematic reviews and Meta-Analyses guideline^14^ and was registered prospectively in the online PROSPERO database (reference number: CRD42022347871).^15^

### Search strategy

A comprehensive search was conducted in four databases and registers (Embase, MEDLINE ALL, Web of Science and the Cochrane Central Register of Controlled Trials) from their inception to December 2023. We additionally used Google Scholar to identify any relevant literature that was not captured by the primary databases. The medical library of the Erasmus Medical Center assisted with the development of the search strategy. The search strategy included terms related to three core concepts: (1) AI (eg, “machine learning,” “deep learning,” “predictive analytics”), (2) inpatient hospital care (eg, “hospital,” “clinical,” “intensive care”) and (3) ethics (eg, “ethical,” “social responsible,” “morality”). The complete search strategies used for each database can be found in online supplemental etable 1.

10.1136/bmjdhai-2025-000086.supp1 Supplementary data

### Selection criteria

Studies were eligible for inclusion if they met the following criteria: they addressed AI algorithms (defined as computational models capable of learning from substantial datasets); they discussed one of the six WHO ethical principles for AI in healthcare (ie, protecting human autonomy, promoting well-being and safety, ensuring transparency and explainability, fostering responsibility and accountability, ensuring inclusiveness and equity and promoting responsiveness and sustainability; online supplemental etable 2)^13^; and they focused on inpatient or hospital-related use or included ethical considerations explicitly applicable to inpatient care. Only studies in which the ethical discussions aligned with at least one of the WHO ethical AI principles—or its substantive equivalent—were included. Both original and non-original research (eg, reviews, perspectives, policy papers) were considered to capture a comprehensive range of medical, technical, ethical and policy perspectives. Studies solely examining non-hospital contexts or AI tasks not directly relevant to patient health were excluded. For example, studies predicting clinical outcomes were eligible, whereas those predicting department crowding were excluded. Only full-text publications written in English were considered.

### Study selection 

After identifying relevant publications, duplicates were removed using EndNote V.20 (Clarivate Analytics, Philadelphia, Pennsylvania, USA). Two reviewers (II and GP) independently screened the titles and abstracts for potential eligibility; discrepancies were resolved by discussion or, if necessary, by consultation with a third reviewer (MEvG). Articles deemed eligible or unclear at the abstract stage proceeded to full-text review by two reviewers (II and DvdS). Discrepancies were resolved by consensus. Reasons for exclusion are provided in online supplemental etable 3.

### Data collection and analysis

A standardised data extraction form was used to gather information on the year of publication, country of publication, study design (eg, descriptive, experimental, observational), type of paper (eg, original research, review, viewpoint, comment), domain (eg, medical, technical, ethics or policy), medical specialty, AI type and AI readiness level (online supplemental etable 4). Any uncertainties were resolved through discussion among four reviewers (II, SB, GP and DvdS). First-author affiliations were classified according to the 2025 World Bank’s Gross National Income categories: low-income (<US$1145), lower-middle-income (US$1146–US$4515), upper-middle-income (US$4516–US$14 005) and high-income (>US$14 006).^16^

Clinical readiness of AI applications, and as such technological readiness, was assessed using the Technology Readiness Level (TRL) framework originally introduced by the National Aeronautics and Space Administration (NASA), adapted here for clinical settings.^17^ These levels range from problem identification (TRL 1) to full clinical integration (TRL 9). Only articles describing model development or testing in a clinical context were assigned TRL ratings. A detailed description of the clinical AI TRLs is provided in online supplemental etable 5.

Ethical considerations were mapped to the WHO’s six principles for AI in healthcare. A panel comprising a researcher, a physician and a philosopher (II, MEvG, SB) developed a list of keywords for each principle (see online supplemental etable 6). Two reviewers (II, DvdS) independently screened the full texts for these keywords, confirming that they were used in an ethical context rather than a purely procedural statement (eg, simple mention of ‘informed consent’ without discussion of ethical implications). We then assessed whether each principle was addressed briefly or in depth. The level of consideration (in-depth consideration vs brief mention) was determined using predefined criteria (online supplemental file etable 7). In-depth assessments involved extended ethical reasoning, illustrative examples or integration into study methods or results. In contrast, brief mentions comprised cursory references—such as listing a principle without elaboration. For instance, one study describing a bias mitigation strategy during model development was classified as in depth for WHO principle 5, while another that simply noted equity as ‘important to consider’ was rated as a brief mention. Prior to this process, both reviewers conducted a calibration round on a sample of 10 articles. Discrepancies in interpretation were discussed to align coding expectations and ensure consistent application of the ethical classification criteria. Any remaining disagreements during full coding were resolved through consensus or, if needed, consultation with MEvG or SB.

Article characteristics (design, domain and type) were summarised using descriptive statistics. Geographic distribution was visualised based on first-author country affiliations. Proportions of discussed ethical principles and AI readiness levels were calculated. For studies developing specific AI models, we plotted TRLs against the number and types of principles discussed to explore patterns between ethical considerations and AI maturity. No additional quantitative synthesis (eg, meta-analysis) was undertaken, given the largely qualitative and heterogeneous nature of the data.

### Patient and public involvement

Patients were not involved in any aspect of the study design, conduct, or in the development of the research question or outcome measures.

## Results

**Figure 1:**
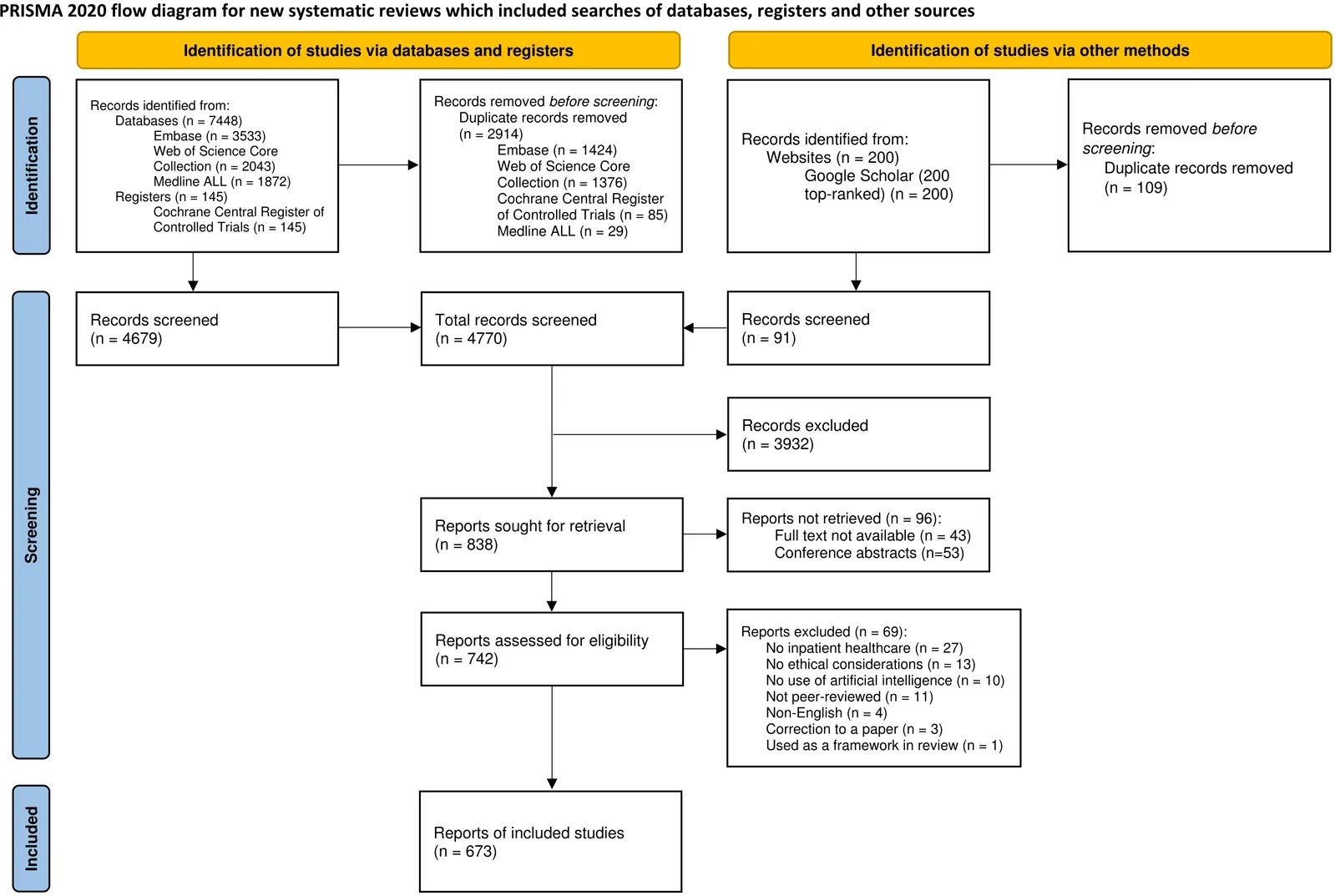
Preferred Reporting Items for Systematic reviews and Meta-Analyses flow diagram^14^

Our comprehensive search across databases and registers identified a total of 4679 publications, while an additional search in Google Scholar yielded 91 publications, duplicates excluded. After screening titles and abstracts, 3932 publications were excluded. The full text of the remaining 742 records was reviewed, leading to 673 publications that met all inclusion criteria (figure 1). Inter-rater agreement was generally high, with 94% agreement in title and abstract screening and 96% in full-text screening.

Of the 673 included studies, only one study was published prior to 2016 (in 2007). Annual publication counts have grown rapidly from a single publication in 2016 to 277 in 2023. The distribution of publications shows a majority in the medical domain (355/673, 53%) and the ethics domain (212/673, 32%), followed by smaller proportions in the technical domain (81/673, 12%) and the policy domain (25/673, 4%). The most common article types were reviews (298/673, 44%), followed by original research articles (170/673, 25%) and opinion or viewpoint pieces (124/673, 18%) (table 1; online supplemental appendix A).

10.1136/bmjdhai-2025-000086.supp2 Supplementary data

**Table 1:** Classification of included studies by domain, article type and study design

	Medical domain	Ethics domain	Technical domain	Policy domain	Subtotal (n (%))
Article type*					
Review	213 (32%)	44 (7%)	31 (5%)	10 (1%)	298 (44%)
Original research	50 (7%)	80 (12%)	33 (5%)	7 (1%)	170 (25%)
Opinion paper	46 (7%)	59 (9%)	14 (2%)	5 (1%)	124 (18%)
Other	46 (7%)	29 (4%)	3 (0%)	3 (0%)	81 (12%)
Subtotal (n (%))	355 (53%)	212 (32%)	81 (12%)	25 (4%)	673 (100%)
Study design					
Descriptive	312 (46%)	196 (29%)	53 (8%)	22 (3%)	583 (87%)
Experimental	4 (1%)	1 (0%)	18 (3%)	0 (0%)	23 (3%)
Observational	18 (3%)	3 (0%)	7 (1%)	0 (0%)	28 (4%)
Qualitative	17 (3%)	9 (1%)	2 (0%)	1 (0%)	29 (4%)
Mixed-methods	4 (1%)	3 (0%)	1 (0%)	2 (0%)	10 (1%)
Subtotal (n (%))	355 (53%)	212 (32%)	81 (12%)	25 (4%)	673 (100%)

Data are presented as numbers and proportions (%). Studies are classified by their primary focus domain: the **medical domain** includes studies focusing on the end-user, such as patients or healthcare professionals, either as the target audience or central topic. The **technical domain** includes studies where the target audience consists of engineers or AI developers, or where the main focus is model development and/or technical analyses. The **ethics domain** includes studies primarily targeting ethicists or philosophers, or those where the main objective is an ethical analysis of AI in healthcare. The **policy domain** includes studies addressing policymakers or legal experts as the target audience, or those focussing on legal and regulatory aspects of AI, including AI governance and policymaking.

*Article types are grouped as follows: review (scoping, systematic, narrative reviews and review protocols), original research (original research articles, case reports, consensus statements, frameworks and research protocols), opinion paper (expert discussions, perspectives and viewpoints) and other (commentaries, correspondence, editorials, essays, letters to the editor and white papers).

AI, artificial intelligence.

Regarding geographic distribution based on first-author affiliations, 558 (83%) of the 673 studies originated from high-income countries, 80 (12%) from upper-middle-income countries, 35 (5%) from lower-middle-income countries and none from low-income countries. The USA and the UK were the most represented, accounting for 200 (30%) and 70 (10%) publications, respectively. Latin America (five papers, 1%) and Africa (four papers, 1%) were minimally represented in first-author affiliations (online supplemental efigure 1).

### AI ethical principles

Of the 673 included studies, 441 (66%) were published after the release of the WHO guidance document in 2021. In total, 558 (83%) of the studies considered one or more WHO ethical principles in depth, while the remaining 115 (17%) were included only on the basis of brief mentions. Of those 558 in-depth studies, 190 (28%) addressed a single WHO ethical principle, and 368 (55%) addressed multiple principles in depth.

**Figure 2:**
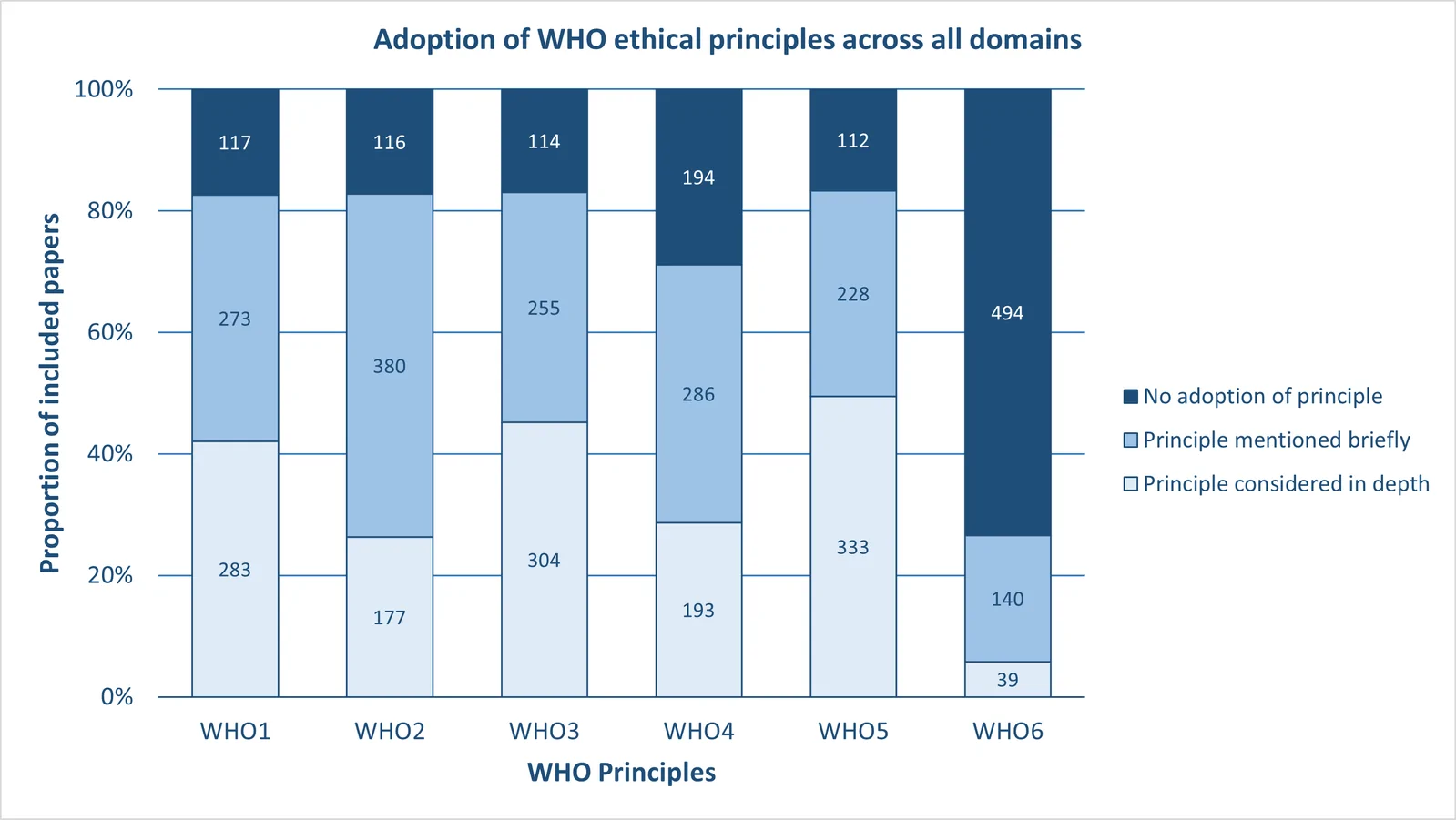
Adoption of the six WHO ethical principles across all included papers (N=673). Each bar shows the proportion of included papers that consider the principle in depth (light blue), mention it briefly (medium blue) or do not adopt the principle (dark blue). WHO 1=protecting human autonomy; WHO 2=promoting well-being and safety; WHO 3=ensuring transparency and explainability; WHO 4=fostering responsibility and accountability; WHO 5=ensuring inclusiveness and equity; WHO 6=promoting responsive and sustainable artificial intelligence.

Among these 558 in-depth studies, some principles were considerably more prominent than others. WHO principle 5 (ensuring inclusiveness and equity) topped the list with 333 (49%) studies offering in-depth discussions. WHO principle 3 (ensuring transparency, explainability and intelligibility) appeared next in 304 (45%) studies, closely followed by WHO principle 1 (protecting autonomy) in 283 (42%) studies. WHO principle 4 (fostering responsibility and accountability) and WHO principle 2 (promoting human well-being, safety and the public interest) were each thoroughly considered less frequently—193 (29%) and 177 (26%) studies, respectively. Most notably, WHO principle 6 (promoting AI that is responsive and sustainable) was the least represented, with only 39 (6%) studies considering it in depth (figure 2). Keyword presence and level of consideration for each principle are summarised in online supplemental etable 8. Notably, the distribution of principles addressed showed minimal differences between studies published before and after the release of the 2021 WHO guidance. Specifically, the average number of WHO principles discussed in depth per study remained consistent at 2.0 before and after the guidance’s publication.

### Adoption of ethical principles in generative AI

Sixty of the 673 included studies focused specifically on generative AI, and their ethical discussions closely resembled those in the broader dataset. In line with the larger sample, WHO principles 1 (autonomy) and 5 (inclusiveness and equity) were most frequently discussed in depth, appearing in 24 (40%) and 25 (42%) of these studies, respectively. WHO principles 2, 3 and 4 were more often mentioned briefly than considered in depth. WHO principle 6 (responsiveness and sustainability) remained least addressed, with only one study (2%) discussing it in depth (online supplemental efigure 2).

### Adoption of ethical principles in relation to AI maturity

Out of the 673 included studies, 44 focused on developing specific clinical AI applications and were assigned a level of readiness based on the TRL scale. Among these 44 studies, 31 (70%) were classified at TRL 4 or below, 12 (27%) focused on model validation (TRL 5) and one (2%) study reported real-time model testing without implementation into the clinical workflow (TRL 6). No studies were identified discussing ethical principles at TRL 7 or higher. Detailed characteristics of these studies are provided in table 2.

**Table 2:** Characteristics of studies developing clinical AI applications (n=44)

Study characteristics	N (%)
Year of publication	
2016	1 (2%)
2019	3 (7%)
2020	3 (7%)
2021	6 (14%)
2022	14 (32%)
2023	16 (36%)
2024	1 (2%)
Geographical region	
Northern America	17 (39%)
Western Europe	11 (25%)
Asia	11 (25%)
Australia	2 (5%)
Eastern Europe	2 (5%)
Southern Europe	1 (2%)
TRL	
1	12 (27%)
2	2 (5%)
3 and 4	17 (39%)
5	12 (27%)
6	1 (2%)
7	0 (0%)
8	0 (0%)
9	0 (0%)
Domain	
Technical	22 (50%)
Medical	20 (45%)
Ethics	2 (5%)
Policy	0 (0%)
Medical specialty	
Internal medicine	14 (32%)
Diagnostics and imaging	7 (16%)
Critical and emergency care	4 (9%)
Paediatric and maternal care	4 (9%)
Specialised care	4 (9%)
Surgical specialities	3 (7%)
Psychiatry	1 (2%)
General healthcare or multispecialty	7 (16%)
Type of AI	
General AI	11 (25%)
Machine learning	17 (38%)
Deep learning	11 (25%)
Generative AI	3 (7%)
Reinforcement learning	1 (2%)
Federated learning	1 (1%)

AI, artificial intelligence; TRL, technology readiness level.

**Figure 3:**
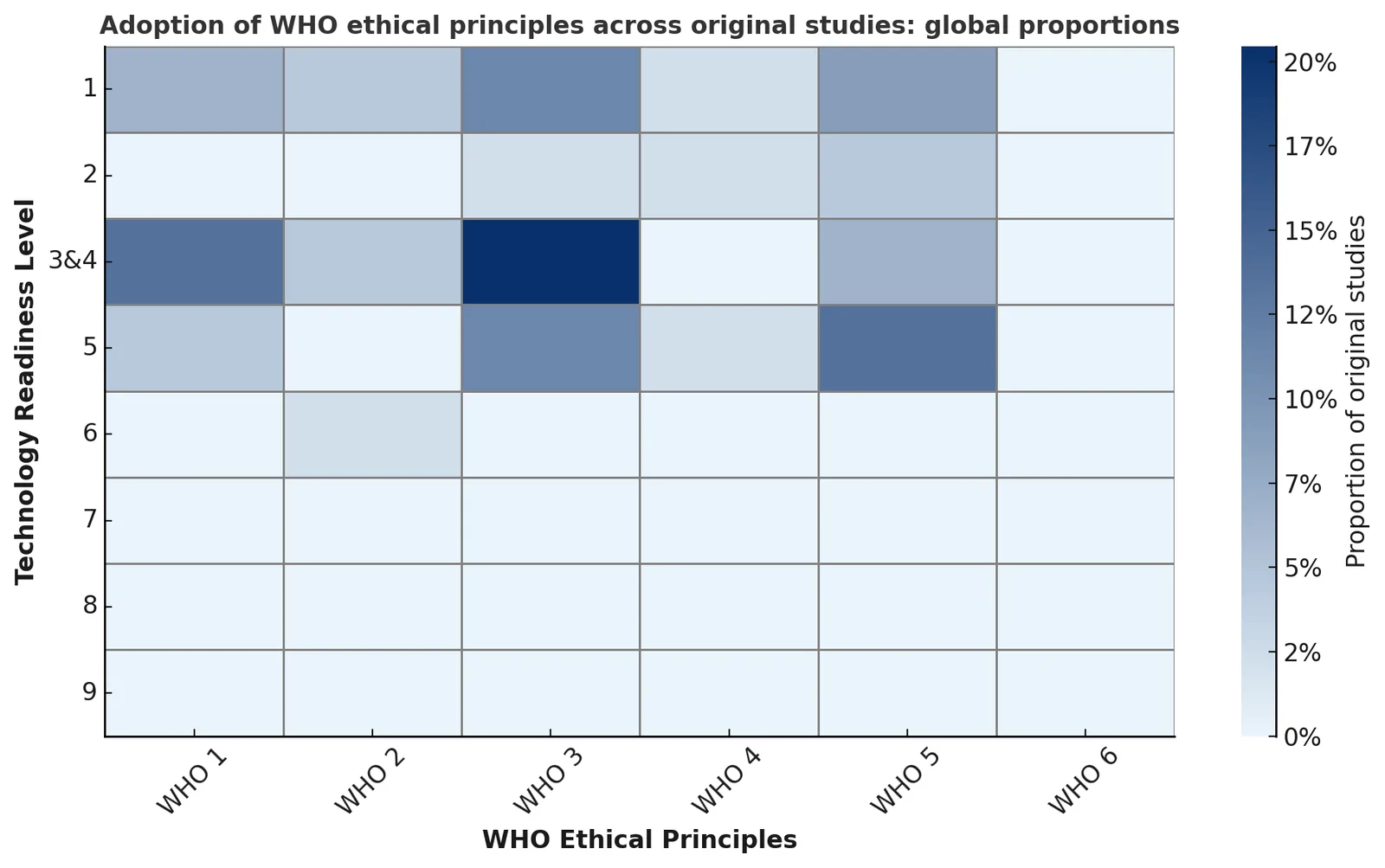
Adoption of the six WHO ethical principles by technology readiness level among clinical AI development studies (n=44). Cells show the percentage of original studies (of the 44) addressing each WHO ethical principle at the specified technology readiness level, with darker shades indicating higher proportions. WHO 1=protecting human autonomy; WHO 2=promoting well-being and safety; WHO 3=ensuring transparency and explainability; WHO 4=fostering responsibility and accountability; WHO 5=ensuring inclusiveness and equity; WHO 6=promoting responsive and sustainable AI. AI, artificial intelligence

In terms of ethical principles, 35 (80%) of the 44 studies discussed at least one ethical principle in depth, while the remaining 9 (20%) studies mentioned them only briefly. Specifically, 12 (27%) studies addressed WHO principle 1 in depth, 5 (11%) studies considered WHO principle 2, 21 (48%) studies considered WHO principle 3, 3 (7%) considered WHO principle 4 and 16 (36%) studies considered WHO principle 5. Notably, none of these studies addressed WHO principle 6 in depth (figure 3). The proportions of studies that only briefly mentioned these principles are presented in online supplemental efigure 3.

## Discussion

The results of this review underscore an uneven and often superficial integration of ethical AI principles within hospital-based AI research. Although the majority of studies reference at least one of the six WHO ethical principles, the depth of consideration varies considerably. Inclusiveness and equity, along with transparency and explainability, receive the most attention, whereas responsibility, well-being and especially sustainability remain comparatively underexplored. This gap is further reflected in studies that develop specific AI models, where the importance of ethical considerations is frequently acknowledged but rarely put into practice. For example, Che *et al*^18^ reported in-depth discussion of WHO principle 3 (interpretability) by designing a deep learning model for ICU mortality prediction that includes visualisation of time-varying feature importance. Tranter-Entwistle *et al*^19^ mention the relevance of explainability (WHO principle 3) in the discussion of deployment challenges, without elaborating on technical or procedural implementation. We considered this to be an example of a brief mention of the principle. This pattern is compounded by a striking geographic imbalance: high-income countries account for 83% of all included publications, effectively shaping the global narrative on responsible AI in healthcare. Collectively, these findings illustrate the persistent challenge of translating ethical guidelines into concrete actions and highlight the urgent need for more comprehensive frameworks that bridge the principle–practice divide.

Only very few (6%) studies took AI’s sustainability into account, highlighting a critical under-representation of WHO principle 6. This limited focus may stem from the principle’s emphasis on broader business and environmental considerations, which are often perceived as less directly relevant to clinicians and patient care. However, this narrow focus fails to address an important ethical dilemma: the trade-off between the benefits of training AI on large datasets and the substantial environmental costs associated with these processes. On the one hand, AI can be leveraged to reduce greenhouse gas emissions in healthcare by about 80%^20^ and help optimise resource efficiency, aligning with the green transition’s broader aims of more responsible use of resources.^21^ On the other hand, training a single AI model can generate over 284 000 kg of carbon dioxide equivalent emissions, comparable to nearly five times the lifetime emissions of an average American car.^22 23^ The under-representation of sustainability in the literature—and its absence among AI applications at higher TRLs—is concerning. Several factors may explain this persistent gap. One key issue is that sustainability is rarely integrated into clinical AI reporting standards, which limits both developer awareness and accountability. For instance, the recently proposed Transparent Reporting of a multivariable model for Individual Prognosis Or Diagnosis – Large Language Models (TRIPOD-LLM) checklist—one of the first to address this issue—recommends reporting computational demands (eg, machine usage, inference time or floating-point operations),^24^ yet concrete metrics on energy use or environmental impact remain absent. Furthermore, sustainability is often perceived as less urgent than concerns such as diagnostic accuracy, patient safety or clinical integration. Lastly, current regulatory and funding structures rarely mandate environmental impact assessments, further limiting developer incentives to incorporate sustainability during model development. Responsible AI requires a more holistic perspective, recognising that AI has significant resource demands and climate impacts, posing significant challenges to achieving the United Nations’ Sustainable Development Goals.^25 26^ It is therefore particularly alarming that, within the subset of 60 studies focusing on generative AI, the same lack of attention to WHO principle 6 is evident, with only one (2%) study addressing sustainability concerns in depth. Although recent initiatives such as the European Union’s Artificial Intelligence Act,^27^ the European Green Deal^28^ and the US National Artificial Intelligence Advisory Committee’s (NAIAC) Year-Two Insights Report^29^ acknowledge the importance of sustainability in trustworthy AI, they still fall short of proposing concrete actions. Therefore, policymakers and healthcare leaders should consider standardising metrics for measuring energy consumption and environmental footprints, particularly as demand grows for computationally intensive models. Sustainability affects not just academic debates but also patient safety, healthcare costs and global equity.

Although most studies considered at least one WHO ethical principle in depth, the overall distribution is uneven. Most efforts have focused on inclusiveness and equity (WHO principle 5) and transparency, explainability and intelligibility (WHO principle 3). The focus on equity aligns with global efforts to protect vulnerable patient groups from discriminatory outcomes and resonates with broader developments in AI ethics.^9 30^ Indeed, AI holds promise for enhancing healthcare access and efficiency, yet it risks perpetuating structural or cultural racism when the underlying data reflect existing societal inequities.^1 4 5 31–33^ These concerns are amplified in the case of generative AI. Like other models, generative AI systems inherit biases from their training data—yet unlike traditional models, their training datasets are often vast, opaque and poorly documented. This limited traceability makes it difficult to assess representativeness or audit for structural bias, increasing the risk of perpetuating health inequities. In addition to this, generative models may produce fabricated yet clinically plausible content, so-called ‘hallucinations’, that can mislead clinicians or propagate misinformation. It is therefore striking that WHO principle 2 related to safety and the public interest was among the least addressed in generative AI studies. These findings suggest that much of the current discourse, including on generative AI, either lacks specific, actionable steps or simply reiterates theoretical considerations. For instance, while numerous papers acknowledge biases in training data, few propose practical methods—such as standardised fairness audits^34^ or continuous bias monitoring^35^—to mitigate these risks. Recent calls to strengthen algorithmic transparency in updates to the Declaration of Helsinki and in emerging AI guidelines further underscore the need for accountability and openness.^36 37^ Ultimately, these efforts aim to enhance trust among patients and the public, which is crucial for achieving meaningful, long-term integration of AI in clinical practice.

Our analysis indicates that explicit attention to ethical considerations in clinical AI was practically absent before 2018, with only one publication identified in 2007 and another in 2016. From 2018 onward, there was a steady rise in publications, which accelerated notably after the release of the WHO guidance document in 2021. While the WHO guidance may have helped raise awareness of AI ethics in healthcare, another contributing factor to this surge is the broader expansion of medical AI research itself.^38^ As technological advances made the development and deployment of clinical AI applications increasingly feasible, more studies were conducted, naturally drawing greater attention to responsible AI practices and the ethical principles governing these technologies. Despite the growth in publications, the average number of WHO principles addressed per study has remained stable before and after the guidance’s release. This observation suggests that the heightened awareness of AI ethics in healthcare has not necessarily translated into a broader or deeper engagement with multiple ethical principles across studies. Indeed, most studies only briefly mention these principles rather than examining them in depth. Although researchers appear to recognise the importance of ethical frameworks, many do not thoroughly explore or operationalise these principles—particularly in studies developing or testing new AI applications. This pattern is evident in our analysis of clinical AI studies with higher TRLs. Although TRL scores reflect technological maturity, they do not correlate with deeper ethical integration. Even at TRL 5 and 6, near-deployment stages, most studies focused narrowly on a single principle, and ethical aspects were often only briefly addressed. For instance, none of these studies operationalised sustainability (WHO principle 6), and only a few considered autonomy (WHO principle 1) or responsibility (WHO principle 4) (see figure 3). This highlights the disconnect between technical progress and ethical engagement, underscoring the need for explicit checkpoints linking AI readiness to responsible design. This persistent superficiality, observed across both early-stage (low TRL) and more mature AI studies (higher TRL), may stem from the abstract nature of existing ethical guidelines. While the WHO guidance and similar frameworks from the European Commission,^39^ the United Nations Educational, Scientific and Cultural Organization (UNESCO)^40^ and the American Medical Informatics Association (AMIA)^41^ emphasise the importance of ethics, they often remain too abstract for AI developers seeking to implement them. These guidelines are often crafted for broad stakeholder audiences rather than specifically tailored to the needs of those who build and deploy AI tools. This limitation is increasingly recognised. Recent work^42^ similarly calls for an actionable ethical framework tailored to the AI development process. Together, these findings stress the importance of embedding practical ethical guidance throughout the AI lifecycle—not only to promote trust, but also to ensure responsible deployment at every readiness stage. To bridge the gap between abstract principles and practical implementation, more targeted tools are needed to support developers and implementers. Resources such as model cards for model reporting,^43^ datasheets for datasets^44^ and algorithmic impact assessments^45^ offer practical ways to embed ethical principles into system design and evaluation. Clinical research can benefit from reporting guidelines like Consolidated Standards of Reporting Trials – Artificial Intelligence (CONSORT-AI)^36^ and Standard Protocol Items: Recommendations for Interventional Trials – Artificial Intelligence (SPIRIT-AI)^37^ to promote transparency and standardisation. In addition, early-stage registration of AI algorithms can help document development processes, mitigate risks and enhance transparency prior to deployment.^46^

Consistent with prior research, the vast majority (83%) of studies originated from high-income countries.^47^ This geographic concentration raises important concerns—not only about the generalisability of findings, but also about whose values and assumptions shape AI ethics. When models and ethical frameworks originate mostly from high-income countries, they risk overlooking the needs and constraints of low-income and middle-income countries (LMICs). Data environments, clinical workflows and governance structures in LMICs often differ significantly. As a result, current standards may be ill-suited for global application. A recent demographic analysis nicely shows that over 90% of AI guideline developers are based in high-income countries—underscoring the geographic imbalance in who defines ethical standards for clinical AI.^48^ A more equitable research landscape requires globally inclusive approaches—not only in AI model development but also in the ethical frameworks that guide their use. This calls for capacity-building mechanisms, such as LMIC-led research hubs, South-South institutional partnerships and funding schemes that prioritise LMIC-led AI innovation. Such efforts are vital to ensure that AI development and governance are contextually relevant, equitable and globally legitimate. Equity challenges also extend to the demographic composition of the AI research workforce. A 2024 report from Stanford University shows that the percentage of female PhD graduates in AI and computer science in North America remains low, increasing only marginally from approximately 18% in 2010 to 22% in 2022.^49^ Hilling *et al*^48^ further show that among AI guideline developers, 73% are male and 61% White, highlighting structural homogeneity even in global standard-setting. This under-representation of perspectives may influence which values are embedded in AI systems. Addressing these disparities requires targeted efforts in mentorship, inclusive hiring and equitable participation in research leadership.

This review has several limitations that should be considered when interpreting its findings. First, although we conducted a systematic review of the literature up to 2024, the rapid influx of AI-related publications in the medical field remains a challenge. Traditional systematic reviews aim to critically synthesise all available evidence into a comprehensive overview. However, rapid healthcare AI developments increase the risk of findings becoming outdated by the time of publication. Even so, several journal guidelines suggest considering updates at 2-year intervals, indicating that this review remains timely and relevant.^50^ Notably, it is also the first of its kind in this research area. Moving forward, a living systematic review model could offer a more dynamic solution, allowing for continuous incorporation of the latest evidence. Second, we relied on first-author affiliation as a proxy for geographic origin, potentially under-representing multi-institutional collaborations. However, first-author affiliations generally indicate where a study is designed and data are processed, making it a reasonable proxy for geographic representation.^51^ Third, although we used a predefined list of keywords and established criteria to classify each article’s level of consideration of the WHO’s ethical principles, some subjectivity remains in determining the depth of discussion. To mitigate this, two reviewers independently assessed each article, resolving discrepancies with a third reviewer when necessary to ensure consistency and reliability. Lastly, we chose the WHO’s six principles as a framework due to the organisation’s global public health leadership and the extensive multistakeholder engagement in developing these principles. We acknowledge that other regional and domain-specific frameworks—such as those from the European Commission,^39^ UNESCO^40^ and AMIA^41^—may offer complementary insights not fully captured in this review.

### Conclusions

This systematic review highlights the limited practical application of ethical AI principles, particularly in relation to sustainability and the transition to operational use. To ensure responsible AI advancements, a more pragmatic and inclusive approach is needed. Ethical requirements must be systematically integrated across all phases of the clinical AI lifecycle. While core principles such as transparency, accountability and equity apply throughout, their operationalisation may differ by context and phase—for example, from data governance in early design to oversight and redress in postdeployment. Embedding ethics ‘by design’ is therefore essential: it enables developers and deployers to anticipate ethical challenges and embed safeguards from the outset.^52^ This approach helps translate abstract principles into concrete design choices, validation strategies and governance structures, and is crucial to advancing equitable, safe and sustainable clinical AI.

## Data Availability

All data relevant to the study are included in the article or uploaded as supplementary information. The appendix includes the full search terms for all databases, a list of studies excluded at the full-text screening stage (with brief reasons) and other relevant information. Additional data are available from the corresponding author upon request.
