# Supplementary material for: Responsible artificial intelligence in healthcare: a systematic review on the use of ethical principles in the development and deployment of artificial intelligence
Source: BMJ Digit Health Ai. 2025 Nov 13;1(1):e000086. doi: 10.1136/bmjdhai-2025-000086 (PMC13492534; doi:10.1136/bmjdhai-2025-000086)
Supplement: Supplementary file 1 [file bmjdhai-1-1-bmjdhai-2025-000086-s001.docx]

**Supplementary content**

Responsible artificial intelligence in healthcare: a systematic review on the use of ethical principles in the development and deployment of artificial intelligence *Imane Ihaddouchen, Stefan Buijsman, Giorgia Pozzi, Davy van de Sande, Andreas Alois Reis, Reggie Townsend, Jeroen van den Hoven, Diederik Gommers, Michel E. van Genderen.*

Contents

[eTable 1. Search strategy	2](#_Toc196235730)

[eTable 2. WHO ethical principles	4](#_Toc196235731)

[eTable 3. Excluded publications with reasons for exclusion	5](#_Toc196235732)

[eTable 4. Data extraction form	10](#_Toc196235733)

[eTable 5. Technology Readiness Level (TRL) scale	12](#_Toc196235734)

[eTable 6. Standardised list of relevant keywords per WHO ethical principle	13](#_Toc196235735)

[eTable 7. Criteria for evaluating degree of consideration	14](#_Toc196235736)

[eTable 8. Keyword presence and the level of consideration given to each principle by the included publications	15](#_Toc196235737)

[eFigure 1. Geographic distribution of included studies	84](#_Toc196235738)

[eFigure 2. Adoption of the six WHO ethical principles across generative AI publications (N=60)	85](#_Toc196235739)

[eFigure 3. Adoption of the six WHO ethical principles across studies developing clinical AI applications (N=44)	86](#_Toc196235740)

[References	87](#_Toc196235741)

This supplementary material has been provided by the authors to give readers additional information about their work.

# eTable 1. Search strategy

| *Database* | *Search string* |
| --- | --- |
| *Embase* | ('ethics'/exp OR 'morality'/exp OR 'social responsibility'/de OR (ethic* OR moral* OR immoral* OR unethic* OR bioethic* OR ((social) NEAR/3 (responsib*))):ab,ti,kw,jt) AND ('artificial intelligence'/exp OR 'machine learning'/exp OR 'natural language processing'/de OR (AI OR automated-reason* OR computer-heurist* OR ((natural) NEAR/3 (language) NEAR/3 (proces*)) OR ((bayes*) NEAR/3 (combiner* OR network*)) OR ((nearest) NEAR/3 (neighbo*)) OR ((random*) NEAR/3 (forest*)) OR ((support*) NEAR/6 (vector*) NEAR/6 (machine* OR active)) OR ((extract*) NEAR/6 (feature*) NEAR/6 (vector*)) OR ((artificial* OR ambient* OR machine* OR algorithm* OR neural) NEAR/3 (intelligen* OR network*)) OR ((automat*) NEAR/3 (pattern*) NEAR/3 (recogni*)) OR ((learn*) NEAR/3 (algori*)) OR ((machine* OR deep OR supervis* OR unsupervis* OR bayes) NEAR/3 (learn* OR vector*))):ab,ti,kw) AND ('hospital'/exp OR 'hospital care'/exp OR 'hospitalization'/de OR 'hospital patient'/exp OR 'clinical decision making'/exp OR 'practice guideline'/de OR (hospital* OR clinic* OR ((intensiv* OR critical) NEAR/3 (care*)) OR IC OR ICU OR intramural* OR inpatient* OR radiolog* OR cardiolog* OR guideline*):ab,ti,kw) AND [ENGLISH]/lim NOT ((animal/exp OR animal*:de OR nonhuman/de) NOT ('human'/exp)) |
| *Medline ALL* | (exp Ethics/ OR ethics.fs. OR Morals/ OR Social Responsibility/ OR (ethic* OR moral* OR immoral* OR unethic* OR bioethic* OR ((social) ADJ3 (responsib*))).ab,ti,kf,jw.) AND (exp Artificial Intelligence/ OR (AI OR automated-reason* OR computer-heurist* OR ((natural) ADJ3 (language) ADJ3 (proces*)) OR ((bayes*) ADJ3 (combiner* OR network*)) OR ((nearest) ADJ3 (neighbo*)) OR ((random*) ADJ3 (forest*)) OR ((support*) ADJ6 (vector*) ADJ6 (machine* OR active)) OR ((extract*) ADJ6 (feature*) ADJ6 (vector*)) OR ((artificial* OR ambient* OR machine* OR algorithm* OR neural) ADJ3 (intelligen* OR network*)) OR ((automat*) ADJ3 (pattern*) ADJ3 (recogni*)) OR ((learn*) ADJ3 (algori*)) OR ((machine* OR deep OR supervis* OR unsupervis* OR bayes) ADJ3 (learn* OR vector*))).ab,ti,kf.) AND (exp Hospitals / OR exp Hospital Departments/ OR exp Hospitalization/ OR exp Critical Care/ OR Inpatients/ OR exp Clinical Decision-Making/ OR exp Guideline/ OR (hospital* OR clinic* OR ((intensiv* OR critical) ADJ3 (care*)) OR IC OR ICU OR intramural* OR inpatient* OR radiolog* OR cardiolog* OR guideline*).ab,ti,kf.) AND english.la. NOT (exp Animals/ NOT Humans/) |
| *Web of Science Core Collection* | TS=(((ethic* OR moral* OR immoral* OR unethic* OR bioethic* OR ((social) NEAR/2 (responsib*)))) AND ((AI OR automated-reason* OR computer-heurist* OR ((natural) NEAR/2 (language) NEAR/2 (proces*)) OR ((bayes*) NEAR/2 (combiner* OR network*)) OR ((nearest) NEAR/2 (neighbo*)) OR ((random*) NEAR/2 (forest*)) OR ((support*) NEAR/5 (vector*) NEAR/5 (machine* OR active)) OR ((extract*) NEAR/5 (feature*) NEAR/5 (vector*)) OR ((artificial* OR ambient* OR machine* OR algorithm* OR neural) NEAR/2 (intelligen* OR network*)) OR ((automat*) NEAR/2 (pattern*) NEAR/2 (recogni*)) OR ((learn*) NEAR/2 (algori*)) OR ((machine* OR deep OR supervis* OR unsupervis* OR bayes) NEAR/2 (learn* OR vector*)))) AND ((hospital* OR clinic* OR ((intensiv* OR critical) NEAR/2 (care*)) OR IC OR ICU OR intramural* OR inpatient* OR radiolog* OR cardiolog* OR guideline*))) |
| *Cochrane Central Register of Controlled Trials* | ((ethic* OR moral* OR immoral* OR unethic* OR bioethic* OR ((social) NEAR/3 (responsib*))):ab,ti,so) AND ((AI OR automated-reason* OR computer-heurist* OR ((natural) NEAR/3 (language) NEAR/3 (proces*)) OR ((bayes*) NEAR/3 (combiner* OR network*)) OR ((nearest) NEAR/3 (neighbo*)) OR ((random*) NEAR/3 (forest*)) OR ((support*) NEAR/6 (vector*) NEAR/6 (machine* OR active)) OR ((extract*) NEAR/6 (feature*) NEAR/6 (vector*)) OR ((artificial* OR ambient* OR machine* OR algorithm* OR neural) NEAR/3 (intelligen* OR network*)) OR ((automat*) NEAR/3 (pattern*) NEAR/3 (recogni*)) OR ((learn*) NEAR/3 (algori*)) OR ((machine* OR deep OR supervis* OR unsupervis* OR bayes) NEAR/3 (learn* OR vector*))):ab,ti) AND ((hospital* OR clinic* OR ((intensiv* OR critical) NEAR/3 (care*)) OR IC OR ICU OR intramural* OR inpatient* OR radiolog* OR cardiolog* OR guideline*):ab,ti,kw) |
| *Google Scholar* | ethic\|ethics\|ethical\|moral\|immoral\|unethical\|bioethics AI\|"artificial\|machine intelligence"\|"machine\|deep learning" hospital\|clinic\|clinics\|clinical\|"intensive\|critical care"\|IC\|ICU\|intramural\|inpatient\|radiology\|cardiology\|guideline  ethic\|ethics\|ethical\|moral\|immoral\|unethical\|bioethics AI\| 'artificial\|machine intelligence '\| 'machine\|deep learning' hospital\|clinic\|clinics\|clinical\| 'intensive\|critical care '\|IC\|ICU\|intramural\|inpatient\|radiology\|cardiology\|guideline |

# eTable 2. WHO ethical principles

| *WHO ethical AI principle** | *Definition* | *Leading criteria* |
| --- | --- | --- |
| *Principle 1:*  *Protecting human autonomy* | AI in healthcare must not undermine human autonomy | - Humans must retain control over healthcare systems and decision-making - AI must assist healthcare providers and patients in making informed decisions. - Privacy, confidentiality, and informed consent must be protected. - Systems should enable meaningful human oversight and reversibility. |
| *Principle 2:*  *Promoting human well-being, safety and the public interest* | AI technologies should not harm people | - AI must comply with safety, accuracy, and efficacy standards before deployment. - Continuous quality control and monitoring are required. - Safeguards should prevent mental or physical harm, stigmatisation, or discrimination. |
| *Principle 3:*  *Ensuring transparency, explainability and intelligibility* | AI must be intelligible and understandable to stakeholders | - Transparency in AI design and deployment is essential. - Documentation of assumptions, data properties, and limitations must be available. - AI systems must be explainable to users and stakeholders, tailored to their capacities. - Tests and evaluations should be regular and transparent. |
| *Principle 4:*  *Fostering responsibility and accountability* | AI tasks must be under the responsibility of stakeholders | - Clearly define tasks and conditions for AI use. - Establish mechanisms for responsibility and accountability among developers, users, and regulators. - Provide effective remedies and redress for harm caused by AI-based decisions. - Avoid diffusion of responsibility with collective accountability frameworks. |
| *Principle 5:*  *Ensuring inclusiveness*  *and equity* | AI should be available, useful, and inclusive for all | - AI must not introduce or exacerbate biases or inequalities. - Training data should represent diverse populations to avoid discrimination. - Efforts should aim to reduce the digital divide and ensure access in low-resource settings. - AI should empower all stakeholders, including marginalised groups, to benefit equally. |
| *Principle 6:*  *Promoting AI that is responsive and sustainable* | AI must be sustainable and adaptable | - Continuously assess AI technologies for responsiveness to user needs. - Ensure alignment with health system sustainability and environmental goals. - Minimise ecological footprints and address workplace disruptions caused by automation. - Commit to updating, repairing, and integrating AI into under-resourced systems effectively. |

***** The definitions and leading criteria were formulated based on the World Health Organization’s Guidance on Ethics and Governance of AI for Health (2021).^1^ AI = Artificial intelligence; WHO = World Health Organization.

# eTable 3. Excluded publications with reasons for exclusion

| *No.* | *Author* | *Year of publication* | *Reason for exclusion* |
| --- | --- | --- | --- |
| *1* | Afnan, M, et al. | 2021 | Conference abstract |
| *2* | Al-Hindawi, A | 2023 | Conference abstract |
| *3* | Anastasi, A, et al. | 2023 | Conference abstract |
| *5* | Ashenburg, N, et al. | 2023 | Conference abstract |
| *6* | Brodie, A, et al. | 2021 | Conference abstract |
| *9* | Cagliero, D, et al. | 2021 | Conference abstract |
| *11* | Carter, S | 2020 | Conference abstract |
| *13* | Carter, S, et al. | 2019 | Conference abstract |
| *16* | Cavaciuti, M, et al. | 2020 | Conference abstract |
| *18* | Charalambides, M | 2019 | Conference abstract |
| *22* | Chen, D, et al. | 2022 | Conference abstract |
| *31* | Cheng, Q, et al. | 2018 | Conference abstract |
| *37* | Cherevko, A, et al. | 2022 | Conference abstract |
| *47* | Chneiweiss, H | 2019 | Conference abstract |
| *49* | Coulter, C, et al. | 2020 | Conference abstract |
| *56* | Currie, G, et al. | 2020 | Conference abstract |
| *69* | Danilatou, V, et al. | 2021 | Conference abstract |
| *79* | Desailly, E, et al. | 2019 | Conference abstract |
| *80* | Dina Radenkovic, D, et al. | 2019 | Conference abstract |
| *82* | Dinapoli, N, et al. | 2019 | Conference abstract |
| *89* | Fasterholdt, I, et al. | 2022 | Conference abstract |
| *91* | Hamer, M, et al. | 2023 | Conference abstract |
| *93* | Hoong, NK | 2021 | Conference abstract |
| *98* | Hoong, NK | 2019 | Conference abstract |
| *99* | Istasy, P, et al. | 2021 | Conference abstract |
| *100* | Javor, E | 2022 | Conference abstract |
| *104* | Jeste, D, et al. | 2019 | Conference abstract |
| *115* | Kazmierska, J | 2021 | Conference abstract |
| *118* | Kellmeyer, P | 2021 | Conference abstract |
| *122* | Kharb, S, et al. | 2021 | Conference abstract |
| *137* | Kollins, S, et al. | 2019 | Conference abstract |
| *139* | Lewis, S, et al. | 2021 | Conference abstract |
| *150* | Liu, X, et al. | 2018 | Conference abstract |
| *158* | Martinez-Martin, N | 2019 | Conference abstract |
| *172* | Matin, A, et al. | 2023 | Conference abstract |
| *175* | Mills, C, et al. | 2023 | Conference abstract |
| *180* | O'Donovan, T, et al. | 2022 | Conference abstract |
| *181* | Pinto Da Costa, M | 2020 | Conference abstract |
| *182* | Prada-Ramallal, G | 2022 | Conference abstract |
| *183* | Shah, SFH, et al. | 2023 | Conference abstract |
| *192* | Sinha, I, et al. | 2019 | Conference abstract |
| *196* | Sorin, V, et al. | 2022 | Conference abstract |
| *199* | Tampu, IE, et al. | 2023 | Conference abstract |
| *203* | Titovich, E | 2023 | Conference abstract |
| *204* | Tolias, Y | 2021 | Conference abstract |
| *206* | Urman, A, et al. | 2018 | Conference abstract |
| *207* | Van Lingen, M, et al. | 2023 | Conference abstract |
| *209* | Verbakel, W | 2019 | Conference abstract |
| *216* | Voskens, F, et al. | 2023 | Conference abstract |
| *229* | Wang, M, et al. | 2019 | Conference abstract |
| *240* | Woodward-Court, P, et al. | 2022 | Conference abstract |
| *247* | Yeager, B, et al. | 2020 | Conference abstract |
| *249* | Zaim, R, et al. | 2023 | Conference abstract |
| *251* | Anonymous | 2022 | Correction to a paper |
| *254* | Rogers, WA, et al. | 2022 | Correction to a paper |
| *272* | Walk, O, et al. | 2022 | Correction to a paper |
| *275* | Adlakha, S, et al. | 2020 | No full text available |
| *277* | Afnan, MAM, et al. | 2021 | No full text available |
| *278* | Ahmad, MA, et al. | 2021 | No full text available |
| *281* | Alkesijevic, A, et al. | 2022 | No full text available |
| *286* | Amugongo, LM, et al. | 2023 | No full text available |
| *289* | Aracena, C, et al. | 2022 | No full text available |
| *290* | Basu, T, et al. | 2020 | No full text available |
| *291* | Berner, ES | 2002 | No full text available |
| *296* | Cacciamani, GE, et al. | 2023 | No full text available |
| *300* | Gallone, G, et al. | 2022 | No full text available |
| *303* | Gandolfi, M, et al. | 2023 | No full text available |
| *310* | Gerke, S | 2024 | No full text available |
| *318* | Hui, AT, et al. | 2021 | No full text available |
| *320* | Ienca, M, et al. | 2020 | No full text available |
| *325* | Kernbach, JM, et al. | 2022 | No full text available |
| *326* | Kim, JY, et al. | 2023 | No full text available |
| *328* | Lo, B | 2022 | No full text available |
| *330* | Makimoto, H, et al. | 2023 | No full text available |
| *331* | Mathiesen, T, et al. | 2022 | No full text available |
| *332* | McGregor, C | 2021 | No full text available |
| *334* | Milosevic, Z | 2021 | No full text available |
| *344* | Mishra, A | 2019 | No full text available |
| *349* | Mishra, NK, et al. | 2022 | No full text available |
| *351* | Mishra, NK, et al. | 2022 | No full text available |
| *357* | Mittelstadt, B | 2019 | No full text available |
| *362* | Nct | 2021 | No full text available |
| *363* | Ng, WY, et al. | 2021 | No full text available |
| *366* | Nolan, P | 2023 | No full text available |
| *367* | Nsier, H | 2023 | No full text available |
| *369* | Peine, A, et al. | 2020 | No full text available |
| *370* | Prajapati, JB, et al. | 2022 | No full text available |
| *371* | Rigby, MJ, et al. | 2019 | No full text available |
| *373* | Rogers, WA, et al. | 2021 | No full text available |
| *374* | Rossi, PJ, et al. | 2014 | No full text available |
| *376* | Samyuktha, PS, et al. | 2020 | No full text available |
| *391* | Schueller, SM, et al. | 2023 | No full text available |
| *395* | Seitzinger, P, et al. | 2021 | No full text available |
| *398* | Sendak, M, et al. | 2020 | No full text available |
| *402* | Stanley, EAM, et al. | 2022 | No full text available |
| *409* | Tarek, Z, et al. | 2023 | No full text available |
| *412* | Valtchinov, VI, et al. | 2021 | No full text available |
| *413* | Wang, H, et al. | 2023 | No full text available |
| *419* | Wu, Y, et al. | 2023 | No full text available |
| *426* | Yamada, H, et al. | 2015 | No full text available |
| *430* | Bin Zahid, A, et al. | 2023 | No specific mention of ethical considerations related to WHO principles |
| *432* | Bourla, A, et al. | 2018 | No specific mention of ethical considerations related to WHO principles |
| *440* | Daidone, M, et al. | 2024 | No specific mention of ethical considerations related to WHO principles |
| *441* | El-Bahnasawi, M, et al. | 2019 | No specific mention of ethical considerations related to WHO principles |
| *442* | Heidari, N, et al. | 2024 | No specific mention of ethical considerations related to WHO principles |
| *449* | Jin, B, et al. | 2020 | No specific mention of ethical considerations related to WHO principles |
| *452* | Mir, MA, et al. | 2023 | No specific mention of ethical considerations related to WHO principles |
| *453* | Motta, O, et al. | 2020 | No specific mention of ethical considerations related to WHO principles |
| *460* | Ray, PP | 2023 | No specific mention of ethical considerations related to WHO principles |
| *466* | Rodriguez-Merchan, EC | 2022 | No specific mention of ethical considerations related to WHO principles |
| *490* | Senapati, SG, et al. | 2023 | No specific mention of ethical considerations related to WHO principles |
| *492* | Wu, X, et al. | 2020 | No specific mention of ethical considerations related to WHO principles |
| *498* | Ahmed, L, et al. | 2023 | No use of artificial intelligence |
| *508* | Alegria, JR, et al. | 2023 | No use of artificial intelligence |
| *512* | Aminizadeh, S, et al. | 2023 | No use of artificial intelligence |
| *520* | Car, J, et al. | 2019 | No use of artificial intelligence |
| *525* | Erdmann, A, et al. | 2021 | No use of artificial intelligence |
| *526* | Finocchiaro, G, et al. | 2022 | No use of artificial intelligence |
| *533* | Jamjoom, AAB, et al. | 2022 | No use of artificial intelligence |
| *537* | Ligthart, S, et al. | 2023 | No use of artificial intelligence |
| *538* | Mumtaz, H, et al. | 2023 | No use of artificial intelligence |
| *562* | Yuste, R | 2023 | No use of artificial intelligence |
| *564* | Calvo, P | 2019 | Non-English |
| *566* | Kulzer, B | 2021 | Non-English |
| *579* | Perin, A | 2019 | Non-English |
| *585* | Wilhelm, D, et al. | 2022 | Non-English |
| *587* | Abramoff, MD, et al. | 2020 | Not inpatient healthcare |
| *590* | Alabi, RO et al. | 2021 | Not inpatient healthcare |
| *594* | Arshad, HB, et al. | 2023 | Not inpatient healthcare |
| *596* | Blease, C, et al. | 2020 | Not inpatient healthcare |
| *598* | Boczar, D, et al. | 2021 | Not inpatient healthcare |
| *602* | Briganti, G, et al. | 2020 | Not inpatient healthcare |
| *610* | Brigden, T, et al. | 2023 | Not inpatient healthcare |
| *618* | Chamunyonga, C, et al. | 2020 | Not inpatient healthcare |
| *619* | Chen, J, et al. | 2020 | Not inpatient healthcare |
| *626* | Ducret, M, et al. | 2022 | Not inpatient healthcare |
| *628* | Flores, L, et al. | 2023 | Not inpatient healthcare |
| *639* | Frehywot, S, et al. | 2023 | Not inpatient healthcare |
| *641* | Fu, S, et al. | 2023 | Not inpatient healthcare |
| *650* | Grzybowski, A, et al. | 2023 | Not inpatient healthcare |
| *659* | Hallowell, N, et al. | 2023 | Not inpatient healthcare |
| *665* | Jenkinson, GP, et al. | 2023 | Not inpatient healthcare |
| *667* | Karakas, C, et al. | 2023 | Not inpatient healthcare |
| *682* | Kong, JD, et al. | 2023 | Not inpatient healthcare |
| *691* | Malerbi, FK, et al. | 2023 | Not inpatient healthcare |
| *704* | Nebeker, C, et al. | 2019 | Not inpatient healthcare |
| *706* | Parikh, RB, et al. | 2022 | Not inpatient healthcare |
| *708* | Rigberg, DA, et al. | 2023 | Not inpatient healthcare |
| *710* | Sawers, N, et al. | 2021 | Not inpatient healthcare |
| *711* | Schwalbe, N, et al. | 2020 | Not inpatient healthcare |
| *717* | Sridi, C, et al. | 2023 | Not inpatient healthcare |
| *734* | Temsah, MH, et al. | 2023 | Not inpatient healthcare |
| *753* | Zecca, PA, et al. | 2023 | Not inpatient healthcare |
| *761* | Alabi, RO, et al. | 2020 | Not peer reviewed |
| *762* | Berbís, MA, et al. | 2022 | Not peer reviewed |
| *765* | Blasimme, A, et al. | 2019 | Not peer reviewed |
| *775* | Briguglio, L, et al. | 2023 | Not peer reviewed |
| *780* | Capelli, G, et al. | 2023 | Not peer reviewed |
| *781* | Gerke, S, et al. | 2020 | Not peer reviewed |
| *785* | Joyce, D, et al. | 2023 | Not peer reviewed |
| *787* | Morley, J, et al. | 2019 | Not peer reviewed |
| *792* | Pasricha, S | 2023 | Not peer reviewed |
| *813* | Sallam, M. | 2023 | Not peer reviewed |
| *816* | Vollmer, S, et al. | 2018 | Not peer reviewed |
| *822* | World Health Organization | 2021 | Used as framework in this study |

eTable 4. Data extraction form
*The data options available per data extraction subject.*

| **Data extraction subject** | **Data options** |
| --- | --- |
| **Year of publication** | Free text |
| **Country of publication** | Free text |
| **Study design** | - Descriptive - Experimental - Observational - Qualitative - Mixed-methods |
| **Type of paper** | - Review - Review protocol - Original research article - Case report - Consensus statement - Framework - Research protocol - Expert discussion - Perspective - Viewpoint - Commentary - Correspondence letter - Editorial - Essay - Letter to the editor - White paper |
| **Domain*** | - Medical domain - Technical domain - Ethics domain - Policy domain |
| **Medical specialty** | - Free text |
| **AI type** | - General AI - Machine learning - Deep learning - Generative AI - Natural language processing - Reinforcement learning - Federated learning |
| **AI readiness level†** | - TRL 1 - TRL 2 - TRL 3-4 - TRL 5 - TRL 6 - TRL 7 - TRL 8 - TRL 9 - N/A |

***** **Domains** were classified as follows: the **medical domain** includes studies focusing on the end-user, such as patients or healthcare professionals, either as the target audience or central topic. The **technical domain** includes studies where the target audience consists of engineers or AI developers, or where the main focus is model development and or technical analyses. The **ethics domain** includes studies primarily targeting ethicists or philosophers, or those where the main objective is an ethical analysis of AI in healthcare. The **policy domain** includes studies addressing policymakers or legal experts as the target audience, or those focusing on legal and regulatory aspects of AI, including AI governance and policymaking. **†** **AI readiness level** was classified using the **Technology Readiness Level (TRL) scale**. For studies that did not focus on developing or testing a specific AI model, TRL classification was not applicable. AI = Artificial Intelligence; N/A = not applicable.

# eTable 5. Technology Readiness Level (TRL) scale

*TRL scale was used to assign a level of readiness to included studies describing model development or testing in a clinical context.*

| *TRL** | *Clinical definition* | *Explanation* |
| --- | --- | --- |
| *1* | Clinical problem identification | Identification of potential medical machine learning solution to defined clinical problem. |
| *2* | Proposal of model/solution | Elaborate proposal of potential medical machine learning solution to clinical problem. Medical dataset identified and outcomes defined. |
| *3&4* | Model prototyping & Model development | Prototype model or model development. Demonstrating potential for prediction or decision support or optimizing model and validation on characterised data. |
| *5* | Model validation | Representative model is validated on realistic dataset other than original training and testing population (ideally data pre-processing and cleaning pipelines are specified for all data sources). |
| *6* | Real-time model testing | Model performance is tested real-time and integrated into the EHR system in one or more clinical settings, but no implementation into clinical workflow (i.e. no clinical staff is exposed to model results). |
| *7* | Workflow implementation | Model is implemented in clinical workflow (i.e. clinical staff is exposed to model results). Performance, safety, and effects on patient relevant outcomes are assessed. Workability by clinical staff is evaluated. Comparable with a ‘Phase 2’ Study. |
| *8* | Clinical outcome evaluation | Implemented model evaluation against clinical outcomes. Comparable with a ‘Phase 3’ Study. |
| *9* | Model integration | Model has proven to work in a research setting in its final form and in expected conditions. Model is integrated in the clinical workflow and evaluated in different centres (if appropriate) whether it meets specifications. |

***** Clinical definitions and explanations were adapted from Fleuren et al.^2^ AI = Artificial intelligence; TRL = Technology Readiness Level.

# eTable 6. Standardised list of relevant keywords per WHO ethical principle

*The presence of a keyword alone was insufficient; it had to be clearly discussed in an ethical context. Other variations of keywords (e.g., "explainable "inequality," "sustainable") were also included.*

| *WHO ethical AI principle* | *Relevant keywords* |
| --- | --- |
| *Principle 1:*  *Protecting human autonomy* | Autonomy, confidentiality, human oversight,  informed consent, privacy |
| *Principle 2:*  *Promoting human well-being, safety and the public interest* | Beneficence, efficacy, harm, non-maleficence, public interest, safety, well-being |
| *Principle 3:*  *Ensuring transparency, explainability and intelligibility* | Explainability, explicability, intelligibility, interpretability, transparency, understandability |
| *Principle 4:*  *Fostering responsibility and accountability* | Accountability, Contestability, human oversight, human supervision, liability, redress, responsibility |
| *Principle 5:*  *Ensuring inclusiveness*  *and equity* | Bias, discrimination, disparities, equity, fairness, inclusiveness, equality, justice |
| *Principle 6:*  *Promoting AI that is responsive and sustainable* | Carbon, climate, continuous improvement, continuous monitoring, ecological footprint, ecosystem, energy efficiency, environment, responsiveness, sustainability |

WHO = World Health Organization; AI = Artificial Intelligence.

# eTable 7. Criteria for evaluating degree of consideration

*A distinction was made between in-depth consideration and brief mention of WHO ethical principles. A study was categorised as either “in-depth consideration” or “brief mention” if it met at least one of the specified criteria within that classification. Additionally, examples of “brief mention” for non-original studies also apply to original studies, particularly when ethical principles where referenced briefly in the introduction or discussion.*

| **Classification** | **Criteria** |
| --- | --- |
| **In-depth consideration** | **Non-original studies**  **•** Provides extensive elaboration or illustrative examples of the relevant “keyword,” including potential risks (“what can go wrong?”) or strategies (“what could be done?”) to promote the principle. |
|  | **Original studies** • Discusses the relevant “keyword” in the introduction or discussion *with extensive elaboration*, such as addressing potential risks or strategies.  • Describes in the methods or results how the study *actively incorporated* the ethical principle in AI design, development, evaluation, or implementation. |
| **Brief mention** | **Non-original studies** • References a framework or regulation focusing on the “keyword” *without elaboration*. • States that the “keyword” must be considered in the use of AI for healthcare *without providing specific details*. • Notes that the current landscape focuses on the “keyword” *without examples or further discussion*. • Mentions the “keyword” in passing, but the main focus is a *different* WHO principle. • Claims a particular aspect *may* lead to “keyword”-related issues, *without* further context. • Suggests that *future research* should focus on the “keyword.” |
|  | **Original studies** • *Briefly* mentions the relevance of the “keyword” (e.g., in the introduction or discussion) but *without further detail*, as in examples provided for non-original studies. |

WHO = World Health Organization.

eTable 8. Keyword presence and the level of consideration given to each principle by the included publications
*LOC = level of consideration. ✔ = in-depth consideration, ~ = brief mention.*

| **No.** | **Authors** | **Year** | **Keywords**  **WHO 1** | **LOC WHO 1** | **Keywords WHO 2** | **LOC WHO 2** | **Keywords  WHO 3** | **LOC WHO 3** | **Keywords  WHO 4** | **LOC WHO 4** | **Keywords WHO 5** | **LOC WHO 5** | **Keywords**  **WHO 6** | **LOC WHO 6** |
| --- | --- | --- | --- | --- | --- | --- | --- | --- | --- | --- | --- | --- | --- | --- |
| **4** | Currie, GM | 2023 | Informed consent; | ~ |  |  | Interpretability; | ✔ | Liability; | ✔ | Bias ; Equity; | ✔ |  |  |
| **7** | Migliorelli, L, et al. | 2023 | Human oversight; Privacy; | ~ | Harm; Safety; Well-being; | ~ | Transparency; Explainability; | ~ | Accountability; Redress; | ✔ | Fairness; Equality; Disparity; | ~ | Carbon; Environment ; Sustainability; | ~ |
| **8** | Gary, PJ, et al. | 2023 | Autonomy; Privacy; | ~ | Beneficence; Harm; Non-maleficence; | ~ | Transparency; | ~ | Responsibility; | ~ | Bias ; Discrimination; Justice; | ~ |  |  |
| **10** | Oniani, D, et al. | 2023 | Autonomy; Confidentiality; Privacy; | ✔ | Harm; Public interest; Safety; Well-being; | ✔ | Explainability; Interpretability; Transparency; Understandability; | ✔ | Accountability; Liability; Responsibility; | ✔ | Bias ; Discrimination; Disparity; Equity; Fairness; Inclusiveness; | ✔ | Responsiveness; Sustainability; | ✔ |
| **12** | Hickman, SE, et al. | 2021 |  |  | Harm; Safety; | ✔ | Transparency; | ✔ | Accountability; Human oversight; Human supervision; Responsibility; | ✔ | Bias ; Discrimination; Fairness; Equality; | ✔ | Continuous monitoring ; Sustainability; | ✔ |
| **14** | Karmakar, A, et al. | 2023 | Privacy; | ✔ | Beneficence; Efficacy; Safety; | ✔ | Transparency; | ~ | Accountability; Responsibility; | ✔ | Bias ; Fairness; | ✔ | Sustainability; | ~ |
| **15** | Polevikov, S | 2023 | Autonomy; Confidentiality; Informed consent; Privacy; | ✔ | Efficacy; Harm; Public interest; Safety; Well-being; | ✔ | Explainability; Interpretability; Transparency; | ✔ | Accountability; Responsibility; | ~ | Bias ; Discrimination; Disparity; Equality; Equity; Justice; | ✔ | Environment; Responsiveness;  Sustainability; | ~ |
| **17** | Sorin, V, et al. | 2023 | Human oversight; | ✔ | Safety; | ✔ |  |  |  |  |  |  |  |  |
| **19** | Kawamleh, S, et al. | 2022 | Autonomy; Informed consent; | ✔ | Harm; Safety; | ✔ | Explainability; Interpretability; Transparency; Understandability; | ✔ |  |  | Bias ; Fairness; | ~ |  |  |
| **20** | Groza, A, et al. | 2021 |  |  | Safety; | ✔ | Explainability; Transparency; | ✔ |  |  |  |  |  |  |
| **21** | Drabiak, K, et al. | 2023 | Privacy; Informed consent; | ✔ | Efficacy; Harm; Safety; Well-being; | ✔ | Explainability; Interpretability; Transparency; | ✔ | Accountability; Liability; | ✔ | Bias ; Fairness; Equality; Equity; | ✔ | Sustainability; | ~ |
| **23** | Fenech, ME, et al. | 2020 | Autonomy; Informed consent; | ✔ | Safety; | ~ | Explainability; Transparency; | ✔ | Liability; Responsibility; | ✔ | Bias ; Fairness; Equality; | ✔ | Responsiveness; | ~ |
| **24** | Rajpurkar, P, et al. | 2022 | Autonomy; Informed consent; Privacy; | ✔ |  |  | Explainability; Interpretability; Transparency; | ✔ | Accountability; Liability; Responsibility; | ✔ | Bias ; Equity; Fairness; Equity; | ✔ |  |  |
| **25** | Mello-Thoms, C, et al. | 2023 | Privacy; | ✔ | Harm; | ~ | Explainability; Interpretability; Transparency; Understandability; | ✔ | Liability; Responsibility; | ✔ | Bias ; Disparity; Equality; | ~ |  |  |
| **26** | Khera, R, et al. | 2023 |  |  | Safety; | ~ |  |  |  |  | Bias ; Disparity; Equity; Equity; | ✔ |  |  |
| **27** | Ng, MY, et al. | 2022 | Privacy; | ✔ | Efficacy; Harm; | ✔ | Interpretability; Transparency; | ✔ |  |  | Bias ; Discrimination; Equality; Fairness; Equity; | ✔ |  |  |
| **28** | Biller-Andorno, N, et al. | 2022 |  |  | Harm; | ~ | Explainability; Interpretability; Transparency; | ✔ | Accountability; Liability; | ✔ | Bias ; Disparity; Fairness; | ✔ |  |  |
| **29** | Lysaght, T, et al. | 2019 | Informed consent; | ~ | Harm; Public interest; | ✔ | Explainability; Interpretability; Transparency; | ✔ | Accountability; Liability; Responsibility; | ✔ | Bias ; Discrimination; Equity; Equity; Justice; | ✔ |  |  |
| **30** | De Panfilis, L, et al. | 2023 | Autonomy; Privacy; Human oversight; | ✔ |  |  | Interpretability; | ~ | Liability; Responsibility; | ✔ | Bias ; Discrimination; Equality; | ✔ |  |  |
| **32** | Johnson, SLJ | 2019 | Autonomy; Confidentiality; Informed consent; Privacy; | ✔ | Beneficence; Harm; Non-maleficence; Safety; Well-being; | ✔ | Explainability; Interpretability; Transparency; Understandability; | ✔ | Accountability; Responsibility; | ✔ | Bias ; Discrimination; Disparity; Equity; Fairness; Justice; | ✔ |  |  |
| **33** | Biller-Andorno, N, et al. | 2019 | Autonomy; Privacy; | ~ | Safety; | ~ | Explainability; Transparency; | ✔ | Accountability; Liability; | ~ | Bias ; Discrimination; | ~ |  |  |
| **34** | Yang, J, et al. | 2023 | Privacy; | ~ | Harm; | ~ |  |  |  |  | Bias ; Discrimination; Disparity; Equity; Fairness; Equity; | ✔ |  |  |
| **35** | Meier, LJ, et al. | 2022 | Autonomy; | ✔ | Beneficence; Harm; Non-maleficence; | ✔ | Interpretability; Transparency; | ✔ | Accountability; | ~ | Bias ; Discrimination; Fairness; Inclusiveness; Justice; | ✔ |  |  |
| **36** | Hogberg, C, et al. | 2023 |  |  | Harm; | ~ | Interpretability; Transparency; | ✔ | Accountability; Liability; Responsibility; | ✔ | Fairness; | ~ |  |  |
| **38** | Gutierrez, L, et al. | 2022 | Privacy; | ~ | Efficacy; | ~ | Explainability; Transparency; | ✔ |  |  |  |  |  |  |
| **39** | Wu, J, et al. | 2021 | Privacy; | ~ |  |  |  |  | Accountability; | ~ |  |  |  |  |
| **40** | Zhang, Z, et al. | 2021 |  |  |  |  | Interpretability; | ~ |  |  |  |  |  |  |
| **41** | Sonawane, A, et al. | 2023 | Confidentiality; Informed consent; Privacy; | ✔ | Safety; | ~ | Transparency; | ~ | Accountability; Responsibility; | ~ | Bias; | ~ |  |  |
| **42** | Schukow, C, et al. | 2024 | Confidentiality; Privacy; | ✔ | Safety; | ~ | Interpretability; Transparency; Understandability; | ✔ |  |  | Bias; | ~ |  |  |
| **43** | Casey, A, et al. | 2023 | Privacy; | ✔ | Non-maleficence; Safety; | ✔ | Interpretability; Transparency; | ✔ |  |  | Bias ; Fairness; | ~ |  |  |
| **44** | Mikdadi, D, et al. | 2022 |  |  |  |  | Explainability; Interpretability; Transparency; Understandability; | ✔ |  |  | Bias ; Discrimination; Fairness; Equality; | ✔ |  |  |
| **45** | Xu, J, et al. | 2022 | Privacy; | ~ | Safety; | ~ | Interpretability; Transparency; | ✔ | Accountability; | ~ |  |  |  |  |
| **46** | Zhang, C, et al. | 2022 | Privacy; | ~ |  |  |  |  | Responsibility; | ~ |  |  |  |  |
| **48** | Chan, L, et al. | 2020 | Confidentiality; Privacy; | ~ | Harm; Safety; | ~ | Interpretability; | ~ |  |  | Bias; | ~ |  |  |
| **50** | Mysona, DP, et al. | 2021 | Privacy; | ~ |  |  | Interpretability; Transparency; | ~ | Liability; Responsibility; | ~ | Bias; | ~ | Continuous improvement; | ~ |
| **51** | Suominen, H, et al. | 2007 | Autonomy; Confidentiality; Privacy; | ✔ | Harm; Safety; | ✔ |  |  | Liability; Responsibility; | ✔ |  |  |  |  |
| **52** | Farah, L, et al. | 2023 | Privacy; | ~ | Harm; Safety; | ✔ | Explainability; Intelligibility; Interpretability; Transparency; Understandability; | ✔ |  |  | Bias; | ~ |  |  |
| **53** | Sahin, E | 2023 |  |  | Efficacy; Well-being; | ✔ |  |  | Liability; | ~ |  |  |  |  |
| **54** | von Gerich, H, et al. | 2022 | Autonomy; Privacy; | ~ | Harm; Safety; | ~ | Transparency; | ~ |  |  | Bias ; Discrimination; Justice; | ~ |  |  |
| **55** | Higgins, O, et al. | 2023 | Autonomy; | ~ | Harm; Safety; Well-being; | ✔ | Intelligibility; Interpretability; Transparency; Understandability; | ✔ | Accountability; Responsibility; | ✔ | Bias ; Fairness; Discrimination; | ~ |  |  |
| **57** | Lamb, LR, et al. | 2022 |  |  | Safety; | ~ | Interpretability; Explainability; Transparency; | ✔ | Liability; Responsibility; | ✔ | Bias ; Disparity; Equity; | ✔ | Continuous monitoring; | ✔ |
| **58** | Bond, A, et al. | 2023 | Privacy; | ✔ | Harm; Safety; | ✔ | Interpretability; Transparency; Understandability; | ✔ | Accountability; Liability; Responsibility; | ✔ | Bias ; Discrimination; | ✔ |  |  |
| **59** | Zafar, S, et al. | 2022 | Privacy; | ✔ | Efficacy; Safety; | ~ | Explainability; Transparency; | ✔ | Liability; | ~ | Bias ; Equality; Equity; | ✔ |  |  |
| **60** | Letterie, G | 2023 |  |  | Safety; | ~ |  |  |  |  |  |  |  |  |
| **61** | London, AJ | 2019 | Autonomy; | ✔ | Harm; | ✔ | Explainability; Interpretability; Transparency; Understandability; | ✔ | Accountability; Responsibility; | ✔ | Bias; | ✔ |  |  |
| **62** | Cui, M, et al. | 2021 | Privacy; | ~ | Safety; | ~ |  |  |  |  |  |  |  |  |
| **63** | Jin, K, et al. | 2022 | Privacy; | ~ |  |  | Explainability; Explicability; Interpretability; Transparency; Understandability; | ✔ |  |  | Bias ; Discrimination; Equality; | ✔ |  |  |
| **64** | Alami, H, et al. | 2020 | Autonomy; Confidentiality; | ✔ | Harm; Well-being; | ~ | Interpretability; Transparency; Understandability; | ✔ | Responsibility; | ✔ | Bias ; Disparity; Equity; | ✔ | Sustainability; | ~ |
| **65** | Asan, O, et al. | 2020 |  |  | Efficacy; Safety; | ✔ | Explainability; Transparency; Understandability; | ✔ | Responsibility; | ✔ | Bias ; Discrimination; Fairness; | ✔ |  |  |
| **66** | Sollini, M, et al. | 2020 | Confidentiality; Privacy; | ✔ | Efficacy; | ~ | Explainability; Interpretability; Transparency; Understandability; | ✔ | Accountability; | ~ | Bias ; Discrimination; Fairness; | ~ | Sustainability; | ~ |
| **67** | Chockalingam, A, et al. | 2021 | Privacy; | ✔ |  |  | Explainability; Understandability; Transparency; | ✔ |  |  | Bias ; Equity; | ✔ |  |  |
| **68** | Balsano, C, et al. | 2023 | Privacy; | ✔ | Safety; Well-being; | ✔ | Explainability; Transparency; Understandability; | ✔ | Accountability; Liability; Responsibility; | ✔ | Bias; | ✔ |  |  |
| **70** | Galbusera, F, et al. | 2019 | Informed consent; Privacy; | ✔ |  |  | Interpretability; Understandability; | ✔ | Accountability; Liability; Responsibility; | ✔ | Bias; | ✔ |  |  |
| **71** | Nelson, B, et al. | 2023 |  |  |  |  | Transparency; | ~ |  |  | Bias ; Fairness; Equity; Equality; | ✔ |  |  |
| **72** | Wamala-Andersson, S, et al. | 2023 | Autonomy; | ~ | Public interest; Safety; Well-being; | ~ | Explainability; Intelligibility; Transparency; | ~ | Accountability; Responsibility; | ~ | Bias ; Equity; Inclusiveness; | ✔ |  |  |
| **73** | Ergin, E, et al. | 2022 | Privacy; | ~ | Harm; Safety; | ✔ |  |  | Responsibility; | ✔ | Justice; | ~ |  |  |
| **74** | Hernigou, P, et al. | 2023 | Privacy; | ~ |  |  |  |  | Responsibility; | ✔ | Bias; | ~ |  |  |
| **75** | Watson, NF, et al. | 2021 |  |  | Harm; | ~ | Interpretability; Transparency; Understandability; | ✔ |  |  | Disparity; | ✔ |  |  |
| **76** | Cobianchi, L, et al. | 2022 | Autonomy; Human oversight; Privacy; | ✔ | Harm; Non-maleficence; Safety; Well-being; | ✔ | Explainability; Explicability; Transparency; | ✔ | Accountability; Liability; Responsibility; | ✔ | Bias ; Discrimination; Disparity; Equality; Equity; Fairness; Inclusiveness; | ✔ | Sustainability; | ~ |
| **77** | Loftus, TJ, et al. | 2020 | Informed consent; | ~ | Harm; Safety; | ✔ | Interpretability; Understandability; | ✔ | Accountability; Responsibility; | ~ | Bias ; Discrimination; | ✔ |  |  |
| **78** | Lorenzini, G, et al. | 2023 | Autonomy; Informed consent; | ✔ | Harm; Safety; | ~ | Explainability; Transparency; Understandability; | ✔ | Accountability; Responsibility; | ✔ |  |  |  |  |
| **81** | Shaw, J, et al. | 2019 | Autonomy; Privacy; | ✔ | Safety; Public interest; | ~ | Explainability; | ✔ |  |  | Bias ; Equity; | ✔ |  |  |
| **83** | Spanos, K, et al. | 2022 | Autonomy; Confidentiality; Informed consent; Privacy; | ~ | Beneficence; Non-maleficence; Safety; | ~ | Explicability; Intelligibility; Transparency; | ✔ | Accountability; Liability; Responsibility; | ~ | Bias; | ~ |  |  |
| **84** | Rokhshad, R, et al. | 2023 |  |  |  |  |  |  |  |  | Bias ; Discrimination; | ~ |  |  |
| **85** | Weerarathna, IN, et al. | 2023 | Privacy; | ~ | Safety; | ~ | Interpretability; | ~ |  |  | Bias ; Fairness; | ~ |  |  |
| **86** | Sharma, M, et al. | 2022 | Autonomy; | ✔ | Safety; | ~ | Explainability; Interpretability; Transparency; Understandability; | ✔ | Accountability; Liability; | ~ |  |  | Sustainability; | ~ |
| **87** | Schattenberg, JM, et al. | 2023 |  |  | Safety; | ✔ | Explainability; Interpretability; Transparency; Understandability; | ~ | Liability; Responsibility; | ~ | Bias ; Disparity; Equity; Equity; | ✔ |  |  |
| **88** | You, E, et al. | 2020 | Confidentiality; Privacy; | ~ |  |  |  |  |  |  | Bias; | ~ |  |  |
| **90** | Pesapane, F, et al. | 2018 | Confidentiality; Privacy; | ✔ | Harm; Public interest; Safety; | ~ | Transparency; Understandability; | ~ | Accountability; Liability; Responsibility; | ✔ | Bias ; Discrimination; | ✔ |  |  |
| **92** | Dashwood, M, et al. | 2021 | Privacy; | ~ | Efficacy; Safety; | ~ | Explainability; | ~ | Accountability; | ~ | Discrimination; | ~ |  |  |
| **94** | Farasati Far, B | 2023 | Autonomy; Informed consent; Privacy; | ✔ | Harm; Safety; | ~ | Explainability; Transparency; Understandability; | ~ | Accountability; | ~ | Bias ; Discrimination; Equity; Equality; | ✔ |  |  |
| **95** | Turkbey, B, et al. | 2022 | Informed consent; | ✔ | Efficacy; Safety; | ~ | Transparency; Understandability; | ✔ |  |  | Bias; | ✔ |  |  |
| **96** | Cascella, M, et al. | 2023 | Autonomy; Privacy; | ~ |  |  | Explainability; Transparency; Understandability; | ✔ | Accountability; | ~ | Bias; | ~ |  |  |
| **97** | Pedersen, M, et al. | 2020 | Privacy; | ~ | Safety; Well-being; | ~ | Explainability; Interpretability; Transparency; Understandability; | ✔ | Accountability; Responsibility; | ~ | Bias ; Discrimination; Fairness; | ~ | Environment; | ~ |
| **101** | Sung, JJY, et al. | 2023 | Autonomy; Confidentiality; Informed consent; Privacy; | ✔ | Efficacy; Harm; Public interest; Safety; Well-being; | ~ | Explainability; Intelligibility; Interpretability; Transparency; Understandability; | ✔ | Accountability; Liability; Responsibility; | ✔ | Bias ; Discrimination; Disparity; Equity; Inclusiveness; Justice; | ✔ | Continuous improvement; Continuous monitoring; | ✔ |
| **102** | Murphy, K, et al. | 2021 | Autonomy; Confidentiality; Privacy; | ✔ | Harm; Safety; Well-being; | ✔ | Explainability; Transparency; Understandability; | ✔ | Accountability; Redress; Responsibility; | ✔ | Bias ; Discrimination; Equality; Equity; Fairness; Inclusiveness; | ✔ | Responsiveness; | ~ |
| **103** | Lee, EE, et al. | 2021 | Human oversight; | ~ | Safety; Well-being; | ~ | Explainability; Interpretability; Transparency; Understandability; | ~ |  |  | Bias ; Equality; Equity; Fairness; | ✔ | Sustainability; | ~ |
| **105** | Gheysen, F, et al. | 2023 |  |  | Efficacy; Safety; Well-being; | ✔ | Transparency; | ✔ | Accountability; | ~ | Bias; | ~ |  |  |
| **106** | Kellmeyer, P | 2021 | Autonomy; Privacy; | ✔ | Well-being; | ~ | Interpretability; | ~ | Accountability; Liability; Responsibility; | ✔ | Bias ; Discrimination; Equality; Fairness; Inclusiveness; Justice; | ✔ |  |  |
| **107** | Ozcan, BB, et al. | 2023 | Privacy; | ✔ | Harm; Safety; Well-being; | ~ | Interpretability; Transparency; Understandability; | ✔ | Accountability; Responsibility; | ~ | Bias ; Discrimination; Discrimination; Disparity; Equality; Fairness; | ✔ | Continuous monitoring; | ~ |
| **108** | Vedantham, S, et al. | 2023 |  |  | Efficacy; | ~ | Interpretability; | ~ | Liability; | ~ |  |  |  |  |
| **109** | Majumder, A, et al. | 2021 | Informed consent; Privacy; | ✔ | Safety; | ~ | Transparency; Understandability; | ✔ | Liability; | ~ | Bias ; Disparity; | ✔ |  |  |
| **110** | Jaltotage, B, et al. | 2023 | Confidentiality; Privacy; | ✔ | Efficacy; Safety; | ~ | Explainability; Interpretability; Transparency; Understandability; | ✔ | Liability; | ✔ | Bias ; Equality; | ✔ |  |  |
| **111** | Danilov, A, et al. | 2023 | Privacy; | ✔ | Efficacy; Harm; | ✔ | Explainability; Interpretability; Transparency; Understandability; | ✔ | Accountability; Liability; Responsibility; | ~ | Bias ; Discrimination; Fairness; Equality; Equity; | ✔ |  |  |
| **112** | Lopez-Jimenez, F, et al. | 2020 |  |  | Efficacy; Safety; | ~ |  |  |  |  | Bias ; Disparity; Equity; | ✔ |  |  |
| **113** | Lang, M, et al. | 2022 | Confidentiality; Privacy; | ~ | Efficacy; Harm; Safety; | ✔ | Explainability; Intelligibility; Interpretability; Understandability; | ✔ | Liability; Responsibility; | ✔ | Bias ; Fairness; | ~ |  |  |
| **114** | Smith, H, et al. | 2023 |  |  | Beneficence; Harm; Non-maleficence; Safety; | ~ | Explainability; Interpretability; Transparency; Understandability; | ✔ | Accountability; Liability; Responsibility; | ✔ |  |  |  |  |
| **116** | Kumar, KS, et al. | 2023 | Autonomy; Privacy; | ~ | Safety; | ~ | Explainability; Interpretability; Transparency; | ✔ | Accountability; | ~ | Bias ; Equity; | ✔ | Sustainability; | ~ |
| **117** | Rubinic, I, et al. | 2023 | Autonomy; Informed consent; Privacy; | ~ | Beneficence; Harm; Non-maleficence; Safety; Well-being; | ✔ | Explainability; Intelligibility; Interpretability; Transparency; | ✔ | Accountability; Contestability; Human oversight; Human supervision; Redress; Responsibility; | ~ | Discrimination; Fairness; Justice; | ~ |  |  |
| **119** | Singh, G | 2023 | Autonomy; | ~ | Safety; | ~ | Interpretability; Transparency; | ✔ | Liability; | ~ |  |  |  |  |
| **120** | Saqib, M, et al. | 2023 | Autonomy; Confidentiality; Human oversight; Informed consent; Privacy; | ✔ | Efficacy; Safety; | ~ | Explainability; Interpretability; Interpretability; Transparency; Understandability; | ✔ |  |  | Bias; | ~ |  |  |
| **121** | Young, AT, et al. | 2020 |  |  | Efficacy; | ~ | Interpretability; Understandability; | ✔ |  |  | Bias ; Discrimination; Disparity; Equity; | ✔ |  |  |
| **123** | Sengupta, D | 2023 | Privacy; | ~ | Safety; | ~ | Explainability; Interpretability; Transparency; | ✔ | Responsibility; | ~ | Bias; | ~ |  |  |
| **124** | Sehly, A, et al. | 2022 |  |  | Efficacy; | ~ | Understandability; | ~ | Human supervision;  Liability; | ~ |  |  |  |  |
| **125** | Vearrier, L, et al. | 2022 | Autonomy; Confidentiality; Privacy; | ✔ | Harm; Safety; | ~ | Transparency; | ~ | Liability; | ✔ | Bias; | ✔ |  |  |
| **126** | Grant, K, et al. | 2020 | Privacy; | ✔ | Safety; | ~ | Transparency; Understandability; | ✔ | Liability; | ✔ |  |  |  |  |
| **127** | Aamir, A, et al. | 2023 | Autonomy; Privacy; | ~ | Safety; | ~ |  |  |  |  |  |  |  |  |
| **128** | Uche-Anya, E, et al. | 2022 | Privacy; | ~ |  |  | Transparency; | ~ |  |  | Bias ; Disparity; Equality; Equity; Fairness; Inclusiveness; Justice; | ✔ |  |  |
| **129** | Cao, JS, et al. | 2021 |  |  |  |  | Explainability; Interpretability; Transparency; | ~ | Liability; Responsibility; | ✔ | Bias; | ~ |  |  |
| **130** | Matheny, ME, et al. | 2020 | Privacy; | ~ | Safety; | ~ | Transparency; | ✔ | Liability; | ~ | Bias ; Disparity; Equity; Inclusiveness; | ✔ |  |  |
| **131** | Habli, I, et al. | 2020 |  |  | Harm; Safety; | ✔ | Transparency; Understandability; | ~ | Accountability; Liability; Responsibility; | ✔ | Bias ; Equality; | ~ |  |  |
| **132** | Guan, J | 2019 | Autonomy; Informed consent; Privacy; | ~ | Beneficence; Efficacy; Harm; Non-maleficence; Safety; Well-being; | ~ | Explicability; Transparency; | ~ | Liability; | ~ | Discrimination; Equality; Justice; Fairness; | ~ | Environment; | ~ |
| **133** | Schönberger, D | 2019 | Autonomy; Informed consent; Privacy; | ✔ | Efficacy; Harm; Safety; Well-being; | ~ | Explainability; Intelligibility; Interpretability; Transparency; Understandability; | ✔ | Accountability; Liability; Responsibility; | ✔ | Bias ; Discrimination; Disparity; Equality; Equity; Fairness; Inclusiveness; Justice; | ✔ |  |  |
| **134** | Sunarti, S, et al. | 2021 | Autonomy; Confidentiality; Privacy; | ~ | Efficacy; Safety; Well-being; | ~ | Explainability; Interpretability; Transparency; | ~ | Accountability; | ~ | Bias ; Discrimination; Equality; Equity; Equality; Equality; Justice; | ✔ | Sustainability; | ~ |
| **135** | Pennestri, F, et al. | 2022 | Autonomy; Informed consent; Privacy; | ✔ | Beneficence; Harm; Non-maleficence; Safety; Well-being; | ✔ |  |  | Liability; Responsibility; | ~ | Bias ; Discrimination; Disparity; Equality; Equity; Fairness; Justice; | ✔ | Sustainability; | ~ |
| **136** | Badnjevic, A, et al. | 2021 | Privacy; | ~ | Safety; | ✔ |  |  | Accountability; Liability; | ~ | Bias ; Equity; Fairness; | ~ |  |  |
| **138** | Jotterand, F, et al. | 2021 | Autonomy; Privacy; | ~ | Beneficence; Non-maleficence; Safety; Well-being; | ~ |  |  |  | ~ | Bias ; Discrimination; Equality; Equity; Justice; | ~ |  |  |
| **140** | Niel, O, et al. | 2019 | Confidentiality; Privacy; | ✔ | Safety; | ~ | Interpretability; Understandability; | ✔ | Liability; | ~ |  |  |  |  |
| **141** | Xie, G, et al. | 2020 | Privacy; | ~ | Efficacy; Harm; Safety; Well-being; | ✔ | Explainability; Transparency; | ~ | Responsibility; | ~ |  |  |  |  |
| **142** | O'Connor, S, et al. | 2023 | Privacy; | ~ | Efficacy; Safety; Well-being; | ~ | Transparency; | ~ | Accountability; | ~ | Bias ; Equity; Justice; | ~ |  |  |
| **143** | Shreve, JT, et al. | 2022 |  |  | Harm; | ~ | Interpretability; Transparency; | ✔ | Responsibility; | ~ | Bias ; Disparity; Equality; Equity; Fairness; Inclusiveness; | ✔ |  |  |
| **144** | Filler, G, et al. | 2023 | Autonomy; Confidentiality; Privacy; | ✔ | Beneficence; Harm; Non-maleficence; Safety; | ~ | Transparency; | ~ | Liability; Responsibility; | ✔ | Bias ; Equality; Equity; Inclusiveness; Justice; | ~ |  |  |
| **145** | Jarvis, T, et al. | 2020 | Autonomy; Confidentiality; Informed consent; | ~ |  |  |  |  |  |  | Bias; | ~ |  |  |
| **146** | Brady, AP, et al. | 2020 | Privacy; | ✔ | Harm; Safety; | ~ | Explainability; Explicability; Interpretability; Transparency; Understandability; | ✔ | Accountability; Liability; Responsibility; | ✔ | Bias ; Equality; Equity; Fairness; | ✔ |  |  |
| **147** | Mazurowski, MA | 2020 |  |  | Well-being; | ~ | Transparency; | ~ | Responsibility; | ~ |  |  |  |  |
| **148** | Francolini, G, et al. | 2020 |  |  |  |  | Transparency; Understandability; | ✔ | Responsibility; | ~ |  |  |  |  |
| **149** | Rolfes, V, et al. | 2023 | Autonomy; Informed consent; | ✔ | Efficacy; Harm; | ✔ | Explainability; Transparency; Understandability; | ✔ | Liability; Responsibility; | ~ | Discrimination; Fairness; Equality; Justice; | ✔ |  |  |
| **151** | Momtazmanesh, S, et al. | 2022 |  |  | Harm; | ~ | Interpretability; Transparency; Understandability; | ~ |  |  | Bias ; Discrimination; Equity; | ✔ |  |  |
| **152** | Goldstein, CA, et al. | 2020 | Privacy; | ~ | Safety; Well-being; | ~ | Transparency; Understandability; | ~ | Liability; Responsibility; | ~ | Bias ; Disparity; Equity; | ✔ |  |  |
| **153** | Kinoshita, T, et al. | 2023 |  |  | Safety; | ~ |  |  |  |  |  |  |  |  |
| **154** | De Simone, B, et al. | 2022 | Autonomy; Privacy; | ~ | Harm; Safety; | ~ | Explainability; Transparency; | ~ | Accountability; Human supervision; Liability; Responsibility; | ~ | Equality; Equity; Inclusiveness; | ~ |  |  |
| **155** | Rompianesi, G, et al. | 2022 | Autonomy; Confidentiality; Privacy; | ~ | Safety; | ~ | Explainability; Intelligibility; Transparency; | ~ |  |  | Bias; | ~ |  |  |
| **156** | Bellini, V, et al. | 2021 | Informed consent; Privacy; | ~ | Efficacy; Safety; | ~ | Transparency; | ~ |  |  | Bias ; Equality; | ~ |  |  |
| **157** | Etienne, H, et al. | 2020 | Informed consent; Privacy; | ✔ | Efficacy; Public interest; Safety; | ~ | Explainability; Interpretability; Transparency; Understandability; | ~ | Liability; Responsibility; | ✔ | Bias; | ~ |  |  |
| **159** | Wilhelm, D, et al. | 2020 | Privacy; | ~ | Efficacy; Safety; | ✔ | Explainability; Interpretability; Transparency; Understandability; | ✔ | Liability; Responsibility; | ~ | Bias ; Fairness; Inclusiveness; | ✔ |  |  |
| **160** | Morrow, E, et al. | 2022 | Privacy; | ~ | Efficacy; Harm; Safety; | ~ |  |  | Liability; | ~ | Bias ; Discrimination; Disparity; Equality; Equity; Fairness; | ~ | Responsiveness;  Sustainability; | ~ |
| **161** | Ledzinski, L, et al. | 2023 | Privacy; | ✔ | Safety; | ✔ | Explainability; Interpretability; Transparency; Understandability; | ✔ | Responsibility; | ~ | Bias ; Discrimination; Equity; | ~ |  |  |
| **162** | Gedefaw, L, et al. | 2023 | Autonomy; Confidentiality; Privacy; | ✔ |  |  | Explainability; Interpretability; Transparency; Understandability; | ✔ |  |  | Bias ; Disparity; | ~ |  |  |
| **163** | Guo, Y, et al. | 2024 | Privacy; | ~ | Efficacy; Safety; | ~ | Explainability; Transparency; Understandability; | ~ | Responsibility; | ~ | Bias ; Fairness; | ~ |  |  |
| **164** | Monlezun, DJ, et al. | 2022 | Privacy; | ~ | Safety; Well-being; | ~ | Transparency; | ~ |  |  | Discrimination; Disparity; Equity; Fairness; Equality; Justice; | ✔ |  |  |
| **165** | Hameed, BS, et al. | 2022 | Confidentiality; | ~ | Safety; | ~ | Explainability; Understandability; | ~ | Accountability; Responsibility; | ~ |  |  |  |  |
| **166** | Skuban-Eiseler, T, et al. | 2023 | Autonomy; | ✔ | Beneficence; Harm; Non-maleficence; Well-being; | ✔ | Explicability; Transparency; Understandability; | ~ | Accountability; Responsibility; | ~ | Bias ; Equality; Equity; Justice; | ✔ |  |  |
| **167** | Challen, R, et al. | 2019 |  |  | Safety; | ✔ | Explainability; Interpretability; | ✔ |  |  | Bias ; Equality; | ~ |  |  |
| **168** | Day, TG, et al. | 2021 |  |  | Harm; | ✔ | Explainability; | ~ |  |  | Bias; | ~ |  |  |
| **169** | Theodosiou, AA, et al. | 2023 | Privacy; | ~ | Safety; | ~ | Interpretability; Transparency; | ~ | Accountability; | ~ | Bias ; Equity; Equality; | ✔ |  |  |
| **170** | Jalal, S, et al. | 2019 | Autonomy; Privacy; | ~ | Beneficence; Safety; | ~ | Transparency; | ~ |  |  |  |  |  |  |
| **171** | Fayos De Arizon, L, et al. | 2023 | Autonomy; Confidentiality; Privacy; | ✔ | Safety; Well-being; | ~ | Explainability; Interpretability; Transparency; Understandability; | ✔ | Responsibility; | ~ | Bias ; Discrimination; Fairness; | ✔ | Environment ; Sustainability; | ✔ |
| **173** | Neri, E, et al. | 2020 | Autonomy; Confidentiality; Informed consent; Privacy; | ~ | Harm; Safety; Well-being; | ~ | Explicability; Interpretability; Transparency; Understandability; | ✔ | Accountability; Liability; Responsibility; | ✔ | Discrimination; Equity; Fairness; | ~ | Environment; | ~ |
| **174** | Masoumian Hosseini, M, et al. | 2023 | Autonomy; Confidentiality; Informed consent; Privacy; | ~ | Harm; Safety; Well-being; | ~ | Explainability; Transparency; | ✔ | Liability; | ~ | Bias ; Discrimination; Equity; Fairness; | ✔ |  |  |
| **176** | Haley, LC, et al. | 2023 | Autonomy; Informed consent; | ✔ |  |  | Understandability; | ~ |  |  | Equality; Equity; Justice; | ✔ |  |  |
| **177** | Stewart, J, et al. | 2023 | Autonomy; Confidentiality; Privacy; | ✔ | Safety; | ✔ | Explainability; Transparency; | ✔ | Accountability; Liability; Responsibility; | ✔ | Bias ; Discrimination; Equality; Equity; | ✔ |  |  |
| **178** | Clement, J, et al. | 2021 |  |  | Harm; | ~ | Explainability; Interpretability; | ✔ |  |  | Bias; | ~ |  |  |
| **179** | Currie, G, et al. | 2022 | Autonomy; Informed consent; Privacy; | ~ | Safety; | ~ | Transparency; | ~ | Liability; Responsibility; | ✔ | Bias; | ~ |  |  |
| **184** | Eppler, M, et al. | 2023 | Confidentiality; Human oversight; Privacy; | ~ | Harm; Safety; | ~ | Explainability; Understandability; | ~ | Responsibility; | ~ | Bias ; Disparity; | ~ |  |  |
| **185** | Ahmad, OF, et al. | 2020 | Autonomy; Confidentiality; Informed consent; Privacy; | ✔ | Efficacy; Harm; Safety; | ✔ | Explainability; Interpretability; Transparency; | ✔ | Accountability; Liability; Responsibility; | ✔ | Bias ; Discrimination; | ✔ |  |  |
| **186** | Nair, AV, et al. | 2022 | Confidentiality; Informed consent; Privacy; | ✔ | Safety; | ~ | Transparency; | ~ | Accountability; Liability; | ~ | Bias ; Equality; Equity; Fairness; | ✔ |  |  |
| **187** | Veritti, D, et al. | 2023 | Confidentiality; Privacy; | ✔ | Harm; Safety; | ~ | Explainability; Interpretability; Transparency; Understandability; | ✔ | Accountability; Liability; Responsibility; | ✔ | Bias ; Equality; Fairness; | ✔ |  |  |
| **188** | McCoy, LG, et al. | 2022 | Autonomy; Human oversight; Informed consent; | ✔ | Beneficence; Harm; Non-maleficence; | ~ | Explainability; Explicability; Transparency; Understandability; | ✔ | Liability; | ~ | Bias ; Fairness; Justice; | ~ |  |  |
| **189** | Parker, JC | 2023 | Autonomy; | ~ |  |  | Explainability; Interpretability; Understandability; | ✔ |  |  |  |  |  |  |
| **190** | Wojcik, S, et al. | 2023 | Confidentiality; Privacy; | ~ | Safety; | ~ |  |  | Liability; Responsibility; | ~ |  |  |  |  |
| **191** | Nittas, V, et al. | 2023 | Informed consent; Privacy; | ✔ | Efficacy; Harm; Safety; | ✔ | Explainability; Interpretability; Transparency; Understandability; | ✔ | Accountability; Liability; Responsibility; | ✔ | Bias ; Equality; Equity; Fairness; | ✔ |  |  |
| **193** | Reilly-Shah, VNO, et al. | 2020 | Autonomy; Informed consent; | ✔ | Harm; | ~ | Interpretability; Transparency; Understandability; | ~ | Liability; | ~ | Bias ; Discrimination; Disparity; Fairness; | ✔ |  |  |
| **194** | Delgado, J, et al. | 2022 | Informed consent; Privacy; | ~ | Harm; | ~ | Transparency; Understandability; | ~ |  |  | Bias ; Disparity; Equality; Fairness; | ✔ |  |  |
| **195** | Asilian Bidgoli, A, et al. | 2022 |  |  |  |  | Explainability; Transparency; | ~ |  |  | Bias ; Discrimination; | ✔ |  |  |
| **197** | Kellmeyer, P | 2021 | Autonomy; Privacy; | ✔ | Harm; Safety; Well-being; | ✔ | Transparency; | ~ | Accountability; | ~ | Bias ; Inclusiveness; | ✔ |  |  |
| **198** | Lazcoz Moratinos, G, et al. | 2020 | Privacy; | ~ | Safety; | ~ |  |  |  |  | Bias ; Discrimination; | ~ |  |  |
| **200** | van de Leur, RR, et al. | 2020 | Confidentiality; Privacy; | ✔ | Safety; | ~ | Interpretability; Transparency; | ✔ | Accountability; Liability; Responsibility; | ~ | Bias ; Discrimination; | ~ |  |  |
| **201** | Manrique de Lara, A, et al. | 2020 | Autonomy; Confidentiality; Informed consent; Privacy; | ✔ | Harm; | ~ | Explainability; Transparency; Understandability; | ✔ | Accountability; Responsibility; | ✔ | Bias ; Discrimination; Disparity; Equality; Equity; Fairness; Justice; | ✔ |  |  |
| **202** | Ngiam, KY, et al. | 2019 | Privacy; | ~ | Efficacy; Safety; | ✔ | Explainability; Interpretability; Transparency; Understandability; | ~ | Liability; | ✔ | Discrimination; Equity; | ~ |  |  |
| **205** | Parker, JC | 2022 | Autonomy; Informed consent; Privacy; | ~ |  |  | Explainability; Transparency; Understandability; | ✔ |  |  | Bias; | ~ |  |  |
| **208** | Stogiannos, N, et al. | 2023 | Autonomy; Confidentiality; Informed consent; Privacy; | ~ | Harm; Safety; Well-being; | ✔ | Explainability; Interpretability; Transparency; Understandability; | ✔ | Accountability; Liability; | ~ | Bias ; Discrimination; Equity; Fairness; Inclusiveness; | ✔ | Continuous monitoring; | ✔ |
| **210** | Aliyeva, A | 2023 | Privacy; | ~ | Well-being; | ~ | Transparency; | ~ | Accountability; | ~ | Equity; | ~ |  |  |
| **211** | Post, B, et al. | 2022 | Autonomy; Informed consent; Privacy; | ~ | Beneficence; Harm; Non-maleficence; Safety; Well-being; | ~ | Transparency; | ~ |  |  | Bias ; Fairness; Justice; | ~ |  |  |
| **212** | Brown, C, et al. | 2023 | Human oversight; Informed consent; Privacy; | ~ | Harm; Safety; Well-being; | ✔ | Interpretability; Transparency; Understandability; | ~ | Accountability; Liability; Responsibility; | ✔ | Bias; | ~ |  |  |
| **213** | Martinez-Martin, N, et al. | 2022 |  |  |  |  |  |  |  |  | Bias ; Disparity; Fairness; Equality; Equity; | ✔ |  |  |
| **214** | Ali, R, et al. | 2024 | Informed consent; | ✔ | Harm; Safety; | ~ |  |  |  |  | Disparity; Inclusiveness; | ~ |  |  |
| **215** | Doo, FX, et al. | 2023 | Informed consent; Privacy; | ~ | Harm; | ~ | Interpretability; | ~ |  |  | Bias ; Discrimination; Equality; Equity; Fairness; Inclusiveness; Justice; | ✔ |  |  |
| **217** | Menegatti, D, et al. | 2023 | Confidentiality; Privacy; | ✔ |  |  | Explainability; Interpretability; Understandability; | ✔ |  |  |  |  |  |  |
| **218** | Ho, CW, et al. | 2021 | Autonomy; Confidentiality; Privacy; | ~ | Beneficence; Efficacy; Harm; Non-maleficence; Safety; Well-being; | ~ | Explainability; Transparency; | ~ | Accountability; Liability; Redress; Responsibility; | ~ | Bias ; Discrimination; Equality; Equity; Fairness; Justice; | ~ | Continuous monitoring ; Environment ; Responsiveness; Sustainability; | ✔ |
| **219** | Chen, IY, et al. | 2019 |  |  |  |  |  |  |  |  | Bias ; Disparity; Fairness; | ✔ |  |  |
| **220** | Liu, HY, et al. | 2023 | Confidentiality; Privacy; | ~ | Harm; | ~ |  |  | Accountability; Human oversight; | ~ |  |  |  |  |
| **221** | Tabudlo, J, et al. | 2022 |  |  | Harm; Safety; | ~ |  |  | Accountability; Liability; Responsibility; | ✔ |  |  |  |  |
| **222** | Pachamanova, D, et al. | 2022 |  |  |  |  | Interpretability; | ~ |  |  | Bias ; Equity; | ~ |  |  |
| **223** | Kotter, E, et al. | 2021 | Privacy; | ~ | Harm; Well-being; | ~ | Transparency; Understandability; | ~ | Accountability; Responsibility; | ~ | Bias; | ~ |  |  |
| **224** | Lopez, DM, et al. | 2022 | Confidentiality; Informed consent; Privacy; | ~ | Efficacy; Safety; | ~ | Explainability; Transparency; | ✔ | Accountability; Liability; | ~ | Bias ; Disparity; Equality; Equity; | ✔ | Climate; Environment ; Sustainability; | ~ |
| **225** | Mazaheri, S, et al. | 2021 |  |  |  |  | Explainability; Transparency; | ~ |  |  | Bias; | ~ | Continuous monitoring; | ~ |
| **226** | Tranter-Entwistle, I, et al. | 2021 | Informed consent; Privacy; | ~ |  |  | Explainability; Transparency; Understandability; | ~ |  |  | Bias ; Discrimination; | ~ |  |  |
| **227** | Kleebayoon, A, et al. | 2023 | Human oversight; | ~ |  |  | Transparency; | ~ |  |  | Bias; | ~ |  |  |
| **228** | Xu, L, et al. | 2021 | Autonomy; Confidentiality; Informed consent; Privacy; | ✔ | Efficacy; Harm; Safety; Well-being; | ✔ | Explainability; Transparency; Understandability; | ✔ | Liability; Responsibility; | ~ | Bias ; Discrimination; Disparity; Fairness; | ✔ |  |  |
| **230** | Doyal, AS, et al. | 2023 | Privacy; | ✔ | Harm; Well-being; | ✔ | Transparency; Understandability; | ✔ | Accountability; | ~ | Bias ; Discrimination; | ✔ |  |  |
| **231** | Ferdush, J, et al. | 2023 | Confidentiality; Human oversight; Informed consent; Privacy; | ✔ | Efficacy; Safety; | ~ | Explainability; Interpretability; Transparency; Understandability; | ✔ |  |  | Bias ; Equity; | ✔ |  |  |
| **232** | Kuang, YR, et al. | 2023 | Confidentiality; Privacy; | ~ | Safety; | ✔ | Transparency; | ✔ | Accountability; Liability; Responsibility; | ~ | Bias; | ~ |  |  |
| **233** | Jeyaraman, M, et al. | 2023 | Autonomy; Privacy; | ~ | Beneficence; Harm; Non-maleficence; Safety; | ~ | Transparency; Understandability; | ~ | Accountability; | ~ | Bias ; Discrimination; Fairness; | ~ | Environment ; Sustainability; | ~ |
| **234** | Li, W, et al. | 2023 | Confidentiality; Privacy; | ✔ | Harm; Safety; | ✔ |  |  | Liability; | ~ | Equity; Bias; | ✔ |  |  |
| **235** | Sarma, G, et al. | 2023 |  |  | Well-being; | ~ | Transparency; | ~ |  |  | Bias; | ~ |  |  |
| **236** | Dave, T, et al. | 2023 | Human oversight; Privacy; | ~ | Safety; Well-being; | ~ | Transparency; | ~ | Accountability; Responsibility; | ✔ | Bias ; Discrimination; Disparity; Fairness; | ✔ | Environment ; Sustainability; | ~ |
| **237** | Giorgino, R, et al. | 2023 | Confidentiality; Privacy; | ~ | Harm; Safety; | ~ | Transparency; Understandability; | ~ | Accountability; Responsibility; | ~ | Bias; | ✔ |  |  |
| **238** | Sharma, SC, et al. | 2023 | Informed consent; Privacy; | ✔ |  |  | Understandability; | ~ | Responsibility; | ~ |  |  |  |  |
| **239** | Srivastav, S, et al. | 2023 | Confidentiality; Human oversight; Informed consent; Privacy; | ✔ | Harm; Safety; | ✔ | Explainability; Interpretability; Transparency; Understandability; | ✔ | Accountability; | ~ | Bias ; Discrimination; Disparity; Equity; Fairness; | ✔ |  |  |
| **241** | Sallam, M | 2023 | Privacy; | ~ | Harm; Safety; | ~ | Interpretability; Transparency; | ~ | Accountability; Responsibility; | ~ | Bias ; Discrimination; Equity; Fairness; | ~ | Environment ; Climate; | ~ |
| **242** | Temsah, MH, et al. | 2023 | Privacy; | ~ |  |  | Transparency; | ~ |  |  | Bias ; Equity; | ~ |  |  |
| **243** | Beculic, H, et al. | 2024 | Privacy; | ~ |  |  | Transparency; | ~ | Accountability; Responsibility; | ~ | Bias ; Disparity; Equity; | ~ |  |  |
| **244** | Kaneda, Y, et al. | 2023 | Confidentiality; Human oversight; | ~ | Efficacy; | ✔ |  |  | Responsibility; | ~ |  |  |  |  |
| **245** | Smith, H | 2021 |  |  | Harm; Safety; | ~ | Explainability; Transparency; Understandability; | ✔ | Accountability; Liability; Responsibility; | ✔ |  |  |  |  |
| **246** | Watson, DS, et al. | 2019 | Informed consent; Privacy; | ~ | Harm; | ✔ | Explainability; Intelligibility; Interpretability; Transparency; Understandability; | ✔ | Liability; Responsibility; | ~ |  |  |  |  |
| **248** | Birch, J, et al. | 2022 | Autonomy; | ✔ |  |  | Transparency; | ~ |  |  |  |  |  |  |
| **250** | McCradden, MD, et al. | 2020 | Human oversight; Privacy; | ✔ | Beneficence; Non-maleficence; | ~ |  |  | Accountability; | ~ | Justice; | ~ |  |  |
| **252** | Novak, LL, et al. | 2023 |  |  | Harm; Safety; | ✔ | Transparency; | ~ |  |  | Bias ; Fairness; Inclusiveness; Equality; Equity; Justice; | ✔ | Continuous improvement; | ✔ |
| **253** | Scott, I, et al. | 2021 | Autonomy; Confidentiality; Privacy; | ~ | Efficacy; Harm; Safety; | ✔ | Explainability; Intelligibility; Interpretability; Transparency; Understandability; | ✔ | Liability; Responsibility; | ~ | Bias ; Discrimination; Equity; Fairness; Inclusiveness; | ✔ |  |  |
| **255** | Smith, H, et al. | 2023 | Autonomy; Confidentiality; Human oversight; Privacy; | ~ | Beneficence; Harm; Non-maleficence; Safety; | ✔ | Explainability; Transparency; Understandability; | ✔ | Accountability; Liability; Responsibility; | ✔ | Bias ; Equality; Fairness; Justice; | ~ |  |  |
| **256** | Lei, II, et al. | 2023 | Confidentiality; | ~ | Safety; | ~ | Interpretability; Transparency; Understandability; | ✔ |  |  | Bias; | ✔ |  |  |
| **257** | Felder, RM | 2021 | Autonomy; Informed consent; | ~ | Beneficence; Efficacy; Harm; | ~ | Explainability; Interpretability; Transparency; Understandability; | ✔ | Accountability; Liability; Redress; Responsibility; | ✔ |  |  |  |  |
| **258** | Srinivasan, ES, et al. | 2022 | Informed consent; | ✔ | Harm; | ~ | Explainability; | ~ | Liability; Responsibility; | ✔ |  |  |  |  |
| **259** | Russell, RG, et al. | 2023 |  |  | Harm; Safety; | ✔ | Interpretability; Transparency; Understandability; | ~ | Human oversight; Responsibility; | *✔* | Bias ; Disparity; Equity; Fairness; Equality; Justice; | ✔ |  |  |
| **260** | Ferreira, AL, et al. | 2023 | Autonomy; Informed consent; | ✔ | Harm; Safety; | ~ |  |  | Accountability; Human supervision; Liability; Responsibility; | ✔ | Bias; | ✔ |  |  |
| **261** | Singhal, M, et al. | 2023 | Privacy; | ~ | Safety; | ✔ | Understandability; | ~ | Accountability; Liability; Responsibility; | ~ | Bias; | ~ |  |  |
| **262** | Lehoux, P, et al. | 2023 | Privacy; | ✔ | Efficacy; Harm; Safety; | ✔ | Explainability; Transparency; Understandability; | ~ | Accountability; Human oversight; Redress; Responsibility; | ✔ | Bias ; Discrimination; Disparity; Equity; Fairness; Equality; | ✔ | Carbon; Climate; Ecosystem; Energy efficien (-t; Environment; Responsiveness; Sustainability; | ✔ |
| **263** | Berbis, MA, et al. | 2023 | Human oversight; Informed consent; Privacy; | ✔ | Harm; Safety; | ~ | Explainability; | ✔ | Accountability; Liability; Responsibility; | ✔ | Bias ; Disparity; | ✔ |  |  |
| **264** | Verghese, G, et al. | 2023 | Confidentiality; Privacy; | ✔ | Efficacy; Safety; | ~ | Explainability; Interpretability; | ✔ | Accountability; Liability; | ~ | Bias ; Equity; Fairness; | ✔ | Continuous monitoring; | ✔ |
| **265** | McDougall, RJ | 2019 | Autonomy; | ✔ |  |  |  |  | Responsibility; | ~ |  |  | Responsiveness; | ~ |
| **266** | Thaxton, C, et al. | 2023 | Autonomy; | ~ | Beneficence; Efficacy; Harm; Non-maleficence; Safety; | ✔ | Explainability; Transparency; | ✔ |  |  | Bias ; Disparity; Equity; Fairness; Justice; | ✔ |  |  |
| **267** | Starke, G, et al. | 2021 | Autonomy; Informed consent; Privacy; | ✔ | Beneficence; Harm; Non-maleficence; Safety; | ✔ | Explicability; Interpretability; Understandability; | ~ | Accountability; Liability; Responsibility; | ~ | Bias ; Equality; Equity; Fairness; Justice; | ✔ |  |  |
| **268** | Rose, C, et al. | 2023 | Privacy; | ✔ | Efficacy; Safety; | ~ | Transparency; | ~ | Accountability; | ~ | Bias ; Disparity; Equity; Fairness; Inclusiveness; | ✔ | Sustainability; | ~ |
| **269** | Allen, JW, et al. | 2023 | Autonomy; Confidentiality; Informed consent; Privacy; | ✔ | Harm; Non-maleficence; Safety; Well-being; | ~ | Explainability; | ~ | Accountability; Liability; Responsibility; | ✔ | Bias ; Fairness; | ~ |  |  |
| **270** | Abramoff, MD, et al. | 2023 | Autonomy; Human oversight; | ~ | Beneficence; Efficacy; Harm; Safety; | ~ | Explainability; Transparency; | ~ |  |  | Bias ; Disparity; Equality; Equity; Inclusiveness; Justice; | ✔ |  |  |
| **271** | Marti-Bonmati, L, et al. | 2022 | Human oversight; Informed consent; Privacy; | ✔ | Harm; Safety; | ~ | Explainability; | ~ | Accountability; Responsibility; | ✔ | Bias ; Disparity; Equity; Fairness; | ✔ |  |  |
| **273** | Bishara, A, et al. | 2022 | Autonomy; Informed consent; Privacy; | ✔ | Beneficence; Harm; Non-maleficence; Safety; Well-being; | ✔ | Explainability; Interpretability; Transparency; Understandability; | ✔ | Accountability; Liability; Responsibility; | ✔ | Bias ; Disparity; Equity; Fairness; Justice; | ✔ |  |  |
| **274** | Beltrami, EJ, et al. | 2023 |  |  | Beneficence; Non-maleficence; | ✔ |  |  |  |  | Bias ; Justice; | ✔ |  |  |
| **276** | Liu, TYA, et al. | 2020 | Confidentiality; | ✔ |  |  | Explainability; Interpretability; Understandability; | ✔ | Responsibility; | ~ | Bias; | ✔ |  |  |
| **279** | Fan, ZY, et al. | 2023 |  |  |  |  | Interpretability; | ~ | Responsibility; | ~ | Bias ; Fairness; | ~ |  |  |
| **280** | Buruk, B, et al. | 2020 | Autonomy; Confidentiality; Informed consent; Privacy; | ✔ | Harm; Safety; Well-being; | ✔ | Explicability; Transparency; Understandability; | ✔ | Accountability; Responsibility; | ~ | Bias ; Discrimination; Equality; Equity; Fairness; Inclusiveness; Justice; | ✔ | Environment ; Sustainability; | ~ |
| **282** | Sharma, S, et al. | 2023 | Autonomy; Confidentiality; Informed consent; Privacy; | ✔ | Efficacy; Harm; Safety; Well-being; | ✔ | Understandability; | ✔ |  |  | Bias ; Discrimination; Disparity; Equity; | ✔ |  |  |
| **283** | Potocnik, J, et al. | 2023 | Privacy; | ✔ | Safety; | ✔ | Explainability; | ~ |  |  | Bias ; Equity; | ✔ |  |  |
| **284** | Fischer, UM, et al. | 2021 | Privacy; | ~ | Safety; | ✔ | Explainability; Interpretability; | ~ |  |  | Bias ; Disparity; | ~ |  |  |
| **285** | Antoniadi, AM, et al. | 2021 | Privacy; | ~ | Harm; Safety; | ~ | Explainability; Interpretability; Transparency; Understandability; | ✔ | Accountability; | ~ | Bias ; Discrimination; Fairness; Equality; Equity; | ✔ |  |  |
| **287** | Saw, SN, et al. | 2022 | Autonomy; Confidentiality; Privacy; | ✔ | Harm; Safety; Well-being; | ~ | Explainability; Transparency; Understandability; | ✔ | Accountability; Liability; Responsibility; | ✔ | Bias ; Discrimination; Equity; Fairness; | ✔ | Environment ; Sustainability; | ~ |
| **288** | Gauss, T, et al. | 2023 | Confidentiality; Human oversight; Privacy; | ~ | Efficacy; Harm; Safety; | ~ | Explainability; Interpretability; Transparency; Understandability; | ~ |  |  | Bias; | ~ |  |  |
| **292** | Cui, C, et al. | 2019 | Informed consent; Privacy; | ✔ | Public interest; | ~ |  |  | Accountability; Responsibility; | ~ | Bias ; Discrimination; Disparity; Fairness; Justice; | ✔ |  |  |
| **293** | Monah, SR, et al. | 2022 | Privacy; | ✔ | Beneficence; Safety; Well-being; | ~ | Transparency; | ~ | Accountability; Responsibility; | ~ | Bias ; Discrimination; Fairness; Justice; | ~ |  |  |
| **294** | Martínez-García, M, et al. | 2022 | Confidentiality; Privacy; | ✔ | Efficacy; Safety; | ~ | Intelligibility; Interpretability; Transparency; | ~ |  |  | Bias ; Fairness; | ~ |  |  |
| **295** | Luo, MH, et al. | 2022 |  |  |  |  |  |  |  |  | Bias ; Equality; Equity; Fairness; | ✔ | Sustainability; | ~ |
| **297** | Shiri, I, et al. | 2022 | Privacy; | ✔ |  |  |  |  |  |  |  |  |  |  |
| **298** | Mangold, P, et al. | 2020 | Privacy; | ✔ |  |  |  |  |  |  |  |  |  |  |
| **299** | Loftus, TJ, et al. | 2020 |  |  | Harm; | ~ | Interpretability; Transparency; Understandability; | ~ | Liability; | ~ |  |  |  |  |
| **301** | Duran, HT, et al. | 2023 | Autonomy; | ~ | Safety; | ✔ | Explainability; Interpretability; Transparency; Understandability; | ✔ | Accountability; Liability; | ✔ | Bias ; Equity; Equality; | ~ |  |  |
| **302** | Ho, A | 2019 | Autonomy; Privacy; | ~ | Safety; Well-being; | ✔ | Intelligibility; Transparency; Understandability; | ~ | Accountability; | ~ | Bias ; Discrimination; Disparity; Equity; Fairness; | ✔ |  |  |
| **304** | Khan, AA, et al. | 2021 | Autonomy; | ~ | Beneficence; Harm; | ✔ | Explainability; Transparency; Understandability; | ✔ | Accountability; Liability; | ~ | Bias; | ~ |  |  |
| **305** | McBee, MP, et al. | 2018 | Autonomy; | ~ | Safety; | ~ |  |  | Responsibility; | ✔ |  |  |  |  |
| **306** | Fernandez-Quilez, A, et al. | 2023 | Confidentiality; Informed consent; Privacy; | ✔ | Efficacy; Harm; Safety; | ~ | Explainability; Interpretability; Transparency; Understandability; | ✔ |  |  | Bias ; Discrimination; Disparity; Equality; Equity; Inclusiveness; | ✔ | Continuous monitoring; | ~ |
| **307** | Montagnon, E, et al. | 2020 | Autonomy; Informed consent; Privacy; | ✔ |  |  |  |  | Human supervision; Responsibility; | ~ |  |  | Continuous monitoring; | ✔ |
| **308** | Adams, J | 2023 | Autonomy; Human oversight; Informed consent; Privacy; | ✔ | Beneficence; Harm; Non-maleficence; | ✔ | Explainability; Explicability; Intelligibility; Interpretability; Transparency; Understandability; | ✔ | Accountability; Responsibility; | ✔ | Bias; Equality; Fairness; Justice; | ✔ |  |  |
| **309** | Wadden, JJ | 2021 | Autonomy; Informed consent; | ~ | Efficacy; Harm; | ~ | Explainability; Interpretability; Transparency; Understandability; | ✔ | Liability; | ~ | Discrimination; | ~ |  |  |
| **311** | Thirunavukarasu, AJ, et al. | 2023 | Informed consent; Privacy; | ~ | Safety; | ~ | Explainability; Interpretability; Transparency; Understandability; | ✔ | Responsibility; | ~ | Bias; Fairness; | ~ |  |  |
| **312** | Roy, S, et al. | 2022 | Privacy; | ~ | Beneficence; Efficacy; Safety; | ~ | Explainability; Interpretability; Transparency; Understandability; | ✔ | Accountability; Responsibility; | ~ | Bias; | ~ | Continuous monitoring; | ~ |
| **313** | Duke, SA | 2022 | Privacy; | ~ | Efficacy; Harm; Safety; Well-being; | ✔ | Interpretability; Transparency; Understandability; | ✔ | Accountability; Human oversight; Liability; Responsibility; | ✔ | Bias; Equality; | ~ |  |  |
| **314** | He, M, et al. | 2020 | Informed consent; Privacy; | ~ | Efficacy; | ~ | Transparency; | ~ |  |  |  |  |  |  |
| **315** | Shad, R, et al. | 2021 | Privacy; | ~ | Harm; | ~ | Explainability; Interpretability; Understandability; | ✔ | Liability; | ~ | Bias; Discrimination; Disparity; Equity; Fairness; | ✔ |  |  |
| **316** | Bajgain, B, et al. | 2023 | Privacy; | ~ | Harm; Safety; | ~ | Transparency; | ~ | Accountability; Liability; | ~ |  |  |  |  |
| **317** | Nichol, AA, et al. | 2023 | Privacy; | ~ | Efficacy; Harm; Safety; Well-being; | ✔ | Explainability; Transparency; Understandability; | ✔ | Responsibility; | ✔ | Bias; Disparity; | ✔ |  |  |
| **319** | Bertsimas, D, et al. | 2019 |  |  |  |  | Interpretability; | ✔ |  |  | Bias; Disparity; Equity; | ✔ |  |  |
| **321** | Ishii, E, et al. | 2023 |  |  |  |  | Interpretability; Transparency; Understandability; | ✔ |  |  |  |  |  |  |
| **322** | Hatherley, J, et al. | 2023 | Confidentiality; Privacy; | ✔ | Harm; Non-maleficence; Safety; Well-being; | ✔ | Interpretability; Understandability; | ~ |  |  | Bias; Equity; Justice; | ✔ | Continuous monitoring; | ~ |
| **323** | Allahqoli, L, et al. | 2023 | Confidentiality; | ~ |  |  | Explainability; Interpretability; | ✔ |  |  | Bias; | ~ |  |  |
| **324** | Bleher, H, et al. | 2022 | Informed consent; | ~ | Harm; Safety; Well-being; | ✔ | Explainability; Explicability; Interpretability; Transparency; Understandability; | ✔ | Accountability; Contestability; Human oversight; Liability; Redress; Responsibility; | ✔ | Bias; Discrimination; Fairness; Justice; | ✔ | Continuous monitoring; Sustainability; | ~ |
| **327** | Mattei, P | 2020 | Human oversight; Privacy; | ~ | Safety; Well-being; | ~ | Transparency; Understandability; | ~ | Accountability; Liability; | ✔ | Equity; | ~ |  |  |
| **329** | Kashani, KB, et al. | 2023 |  |  | Beneficence; Efficacy; Harm; Safety; | ~ |  |  |  |  | Bias; Disparity; Equality; | ~ | Continuous monitoring; | ✔ |
| **333** | Armeni, P, et al. | 2022 | Confidentiality; Informed consent; Privacy; | ✔ | Efficacy; Safety; | ~ | Explainability; Transparency; | ~ | Responsibility; | ~ | Bias; Equity; | ✔ |  |  |
| **335** | Wiens, J, et al. | 2019 | Privacy; | ~ | Efficacy; Harm; Safety; | ✔ | Explainability; Interpretability; Transparency; Understandability; | ✔ | Responsibility; | ~ | Bias; Equality; Fairness; | ✔ | Continuous improvement; Continuous monitoring; | ~ |
| **336** | Mazurowski, MA | 2021 |  |  |  |  | Explainability; Interpretability; Transparency; Understandability; | ✔ |  |  | Discrimination; Fairness; | ~ |  |  |
| **337** | Burnside, M, et al. | 2020 | Confidentiality; Privacy; | ✔ | Efficacy; Harm; Safety; | ✔ | Interpretability; Transparency; | ~ | Responsibility; | ~ | Bias; | ~ |  |  |
| **338** | Binkley, CE, et al. | 2021 | Privacy; | ~ | Beneficence; Harm; Safety; | ✔ | Explainability; Transparency; | ✔ | Responsibility; | ✔ | Bias; Fairness; Justice; | ~ |  |  |
| **339** | Hashemi, AS, et al. | 2023 | Privacy; | ✔ |  |  |  |  |  |  | Bias; | ~ |  |  |
| **340** | Khan, B, et al. | 2023 | Confidentiality; Informed consent; Privacy; | ✔ | Beneficence; Efficacy; Harm; Safety; | ~ | Explainability; Interpretability; Transparency; Understandability; | ✔ | Accountability; Responsibility; | ✔ | Bias; Discrimination; Equality; Fairness; | ✔ |  |  |
| **341** | Parums, DV | 2021 | Confidentiality; | ~ |  |  |  |  |  |  |  |  |  |  |
| **342** | Zhang, RW, et al. | 2023 | Privacy; | ~ | Beneficence; Harm; Safety; | ~ | Interpretability; Transparency; Understandability; | ✔ |  |  | Bias; Disparity; Fairness; | ✔ |  |  |
| **343** | Bunnell, A, et al. | 2023 |  |  | Harm; Safety; | ✔ | Explainability; Interpretability; | ~ | Responsibility; | ✔ | Bias; | ~ |  |  |
| **345** | Wouters, PC, et al. | 2023 |  |  |  |  | Explainability; Interpretability; Understandability; | ✔ |  |  | Bias; | ~ |  |  |
| **346** | Garcelon, N, et al. | 2020 | Privacy; | ✔ |  |  | Understandability; | ~ |  |  | Equity; | ~ |  |  |
| **347** | McLennan, S, et al. | 2022 | Autonomy; Confidentiality; Privacy; | ~ | Beneficence; Harm; Non-maleficence; Safety; | ✔ | Explainability; Explicability; Transparency; Understandability; | ✔ | Accountability; Responsibility; | ✔ | Bias; Equity; Fairness; Justice; | ~ |  |  |
| **348** | Karabacak, M, et al. | 2023 | Privacy; | ✔ | Beneficence; Safety; | ~ | Explainability; Interpretability; Transparency; Understandability; | ✔ |  |  | Bias; Equity; Fairness; Inclusiveness; | ✔ | Continuous monitoring; | ~ |
| **350** | Alaidarous, MA | 2020 | Autonomy; Privacy; | ~ | Efficacy; Safety; | ~ | Interpretability; Transparency; | ~ |  |  | Bias; Discrimination; | ~ |  |  |
| **352** | Shaw, J, et al. | 2022 | Informed consent; | ~ | Harm; Well-being; | ~ |  |  |  |  | Equity; Justice; | ~ |  |  |
| **353** | Bart, NK, et al. | 2023 | Confidentiality; Human oversight; Privacy; | ✔ | Beneficence; Efficacy; Harm; Safety; Well-being; | ~ | Explainability; Transparency; | ~ | Accountability; Contestability; Liability; Responsibility; | ~ | Bias; Discrimination; Fairness; | ~ | Environment; | ~ |
| **354** | Pfohl, SR, et al. | 2021 |  |  |  |  | Transparency; | ~ | Contestability; | ~ | Bias; Discrimination; Disparity; Equality; Equity; Fairness; | ✔ | Continuous monitoring; | ~ |
| **355** | Grote, T, et al. | 2022 | Informed consent; | ~ | Beneficence; Efficacy; Harm; Safety; Well-being; | ~ | Explainability; Interpretability; Transparency; | ~ |  |  | Bias; Disparity; Equality; Equity; Fairness; Justice; | ✔ |  |  |
| **356** | Nellaker, C, et al. | 2019 | Informed consent; Privacy; | ✔ |  |  | Interpretability; Understandability; | ~ | Accountability; Liability; | ~ | Bias; Equity; Fairness; | ~ |  |  |
| **358** | Lisboa, PJG, et al. | 2022 |  |  | Safety; | ~ | Explainability; Interpretability; Transparency; Understandability; | ✔ | Responsibility; | ~ | Bias; | ~ |  |  |
| **359** | Aranovich, TD, et al. | 2023 | Informed consent; Privacy; | ~ | Harm; Safety; | ✔ | Explainability; Explicability; Interpretability; Transparency; Understandability; | ✔ | Accountability; Liability; Responsibility; | ✔ | Bias; Discrimination; | ✔ |  |  |
| **360** | Samaan, JS, et al. | 2023 | Human oversight; Privacy; | ✔ | Safety; | ~ | Transparency; | ~ | Accountability; | ~ | Fairness; | ~ |  |  |
| **361** | Rajkomar, A, et al. | 2018 | Autonomy; Privacy; | ~ | Beneficence; Harm; Non-maleficence; Safety; | ~ | Understandability; | ~ |  |  | Bias; Discrimination; Disparity; Equality; Equity; Fairness; Justice; | ✔ | Continuous monitoring; | ~ |
| **364** | Mudgal, KS, et al. | 2020 | Autonomy; Confidentiality; Human oversight; Informed consent; Privacy; | ✔ | Beneficence; Efficacy; Harm; Safety; | ✔ | Explainability; Explicability; Interpretability; Transparency; Understandability; | ✔ | Accountability; Human supervision; Liability; Responsibility; | ✔ | Bias; Discrimination; Equity; Fairness; Inclusiveness; | ✔ | Sustainability; | ~ |
| **365** | Currie, G, et al. | 2021 | Autonomy; Confidentiality; Privacy; | ✔ | Beneficence; Harm; Non-maleficence; Safety; Well-being; | ~ | Explainability; Explicability; Intelligibility; Transparency; Understandability; | ✔ | Accountability; Responsibility; | ~ | Bias; Equality; Equity; Fairness; Inclusiveness; Justice; | ✔ |  |  |
| **368** | Redrup Hill, E, et al. | 2023 | Autonomy; Human oversight; Informed consent; | ✔ | Beneficence; Efficacy; Harm; Non-maleficence; Safety; | ✔ | Explainability; Interpretability; Transparency; Understandability; | ✔ | Accountability; Human supervision; Liability; Redress; Responsibility; | ✔ | Bias; Discrimination; Equality; Equity; Fairness; | ✔ |  |  |
| **372** | Boch, S, et al. | 2022 | Privacy; | ~ | Beneficence; | ~ | Explainability; Transparency; | ✔ | Accountability; | ~ | Bias; Equity; Fairness; Inclusiveness; Justice; | ✔ |  |  |
| **375** | McKay, F, et al. | 2022 | Autonomy; Confidentiality; Informed consent; Privacy; | ✔ | Harm; Safety; | ~ | Explainability; Explicability; Interpretability; Transparency; | ~ | Accountability; | ~ | Bias; Equity; Fairness; | ✔ | Continuous monitoring; | ~ |
| **377** | Prabhu, SP | 2019 | Informed consent; Privacy; | ✔ |  |  | Understandability; | ~ | Liability; Responsibility; | ~ |  |  |  |  |
| **378** | McCradden, MD, et al. | 2020 | Autonomy; Confidentiality; Informed consent; Privacy; | ✔ | Harm; Safety; | ~ | Transparency; | ~ | Accountability; Liability; Responsibility; | ✔ | Equality; Fairness; | ~ |  |  |
| **379** | Lareyre, F, et al. | 2024 |  |  | Safety; | ~ | Transparency; | ~ | Accountability; | ~ | Equity; Fairness; | ~ |  |  |
| **380** | Beil, M, et al. | 2019 | Autonomy; Confidentiality; Human oversight; Informed consent; Privacy; | ✔ | Beneficence; Harm; Non-maleficence; Well-being; | ✔ | Explainability; Explicability; Interpretability; Transparency; Understandability; | ✔ | Accountability; Responsibility; | ~ | Bias; Discrimination; Fairness; Justice; | ✔ | Continuous monitoring; | ~ |
| **381** | Filippi, CG, et al. | 2023 | Autonomy; Informed consent; Privacy; | ✔ | Beneficence; Harm; Non-maleficence; Safety; Well-being; | ~ | Explainability; Explicability; Transparency; Understandability; | ✔ | Accountability; Redress; Responsibility; | ~ | Bias; Disparity; Equality; Equity; Fairness; Justice; | ✔ | Continuous monitoring; | ~ |
| **382** | Rockwell, HD, et al. | 2023 | Informed consent; Privacy; | ✔ | Beneficence; Harm; Safety; | ~ |  |  | Accountability; Liability; Responsibility; | ✔ | Bias; Equality; Equity; | ✔ |  |  |
| **383** | Herington, J, et al. | 2023 | Autonomy; Confidentiality; Human oversight; Informed consent; Privacy; | ✔ | Beneficence; Efficacy; Harm; Non-maleficence; Safety; Well-being; | ✔ | Explainability; Explicability; Interpretability; Transparency; Understandability; | ✔ | Accountability; Responsibility; | ~ | Bias; Discrimination; Disparity; Equality; Equity; Fairness; Justice; | ✔ |  |  |
| **384** | Herington, J, et al. | 2023 | Autonomy; Human oversight; Informed consent; Privacy; | ✔ | Beneficence; Efficacy; Harm; Non-maleficence; Safety; Well-being; | ✔ | Explainability; Explicability; Interpretability; Transparency; Understandability; | ✔ | Accountability; Liability; Responsibility; | ✔ | Bias; Disparity; Equality; Equity; Fairness; Justice; | ✔ | Continuous monitoring; | ~ |
| **385** | Akinci D'Antonoli, T | 2020 | Autonomy; Confidentiality; Informed consent; Privacy; | ✔ | Beneficence; Harm; Non-maleficence; Safety; Well-being; | ✔ | Explainability; Explicability; Intelligibility; Interpretability; Transparency; | ✔ | Accountability; Liability; Responsibility; | ✔ | Bias; Discrimination; Disparity; Equality; Equity; Fairness; Justice; | ✔ | Continuous monitoring; | ~ |
| **386** | Fusar-Poli, P, et al. | 2022 | Autonomy; Confidentiality; Informed consent; Privacy; | ✔ | Beneficence; Harm; Non-maleficence; | ~ | Explainability; Interpretability; Transparency; | ✔ | Accountability; Responsibility; | ~ | Bias; Discrimination; Disparity; Equality; Equity; Fairness; Justice; | ✔ |  |  |
| **387** | MacIntyre, MR, et al. | 2023 | Autonomy; Informed consent; Privacy; | ✔ | Harm; Safety; | ~ | Explainability; Interpretability; Transparency; Understandability; | ~ | Accountability; Responsibility; | ✔ | Bias; Disparity; Fairness; | ✔ |  |  |
| **388** | Petersson, L, et al. | 2023 | Autonomy; Confidentiality; Privacy; | ✔ | Beneficence; Harm; Non-maleficence; Safety; Well-being; | ✔ | Explicability; Interpretability; Transparency; Understandability; | ✔ | Accountability; Liability; Responsibility; | ~ | Bias; Discrimination; Equality; Equity; Fairness; Justice; | ✔ |  |  |
| **389** | Petti, U, et al. | 2023 | Autonomy; Confidentiality; Informed consent; Privacy; | ✔ | Harm; Safety; | ✔ | Explainability; Intelligibility; Interpretability; Transparency; Understandability; | ✔ |  |  | Bias; Discrimination; Equality; Fairness; Inclusiveness; Justice; | ✔ |  |  |
| **390** | Martin, C, et al. | 2022 | Human oversight; Informed consent; Privacy; | ✔ | Harm; Safety; | ~ | Explainability; Interpretability; Transparency; Understandability; | ~ | Responsibility; | ~ | Bias; Disparity; | ✔ | Continuous monitoring; | ~ |
| **392** | Wang, C, et al. | 2023 | Confidentiality; Human oversight; Informed consent; Privacy; | ✔ | Beneficence; Efficacy; Harm; Non-maleficence; Safety; Well-being; | ✔ | Explainability; Interpretability; Transparency; Understandability; | ✔ | Accountability; Liability; Responsibility; | ✔ | Bias; Discrimination; Fairness; Justice; | ✔ | Continuous monitoring; | ~ |
| **393** | Michelson, KN, et al. | 2022 | Autonomy; Informed consent; Privacy; | ✔ | Efficacy; Harm; Safety; | ~ | Explainability; Transparency; | ~ | Liability; Responsibility; | ✔ | Bias; | ✔ |  |  |
| **394** | Prakash, S, et al. | 2022 | Autonomy; Confidentiality; Informed consent; Privacy; | ✔ | Beneficence; Efficacy; Harm; Non-maleficence; Safety; Well-being; | ~ | Explainability; Explicability; Interpretability; Transparency; Understandability; | ~ | Accountability; Human supervision; Liability; Responsibility; | ~ | Bias; Discrimination; Equality; Equity; Fairness; Justice; | ✔ |  |  |
| **396** | Yirmibesoglu Erkal, E, et al. | 2021 | Autonomy; Confidentiality; Human oversight; Informed consent; Privacy; | ✔ | Beneficence; Harm; Non-maleficence; Safety; | ✔ | Transparency; | ~ | Accountability; Responsibility; | ~ | Bias; Disparity; Equality; Fairness; Justice; | ✔ |  |  |
| **397** | Leimanis, A, et al. | 2021 | Autonomy; Human oversight; Informed consent; Privacy; | ~ | Harm; Safety; Well-being; | ~ | Explainability; Interpretability; Transparency; Understandability; | ✔ | Accountability; Human supervision; Liability; Responsibility; | ✔ | Bias; Discrimination; Fairness; Justice; | ~ | Environment; Sustainability; | ~ |
| **399** | Ursin, F, et al. | 2021 | Autonomy; Confidentiality; Human oversight; Privacy; | ✔ | Beneficence; Harm; Non-maleficence; Well-being; | ✔ | Explainability; Explicability; Intelligibility; Interpretability; Transparency; Understandability; | ✔ | Accountability; Liability; Responsibility; | ✔ | Bias; Discrimination; Disparity; Equality; Equity; Fairness; Justice; | ✔ |  |  |
| **400** | Straw, I | 2021 | Autonomy; Confidentiality; Informed consent; | ✔ | Beneficence; Harm; Non-maleficence; Well-being; | ✔ | Transparency; | ~ | Accountability; Liability; Responsibility; | ~ | Bias; Discrimination; Equality; Equity; Fairness; Inclusiveness; Justice; | ✔ |  |  |
| **401** | Loch, AA, et al. | 2022 | Autonomy; Confidentiality; Informed consent; Privacy; | ✔ | Beneficence; Harm; Non-maleficence; | ~ | Explainability; Interpretability; | ✔ | Liability; | ~ | Discrimination; Equity; Fairness; Justice; | ~ |  |  |
| **403** | Matsuzaki, T. | 2018 | Informed consent; Privacy; | ~ | Harm; | ~ | Explainability; Transparency; | ✔ | Liability; Responsibility; | ✔ |  |  |  |  |
| **404** | Karimian, E, et al. | 2022 | Autonomy; Confidentiality; Human oversight; Informed consent; Privacy; | ✔ | Harm; Non-maleficence; Safety; Well-being; | ✔ | Explainability; Explicability; Interpretability; Transparency; | ✔ | Accountability; | ~ | Bias; Discrimination; Equity; Fairness; Inclusiveness; Justice; | ✔ | Environment; Sustainability; | ~ |
| **405** | Ford, E, et al. | 2023 | Autonomy; Confidentiality; Informed consent; Privacy; | ~ | Beneficence; Harm; Non-maleficence; | ~ | Explainability; Intelligibility; Interpretability; Transparency; | ~ | Accountability; Liability; Responsibility; | ~ | Bias; Discrimination; Equality; Equity; Fairness; Justice; | ✔ |  |  |
| **406** | Fritzsche, MC, et al. | 2023 | Autonomy; Confidentiality; Informed consent; Privacy; | ✔ | Safety; | ~ | Explainability; Explicability; Interpretability; Transparency; Understandability; | ✔ | Accountability; Responsibility; | ~ | Bias; Discrimination; Disparity; Equality; Equity; Fairness; Justice; | ✔ |  |  |
| **407** | McCradden, MD, et al. | 2020 | Human oversight; | ~ | Beneficence; Efficacy; Harm; Non-maleficence; Safety; | ~ | Explainability; Interpretability; Transparency; Understandability; | ✔ | Accountability; | ~ | Bias; Discrimination; Equality; Equity; Fairness; Justice; | ✔ |  |  |
| **408** | Chen, IY, et al. | 2021 | Autonomy; Confidentiality; Informed consent; Privacy; | ~ | Beneficence; Harm; Non-maleficence; Safety; | ~ | Interpretability; Understandability; | ~ | Liability; Responsibility; | ~ | Bias; Discrimination; Disparity; Equality; Equity; Fairness; Inclusiveness; Justice; | ✔ | Continuous monitoring; | ~ |
| **410** | Currie, G, et al. | 2020 | Autonomy; Confidentiality; Informed consent; Privacy; | ✔ | Beneficence; Harm; Non-maleficence; Safety; Well-being; | ✔ | Explainability; Explicability; Intelligibility; Interpretability; Transparency; Understandability; | ✔ | Accountability; Liability; Responsibility; | ✔ | Bias; Discrimination; Equity; Fairness; Justice; | ✔ | Sustainability; | ~ |
| **411** | Douglas, DM, et al. | 2022 | Informed consent; | ~ | Harm; Safety; | ~ | Transparency; Understandability; | ~ | Accountability; Liability; Responsibility; | ✔ |  |  | Continuous monitoring; | ~ |
| **414** | Carter, SM, et al. | 2020 | Confidentiality; Human oversight; Informed consent; Privacy; | ✔ | Efficacy; Harm; | ~ | Explainability; Interpretability; Transparency; Understandability; | ✔ | Accountability; Liability; Responsibility; | ✔ | Bias; Discrimination; | ✔ |  |  |
| **415** | Morris, MX, et al. | 2023 | Privacy; | ✔ | Efficacy; Harm; Safety; | ~ | Interpretability; Transparency; | ~ | Accountability; Liability; Responsibility; | ✔ | Bias; Discrimination; Disparity; Equality; | ✔ | Continuous monitoring; | ~ |
| **416** | Cartolovni, A, et al. | 2022 | Autonomy; Informed consent; Privacy; | ~ | Harm; Non-maleficence; Safety; | ✔ | Explainability; Transparency; Understandability; | ✔ | Accountability; Liability; Responsibility; | ✔ | Bias; Discrimination; Disparity; Equality; Fairness; | ~ | Continuous monitoring; | ~ |
| **417** | Morley, J, et al. | 2020 | Autonomy; Human oversight; | ~ | Harm; Safety; | ✔ | Explainability; Understandability; | ~ | Liability; Responsibility; | ~ | Bias; Discrimination; Fairness; | ~ |  |  |
| **418** | Li, F, et al. | 2022 | Autonomy; Confidentiality; Human oversight; Privacy; | ✔ | Beneficence; Harm; Non-maleficence; Safety; Well-being; | ✔ | Explainability; Interpretability; Transparency; Understandability; | ✔ | Accountability; Liability; Responsibility; | ✔ | Bias; Discrimination; Equality; Fairness; Justice; | ✔ | Environment; Sustainability; | ✔ |
| **420** | Zhang, J, et al. | 2023 | Autonomy; Privacy; | ✔ | Beneficence; Efficacy; Harm; Non-maleficence; Safety; Well-being; | ✔ | Explainability; Interpretability; Transparency; Understandability; | ✔ | Accountability; Human supervision; Liability; Responsibility; | ✔ | Bias; Discrimination; Disparity; Equality; Equity; Justice; | ✔ | Continuous monitoring; | ~ |
| **421** | Lareyre, F, et al. | 2023 | Human oversight; Privacy; | ~ | Safety; Well-being; | ~ | Explainability; Interpretability; Transparency; Understandability; | ~ | Accountability; Responsibility; | ~ | Bias; Discrimination; Equity; Fairness; | ~ | Environment; | ~ |
| **422** | Kenny, LM, et al. | 2021 | Autonomy; Informed consent; Privacy; | ✔ | Beneficence; Harm; Non-maleficence; Safety; | ~ | Explainability; Interpretability; Transparency; | ~ | Accountability; Human oversight; Responsibility; | ✔ | Bias; Discrimination; Disparity; Equity; Fairness; Justice; | ✔ | Continuous monitoring; | ~ |
| **423** | Childress, JF, et al. | 2023 | Autonomy; Privacy; | ~ | Beneficence; Harm; Non-maleficence; | ~ | Explicability; Intelligibility; Transparency; | ~ | Accountability; Responsibility; | ~ | Justice; | ~ |  |  |
| **424** | Krupinski, EA | 2020 | Informed consent; Privacy; | ✔ | Beneficence; | ~ |  |  | Human oversight; | ~ |  |  |  |  |
| **425** | Pruski, M | 2023 |  |  | Beneficence; Harm; Non-maleficence; Safety; Well-being; | ✔ | Transparency; | ~ | Accountability; Human oversight; Responsibility; | ~ | Bias; Discrimination; Equality; Fairness; Inclusiveness; Justice; | ✔ | Continuous monitoring; Responsiveness; | ✔ |
| **427** | Kulikowski, CA | 2022 |  |  | Harm; Safety; | ~ | Explainability; | ~ | Accountability; Responsibility; | ~ | Bias; Equity; Fairness; Inclusiveness; | ~ |  |  |
| **428** | Katirai, A | 2023 | Autonomy; Confidentiality; Human oversight; Informed consent; Privacy; | ✔ | Beneficence; Efficacy; Harm; Safety; Well-being; | ✔ | Explainability; Intelligibility; Transparency; Understandability; | ✔ | Accountability; Human supervision; Redress; Responsibility; | ✔ | Bias; Discrimination; Disparity; Equality; Equity; Inclusiveness; | ✔ | Carbon; Climate; Environment; Responsiveness; Sustainability; | ✔ |
| **429** | Liao, SM | 2023 | Autonomy; Informed consent; Privacy; | ✔ | Beneficence; Harm; Non-maleficence; Well-being; | ✔ | Explainability; Interpretability; Understandability; | ✔ |  |  | Bias; Discrimination; Equality; Equity; Fairness; Inclusiveness; Justice; | ✔ |  |  |
| **431** | Morley, J, et al. | 2020 | Autonomy; Confidentiality; Human oversight; Informed consent; Privacy; | ~ | Beneficence; Efficacy; Harm; Non-maleficence; Safety; Well-being; | ✔ | Explainability; Intelligibility; Interpretability; Transparency; Understandability; | ✔ | Accountability; Liability; Redress; Responsibility; | ✔ | Bias; Discrimination; Equality; Equity; Fairness; Justice; | ✔ | Continuous monitoring; | ~ |
| **433** | Chauhan, C, et al. | 2021 | Autonomy; Confidentiality; Informed consent; Privacy; | ✔ | Beneficence; Efficacy; Harm; Safety; | ~ | Transparency; Understandability; | ✔ | Accountability; Human oversight; Human supervision; Liability; Responsibility; | ✔ | Bias; Discrimination; Disparity; Equity; Fairness; Inclusiveness; | ✔ |  |  |
| **434** | Goisauf, M, et al. | 2022 | Autonomy; Informed consent; Privacy; | ~ | Beneficence; Harm; Non-maleficence; Safety; | ~ | Explainability; Explicability; Intelligibility; Interpretability; Transparency; Understandability; | ✔ | Accountability; Contestability; Liability; Responsibility; | ✔ | Bias; Discrimination; Disparity; Equality; Equity; Fairness; Justice; | ✔ |  |  |
| **435** | Lesandrini, J, et al. | 2023 | Autonomy; Informed consent; Privacy; | ~ | Harm; | ~ |  |  |  |  | Bias; | ~ |  |  |
| **436** | Morgan, MB, et al. | 2023 | Autonomy; Confidentiality; Informed consent; Privacy; | ✔ | Beneficence; Harm; Non-maleficence; | ✔ | Explicability; Intelligibility; Transparency; | ✔ | Accountability; Responsibility; | ✔ | Bias; Disparity; Equality; Equity; Fairness; Justice; | ✔ |  |  |
| **437** | Abdullah, YI, et al. | 2021 | Autonomy; Confidentiality; Informed consent; Privacy; | ✔ | Beneficence; Efficacy; Harm; Non-maleficence; Safety; | ~ | Explainability; Intelligibility; Transparency; | ✔ | Accountability; Liability; Responsibility; | ✔ | Bias; Discrimination; Disparity; Equality; Equity; Fairness; Inclusiveness; Justice; | ✔ |  |  |
| **438** | Jackson, BR, et al. | 2021 | Autonomy; Informed consent; Privacy; | ✔ | Beneficence; Harm; Non-maleficence; Safety; Well-being; | ✔ | Explainability; Transparency; Understandability; | ~ | Accountability; Human oversight; Liability; Responsibility; | ✔ | Bias; Discrimination; Disparity; Equity; Fairness; Justice; | ✔ | Continuous monitoring; | ~ |
| **439** | Geis, JR, et al. | 2019 | Informed consent; Privacy; | ~ | Beneficence; Harm; Safety; Well-being; | ~ | Explainability; Transparency; Understandability; | ✔ | Accountability; Human oversight; Liability; Responsibility; | ✔ | Bias; Discrimination; Disparity; Equality; Equity; Fairness; Justice; | ✔ | Continuous monitoring; Sustainability; | ~ |
| **443** | Heyen, NB, et al. | 2021 | Autonomy; Informed consent; Privacy; | ✔ |  |  | Explainability; Transparency; | ~ | Responsibility; | ~ | Bias; Discrimination; Fairness; | ✔ |  |  |
| **444** | Dalton-Brown, S | 2020 | Autonomy; Informed consent; Privacy; | ✔ | Safety; | ~ | Explainability; Understandability; | ~ | Accountability; Liability; Responsibility; | ✔ | Bias; | ~ | Environment; Sustainability; | ~ |
| **445** | Larson, DB, et al. | 2020 | Informed consent; Privacy; | ✔ | Beneficence; Harm; Public interest; Safety; | ✔ |  |  | Accountability; Human oversight; Responsibility; | ~ | Fairness; Justice; | ~ |  |  |
| **446** | Sood, A, et al. | 2022 | Autonomy; Informed consent; | ~ | Beneficence; Harm; Non-maleficence; | ~ | Explainability; Understandability; | ~ |  |  | Bias; Equity; Fairness; Justice; | ✔ |  |  |
| **447** | Kohli, M, et al. | 2018 | Human oversight; Informed consent; Privacy; | ✔ | Harm; Safety; | ~ | Transparency; Understandability; | ~ |  |  | Bias; Discrimination; | ~ |  |  |
| **448** | Lin, M, et al. | 2023 |  |  | Harm; Well-being; | ~ |  |  |  |  | Bias; Disparity; Fairness; | ✔ |  |  |
| **450** | McInerney, C, et al. | 2022 |  |  | Safety; | ✔ |  |  |  |  |  |  |  |  |
| **451** | Reddy, S, et al. | 2021 | Autonomy; Informed consent; Privacy; | ✔ | Beneficence; Efficacy; Harm; Non-maleficence; Safety; | ✔ | Explainability; Interpretability; Transparency; Understandability; | ✔ |  |  | Bias; Disparity; Justice; | ✔ | Continuous monitoring; | ✔ |
| **454** | McCradden, M, et al. | 2023 | Informed consent; | ~ | Harm; Safety; | ~ | Explainability; Interpretability; Transparency; Understandability; | ✔ | Accountability; Liability; | ~ | Bias; Discrimination; Fairness; Justice; | ✔ |  |  |
| **455** | Bellini, V, et al. | 2023 | Privacy; | ~ | Harm; Safety; | ~ | Explainability; Interpretability; Understandability; | ✔ |  |  | Disparity; Equality; Equity; Justice; | ✔ |  |  |
| **456** | Amann, J, et al. | 2023 | Autonomy; Informed consent; Privacy; | ✔ | Harm; Safety; | ~ | Explainability; Interpretability; Transparency; Understandability; | ~ | Accountability; Liability; Responsibility; | ✔ | Bias; Discrimination; Equality; Equity; Fairness; Inclusiveness; Justice; | ~ |  |  |
| **457** | Kloka, JA, et al. | 2023 |  |  | Safety; | ~ | Explainability; | ~ | Liability; | ✔ |  |  |  |  |
| **458** | Starke, G, et al. | 2023 | Autonomy; Privacy; | ✔ | Beneficence; Harm; Non-maleficence; | ~ | Explainability; Explicability; Intelligibility; Interpretability; Transparency; Understandability; | ✔ | Accountability; Contestability; Human oversight; Liability; Responsibility; | ~ | Bias; Discrimination; Fairness; Justice; | ✔ | Environment; | ~ |
| **459** | Amann, J, et al. | 2020 | Autonomy; Informed consent; Privacy; | ✔ | Beneficence; Efficacy; Harm; Non-maleficence; Safety; Well-being; | ✔ | Explainability; Interpretability; Transparency; Understandability; | ✔ | Accountability; Liability; Responsibility; | ~ | Bias; Discrimination; Equality; Justice; | ✔ |  |  |
| **461** | Saraswat, D, et al. | 2022 | Confidentiality; Privacy; | ✔ | Efficacy; Safety; | ~ | Explainability; Interpretability; Transparency; Understandability; | ✔ | Accountability; Contestability; Responsibility; | ~ | Bias; Fairness; | ~ |  |  |
| **462** | Sabol, P, et al. | 2020 |  |  | Efficacy; | ~ | Explainability; Interpretability; Transparency; Understandability; | ✔ | Accountability; Responsibility; | ✔ |  |  |  |  |
| **463** | Singh, A, et al. | 2020 |  |  | Safety; | ~ | Explainability; Interpretability; Transparency; Understandability; | ✔ |  |  | Fairness; | ~ |  |  |
| **464** | Rasheed, K, et al. | 2022 | Autonomy; Confidentiality; Human oversight; Informed consent; Privacy; | ✔ | Beneficence; Efficacy; Harm; Non-maleficence; Safety; Well-being; | ✔ | Explainability; Explicability; Intelligibility; Interpretability; Transparency; Understandability; | ✔ | Accountability; Human supervision; Liability; Responsibility; | ✔ | Bias; Discrimination; Equity; Fairness; Inclusiveness; Justice; | ✔ | Environment; Sustainability; | ~ |
| **465** | Ursin, F, et al. | 2022 | Autonomy; Confidentiality; Human oversight; Informed consent; Privacy; | ✔ | Beneficence; Harm; Non-maleficence; Safety; Well-being; | ✔ | Explainability; Explicability; Intelligibility; Interpretability; Transparency; Understandability; | ✔ | Accountability; Liability; Responsibility; | ~ | Bias; Discrimination; Equality; Fairness; Justice; | ✔ |  |  |
| **467** | Yadav, P, et al. | 2023 |  |  | Beneficence; Efficacy; Harm; | ~ | Explainability; Interpretability; | ✔ |  |  |  |  |  |  |
| **468** | Adhikari, K, et al. | 2023 | Autonomy; Confidentiality; Human oversight; Informed consent; Privacy; | ✔ | Safety; | ~ | Transparency; Understandability; | ~ | Accountability; Human oversight; Human supervision; Liability; Responsibility; | ✔ | Bias; Disparity; Equality; Fairness; | ✔ | Continuous improvement; Continuous monitoring; | ~ |
| **469** | Stafie, CS, et al. | 2023 | Autonomy; Confidentiality; Privacy; | ~ | Harm; Safety; | ~ | Explainability; Interpretability; Transparency; Understandability; | ✔ | Liability; | ~ | Bias; | ~ |  |  |
| **470** | Suppadungsuk, S, et al. | 2023 | Confidentiality; Informed consent; Privacy; | ✔ | Beneficence; Efficacy; Safety; Well-being; | ✔ | Explainability; Transparency; Understandability; | ~ | Human oversight; | ~ | Inclusiveness; | ~ | Continuous improvement; Continuous monitoring; | ✔ |
| **471** | Jalal, S, et al. | 2021 | Informed consent; Privacy; | ~ | Safety; | ~ | Transparency; | ~ | Accountability; | ~ | Bias; Discrimination; Fairness; | ~ | Continuous monitoring; | ~ |
| **472** | Espejo, G, et al. | 2023 | Autonomy; Informed consent; Privacy; | ✔ | Efficacy; | ~ |  |  | Human oversight; Human supervision; | ~ | Bias; Disparity; Equality; Equity; | ~ |  |  |
| **473** | Garg, RK, et al. | 2023 | Privacy; | ~ | Harm; | ~ | Interpretability; | ~ |  |  | Bias; | ~ |  |  |
| **474** | Rosemann, A, et al. | 2022 | Autonomy; Informed consent; Privacy; | ✔ | Harm; Safety; Well-being; | ~ | Transparency; | ~ | Accountability; Human oversight; Responsibility; | ✔ | Bias; Discrimination; Disparity; Equality; Equity; Fairness; Inclusiveness; Justice; | ✔ | Environment; Sustainability; | ✔ |
| **475** | Arigbede, O, et al. | 2023 | Confidentiality; Privacy; | ~ | Beneficence; Efficacy; Harm; Safety; | ✔ | Understandability; | ~ |  |  | Bias; Disparity; | ✔ |  |  |
| **476** | Dossantos, J, et al. | 2023 | Confidentiality; Human oversight; Privacy; | ~ | Safety; | ~ | Transparency; | ~ |  |  | Bias; | ~ |  |  |
| **477** | Nordling, L | 2019 | Privacy; | ✔ | Harm; | ~ |  |  |  |  | Bias; Discrimination; Disparity; Equality; Equity; Fairness; | ✔ |  |  |
| **478** | Banja, J, et al. | 2023 | Confidentiality; | ~ | Harm; | ~ | Explainability; Transparency; | ~ | Accountability; Human oversight; Responsibility; | ~ | Bias; Discrimination; Disparity; Equality; Equity; Fairness; Justice; | ✔ | Continuous monitoring; | ~ |
| **479** | Ueda, D, et al. | 2023 | Autonomy; Confidentiality; Informed consent; Privacy; | ✔ | Beneficence; Harm; Non-maleficence; Safety; | ~ | Explainability; Interpretability; Transparency; Understandability; | ✔ | Accountability; Liability; Responsibility; | ✔ | Bias; Discrimination; Disparity; Equity; Fairness; Inclusiveness; Justice; | ✔ | Continuous monitoring; | ✔ |
| **480** | Stanley, EAM, et al. | 2022 |  |  |  |  | Explainability; Interpretability; Understandability; | ✔ |  |  | Bias; Disparity; Equality; Fairness; | ✔ |  |  |
| **481** | Donnelly, DL | 2022 | Autonomy; Confidentiality; Informed consent; Privacy; | ✔ | Beneficence; Efficacy; Harm; Safety; Well-being; | ~ | Explainability; Explicability; Transparency; Understandability; | ✔ | Accountability; Human oversight; Liability; Redress; Responsibility; | ✔ | Bias; Discrimination; Equality; Fairness; Inclusiveness; Justice; | ~ | Sustainability; | ~ |
| **482** | Shaw, JA, et al. | 2021 | Informed consent; | ✔ | Harm; Safety; | ~ |  |  | Responsibility; | ~ | Bias; Equity; | ✔ |  |  |
| **483** | Abramoff, MD, et al. | 2022 | Autonomy; Confidentiality; Human oversight; Privacy; | ✔ | Beneficence; Efficacy; Harm; Non-maleficence; Safety; | ✔ | Explainability; Transparency; Understandability; | ✔ | Accountability; Human oversight; Liability; Responsibility; | ✔ | Bias; Disparity; Equity; Justice; | ✔ | Continuous monitoring; | ✔ |
| **484** | Khoury, P, et al. | 2022 | Informed consent; Privacy; | ~ | Efficacy; Harm; Safety; Well-being; | ~ | Explainability; Interpretability; Transparency; | ~ | Accountability; Human oversight; Liability; | ~ | Bias; Disparity; Equity; Fairness; | ✔ |  |  |
| **485** | MacKay, C, et al. | 2023 | Autonomy; Informed consent; Privacy; | ✔ | Harm; Non-maleficence; Safety; Well-being; | ✔ | Explainability; Interpretability; Transparency; Understandability; | ✔ | Accountability; Human oversight; Liability; Responsibility; | ✔ | Bias; Equity; Fairness; Justice; | ✔ |  |  |
| **486** | Rojas, JC, et al. | 2022 |  |  | Harm; Safety; Well-being; | ~ | Interpretability; Transparency; Understandability; | ✔ |  |  | Bias; Discrimination; Disparity; Equality; Equity; Fairness; Justice; | ✔ | Continuous improvement; Continuous monitoring; | ✔ |
| **487** | Cagliero, D, et al. | 2023 | Informed consent; Privacy; | ✔ | Beneficence; Efficacy; Harm; Safety; | ✔ | Interpretability; Transparency; Understandability; | ✔ | Accountability; Human oversight; | ~ | Bias; Equality; Equity; Fairness; Inclusiveness; Justice; | ✔ |  |  |
| **488** | Griffin, AC, et al. | 2020 | Privacy; | ~ | Safety; | ✔ | Interpretability; Transparency; | ✔ |  |  | Bias; Disparity; | ~ | Environment; | ~ |
| **489** | Gundersen, T, et al. | 2022 | Autonomy; Human oversight; Privacy; | ~ | Harm; | ~ | Explainability; Interpretability; Transparency; Understandability; | ✔ | Accountability; Responsibility; | ✔ | Bias; Discrimination; Fairness; | ✔ |  |  |
| **491** | Arora, A, et al. | 2022 | Confidentiality; Privacy; | ✔ |  |  |  |  |  |  | Bias; | ~ | Environment; | ~ |
| **493** | Paladugu, PS, et al. | 2023 | Confidentiality; Informed consent; Privacy; | ✔ | Safety; | ~ | Interpretability; | ~ | Accountability; Human oversight; Liability; Responsibility; | ~ | Bias; Discrimination; Equity; Fairness; Justice; | ✔ | Continuous monitoring; | ~ |
| **494** | Koohi-Moghadam, M, et al. | 2023 | Privacy; | ✔ | Harm; | ~ | Interpretability; Transparency; | ~ | Responsibility; | ~ | Bias; Equity; | ✔ |  |  |
| **495** | Ray, PP | 2023 | Confidentiality; Privacy; | ~ | Beneficence; Harm; | ~ | Transparency; | ~ |  |  | Bias; | ~ |  |  |
| **496** | Goodman, KW, et al. | 2023 | Autonomy; Confidentiality; Informed consent; Privacy; | ~ | Beneficence; Safety; Well-being; | ~ | Explainability; Intelligibility; Transparency; Understandability; | ~ | Accountability; Human oversight; Human supervision; Liability; Responsibility; | ✔ | Bias; Discrimination; Disparity; Equity; Fairness; Inclusiveness; | ~ | Climate; Environment; Responsiveness; Sustainability; | ~ |
| **497** | Hossain, MM, et al. | 2019 |  |  | Efficacy; Safety; | ~ |  |  |  |  | Disparity; Equality; | ~ | Sustainability; | ~ |
| **499** | Reddy, S, et al. | 2020 | Autonomy; Confidentiality; Informed consent; Privacy; | ✔ | Beneficence; Efficacy; Harm; Non-maleficence; Safety; Well-being; | ~ | Explainability; Interpretability; Transparency; Understandability; | ✔ | Accountability; Human oversight; Liability; Responsibility; | ✔ | Bias; Discrimination; Disparity; Equity; Fairness; Justice; | ✔ | Continuous monitoring; Responsiveness; | ✔ |
| **500** | Ho, CWL, et al. | 2019 | Autonomy; Confidentiality; Informed consent; Privacy; | ✔ | Harm; Safety; | ✔ | Explainability; Transparency; | ~ | Accountability; Human oversight; Liability; Responsibility; | ✔ | Bias; Discrimination; Equity; Fairness; | ✔ | Continuous monitoring; | ~ |
| **501** | Liao, F, et al. | 2022 | Autonomy; | ~ | Beneficence; Harm; Non-maleficence; Safety; | ~ | Explainability; Interpretability; | ~ | Accountability; Human oversight; Responsibility; | ✔ | Equity; Fairness; Justice; | ✔ | Continuous monitoring; Sustainability; | ✔ |
| **502** | Mathews, DJH, et al. | 2022 |  |  | Beneficence; Harm; Safety; Well-being; | ~ |  |  | Responsibility; | ~ | Equality; Equity; Fairness; Justice; | ~ |  |  |
| **503** | Verma, AA, et al. | 2023 | Autonomy; Informed consent; Privacy; | ~ | Harm; Safety; | ✔ | Explainability; Interpretability; Transparency; Understandability; | ✔ | Accountability; Liability; | ~ | Bias; Disparity; Equality; Equity; Fairness; | ✔ | Continuous monitoring; | ✔ |
| **504** | de Hond, AAH, et al. | 2022 | Confidentiality; Privacy; | ✔ | Safety; | ~ | Explainability; Interpretability; Transparency; Understandability; | ✔ | Accountability; Human oversight; Responsibility; | ~ | Bias; Equality; Fairness; Inclusiveness; | ✔ | Continuous monitoring; | ✔ |
| **505** | Crossnohere, NL, et al. | 2022 | Autonomy; Privacy; | ~ | Efficacy; Harm; Safety; | ~ | Interpretability; Transparency; Understandability; | ✔ | Human oversight; | ✔ | Bias; Equity; Fairness; | ~ |  |  |
| **506** | Chiang, S, et al. | 2021 | Autonomy; Privacy; | ✔ | Beneficence; Harm; Non-maleficence; Safety; | ✔ | Explainability; Explicability; Transparency; Understandability; | ✔ | Human oversight; Human supervision; Liability; Responsibility; | ~ | Bias; Discrimination; Disparity; Equality; Equity; Justice; | ✔ | Carbon; Continuous monitoring; Ecological footprint; | ✔ |
| **507** | Holm, S | 2022 | Autonomy; | ✔ | Beneficence; Harm; | ✔ | Explainability; Interpretability; Understandability; | ✔ |  |  | Bias; Equality; Fairness; Justice; | ✔ |  |  |
| **509** | Nashwan, AJ, et al. | 2023 | Autonomy; Confidentiality; Informed consent; Privacy; | ✔ | Harm; Safety; Well-being; | ~ | Transparency; | ~ | Accountability; Human oversight; Human supervision; Liability; Responsibility; | ~ | Bias; Discrimination; Disparity; Equity; Fairness; Inclusiveness; Justice; | ✔ |  |  |
| **510** | Bragazzi, NL, et al. | 2022 | Privacy; | ~ |  |  |  |  |  |  | Bias; Disparity; Equality; | ~ |  |  |
| **511** | Nashwan, AJ, et al. | 2023 | Confidentiality; Privacy; | ~ |  |  | Explainability; Interpretability; Transparency; Understandability; | ~ | Accountability; Responsibility; | ~ | Bias; Equality; Fairness; | ~ |  |  |
| **513** | Racine, E, et al. | 2019 | Confidentiality; Informed consent; Privacy; | ✔ |  |  | Explainability; Transparency; | ~ | Accountability; Responsibility; | ~ | Bias; Discrimination; | ~ | Continuous monitoring; | ~ |
| **514** | Martinho, A, et al. | 2021 | Autonomy; Confidentiality; Human oversight; Informed consent; Privacy; | ✔ | Safety; | ~ | Explainability; Interpretability; Transparency; Understandability; | ✔ | Accountability; Human oversight; Liability; | ✔ | Bias; Discrimination; Equality; Equity; Fairness; Justice; | ✔ |  |  |
| **515** | Reich, C, et al. | 2023 | Autonomy; Confidentiality; Informed consent; Privacy; | ✔ | Beneficence; Harm; Non-maleficence; Safety; | ~ | Explainability; Interpretability; Transparency; Understandability; | ✔ | Human supervision; Responsibility; | ~ | Bias; Discrimination; Fairness; Justice; | ✔ |  |  |
| **516** | Al Meslamani, AZ | 2023 | Informed consent; Privacy; | ~ | Efficacy; | ~ |  |  |  |  | Bias; Equality; Equity; | ✔ |  |  |
| **517** | Nabi, J | 2018 | Autonomy; Confidentiality; Informed consent; Privacy; | ✔ | Harm; | ~ |  |  | Accountability; Responsibility; | ~ | Bias; Equality; Fairness; | ~ |  |  |
| **518** | Briganti, G | 2023 | Privacy; | ~ | Beneficence; | ~ | Interpretability; Understandability; | ~ |  |  |  |  |  |  |
| **519** | Grote, T, et al. | 2022 | Autonomy; | ~ | Beneficence; Harm; Safety; | ~ | Explainability; Intelligibility; Interpretability; Understandability; | ✔ | Accountability; Liability; Responsibility; | ~ |  |  |  |  |
| **521** | Anderson, M, et al. | 2019 |  |  |  |  | Transparency; Understandability; | ✔ |  |  |  |  |  |  |
| **522** | Schiff, D, et al. | 2019 | Informed consent; | ✔ | Harm; Safety; | ~ | Explainability; Intelligibility; Transparency; Understandability; | ✔ | Liability; Responsibility; | ✔ | Bias; | ~ | Continuous monitoring; | ~ |
| **523** | Baeroe, K, et al. | 2020 | Autonomy; Human oversight; Privacy; | ~ | Beneficence; Harm; Safety; Well-being; | ✔ | Explicability; Transparency; Understandability; | ~ | Accountability; Responsibility; | ~ | Bias; Discrimination; Equality; Equity; Fairness; Justice; | ✔ | Environment; | ~ |
| **524** | Nagy, M, et al. | 2020 |  |  | Harm; | ✔ | Explainability; | ~ |  |  |  |  |  |  |
| **527** | Iniesta, R. | 2023 | Autonomy; Informed consent; Privacy; | ✔ | Beneficence; Efficacy; Harm; Non-maleficence; Safety; Well-being; | ✔ | Explainability; Intelligibility; Interpretability; Transparency; Understandability; | ✔ | Accountability; Human supervision; Liability; Responsibility; | ✔ | Bias; Discrimination; Equality; Equity; Fairness; Inclusiveness; Justice; | ✔ | Responsiveness; Sustainability; | ~ |
| **528** | Coppola, F, et al. | 2021 | Autonomy; Confidentiality; Informed consent; Privacy; | ✔ | Beneficence; Harm; Safety; Well-being; | ~ | Explainability; Interpretability; Transparency; Understandability; | ✔ | Liability; Responsibility; | ✔ | Bias; Discrimination; Equality; | ✔ |  |  |
| **529** | Rodrigo, GH | 2023 | Privacy; | ~ | Efficacy; Safety; | ~ | Explainability; Interpretability; Understandability; | ~ |  |  | Bias; | ~ |  |  |
| **530** | Hallowell, N, et al. | 2022 | Autonomy; Confidentiality; Human oversight; Informed consent; Privacy; | ~ | Harm; | ~ | Explainability; Interpretability; Transparency; Understandability; | ✔ | Accountability; Responsibility; | ~ | Bias; | ✔ | Continuous monitoring; | ~ |
| **531** | Kempt, H, et al. | 2023 | Autonomy; | ✔ |  |  | Explainability; Intelligibility; Interpretability; Understandability; | ✔ | Liability; Responsibility; | ✔ |  |  |  |  |
| **532** | Char, DS, et al. | 2020 | Autonomy; Privacy; | ~ | Harm; Safety; | ✔ | Explainability; Interpretability; Transparency; Understandability; | ✔ | Accountability; Human oversight; Human supervision; Liability; Responsibility; | ~ | Bias; Discrimination; Equity; Fairness; | ✔ |  |  |
| **534** | Chakrabarty, N, et al. | 2023 | Autonomy; Privacy; | ~ | Beneficence; Harm; Non-maleficence; Safety; Well-being; | ~ | Explainability; Explicability; Interpretability; Transparency; | ✔ | Accountability; Responsibility; | ~ | Bias; Equality; Fairness; Justice; | ~ |  |  |
| **535** | Walsh, SLF, et al. | 2020 | Confidentiality; Privacy; | ~ |  |  | Interpretability; Transparency; Understandability; | ~ | Accountability; | ~ | Bias; Disparity; | ~ |  |  |
| **536** | Choudhury, A, et al. | 2022 | Informed consent; Privacy; | ~ | Harm; Safety; | ✔ | Explainability; Interpretability; Transparency; Understandability; | ~ | Accountability; Liability; Responsibility; | ✔ | Bias; | ~ |  |  |
| **539** | Istasy, P, et al. | 2022 | Informed consent; Privacy; | ~ | Efficacy; Harm; | ~ | Interpretability; Transparency; | ~ |  |  | Bias; Discrimination; Disparity; Equality; Equity; Fairness; | ✔ |  |  |
| **540** | Abdallah, S, et al. | 2023 | Informed consent; Privacy; | ~ | Efficacy; | ~ | Interpretability; Transparency; | ~ | Responsibility; | ~ | Bias; Equity; Fairness; | ~ |  |  |
| **541** | Vanhonacker, D, et al. | 2022 | Informed consent; Privacy; | ✔ | Harm; Safety; | ✔ |  |  | Liability; | ~ | Bias; | ✔ |  |  |
| **542** | Mesko, B, et al. | 2023 | Autonomy; Confidentiality; Informed consent; Privacy; | ✔ | Harm; Safety; | ~ | Explainability; Interpretability; Transparency; Understandability; | ~ | Accountability; Human oversight; Liability; Responsibility; | ✔ | Bias; Discrimination; Disparity; Equity; Fairness; | ✔ | Continuous monitoring; | ~ |
| **543** | Gama, F, et al. | 2022 | Autonomy; Human oversight; Informed consent; Privacy; | ~ | Beneficence; Non-maleficence; Safety; Well-being; | ~ | Explainability; Explicability; Interpretability; Transparency; | ~ | Accountability; Responsibility; | ~ | Bias; Discrimination; Equality; Equity; Fairness; Justice; | ~ |  |  |
| **544** | Gleeson, F, et al. | 2023 | Confidentiality; | ~ | Efficacy; Harm; Safety; | ✔ | Explainability; Transparency; | ~ | Accountability; Liability; Responsibility; | ✔ | Bias; | ~ |  |  |
| **545** | Filipow, N, et al. | 2022 |  |  | Efficacy; Harm; Safety; | ✔ | Explainability; Interpretability; Transparency; | ✔ | Accountability; Liability; Responsibility; | ✔ | Bias; Disparity; Equity; | ✔ |  |  |
| **546** | Char, DS, et al. | 2018 | Confidentiality; | ✔ |  |  |  |  | Responsibility; | ~ | Bias; Discrimination; | ✔ |  |  |
| **547** | Ben Ali, W, et al. | 2021 | Informed consent; Privacy; | ✔ | Public interest; Safety; | ~ | Explainability; Interpretability; Understandability; | ✔ | Human oversight; Liability; Responsibility; | ~ | Bias; Equality; | ~ | Continuous monitoring; | ✔ |
| **548** | Mese, I, et al. | 2023 | Confidentiality; Privacy; | ~ | Harm; | ~ | Transparency; Understandability; | ~ | Accountability; Human oversight; Responsibility; | ~ | Bias; Discrimination; Equality; Justice; | ✔ | Continuous improvement; Continuous monitoring; | ~ |
| **549** | Felmingham, C, et al. | 2022 |  |  | Safety; | ✔ |  |  |  |  |  |  |  |  |
| **550** | Montemayor, C, et al. | 2022 | Autonomy; | ✔ | Beneficence; Well-being; | ~ |  |  |  |  |  |  |  |  |
| **551** | Biller-Andorno, N, et al. | 2022 | Autonomy; | ✔ | Beneficence; Non-maleficence; Safety; | ~ | Explainability; Interpretability; Transparency; Understandability; | ~ | Responsibility; | ~ |  |  |  |  |
| **552** | Bazoukis, G, et al. | 2022 | Informed consent; Privacy; | ✔ | Efficacy; Harm; Safety; | ✔ | Explainability; Interpretability; Transparency; Understandability; | ✔ | Accountability; Human oversight; Liability; Responsibility; | ✔ | Bias; Disparity; Equity; Fairness; | ✔ | Continuous improvement; Continuous monitoring; | ✔ |
| **553** | Binkley, CE, et al. | 2023 | Autonomy; Confidentiality; Informed consent; Privacy; | ✔ | Beneficence; Harm; | ✔ |  |  | Liability; | ~ | Bias; | ~ |  |  |
| **554** | Taribagil, P, et al. | 2023 | Autonomy; Informed consent; Privacy; | ✔ | Harm; Non-maleficence; Safety; Well-being; | ~ | Explainability; Interpretability; Transparency; Understandability; | ✔ | Accountability; Liability; Responsibility; | ✔ | Bias; Disparity; Equality; Equity; Fairness; Inclusiveness; | ✔ | Continuous monitoring; | ✔ |
| **555** | Recht, MP, et al. | 2020 | Informed consent; Privacy; | ~ | Harm; Safety; | ~ | Explainability; Intelligibility; Interpretability; Transparency; Understandability; | ✔ | Accountability; Liability; Responsibility; | ~ | Bias; Equality; Fairness; | ✔ |  |  |
| **556** | Nyariro, M, et al. | 2023 |  |  | Harm; | ~ |  |  |  |  | Bias; Discrimination; Equality; Equity; Fairness; | ✔ |  |  |
| **557** | Kumar, R, et al. | 2021 | Privacy; | ✔ |  |  | Transparency; | ~ |  |  |  |  |  |  |
| **558** | Che, Z, et al. | 2016 |  |  |  |  | Interpretability; Understandability; | ✔ |  |  |  |  |  |  |
| **559** | Afnan, MAM, et al. | 2021 | Autonomy; Informed consent; Privacy; | ✔ | Harm; Safety; Well-being; | ✔ | Explainability; Interpretability; Transparency; Understandability; | ✔ | Accountability; Human oversight; Liability; Responsibility; | ✔ | Bias; | ~ |  |  |
| **560** | Wong, RS, et al. | 2023 | Confidentiality; Informed consent; Privacy; | ✔ | Safety; | ~ | Explainability; Transparency; | ~ | Accountability; Liability; Responsibility; | ✔ | Bias; Discrimination; Fairness; | ✔ |  |  |
| **561** | Drukker, L, et al. | 2021 | Informed consent; Privacy; | ✔ | Safety; | ~ | Explainability; Understandability; | ✔ | Accountability; Liability; Responsibility; | ✔ | Bias; | ~ |  |  |
| **563** | Chapalain, X, et al. | 2019 | Confidentiality; Informed consent; Privacy; | ~ | Well-being; | ~ |  |  |  |  | Fairness; | ~ |  |  |
| **565** | Gampala, S, et al. | 2020 | Privacy; | ~ | Harm; Safety; Well-being; | ~ | Explainability; Transparency; Understandability; | ✔ | Accountability; Responsibility; | ~ | Bias; Disparity; Equity; Fairness; | ✔ |  |  |
| **567** | Ren, Y, et al. | 2023 | Privacy; | ~ |  |  | Interpretability; | ~ |  |  | Disparity; | ~ |  |  |
| **568** | Hardt, M, et al. | 2020 | Informed consent; | ~ |  |  | Explainability; Transparency; Understandability; | ~ | Accountability; | ~ | Bias; Disparity; Equality; Equity; Fairness; Justice; | ✔ | Continuous monitoring; | ~ |
| **569** | Hummel, P, et al. | 2020 | Autonomy; Informed consent; Privacy; | ✔ | Beneficence; Harm; Non-maleficence; Safety; Well-being; | ~ | Explainability; Transparency; Understandability; | ~ | Responsibility; | ~ | Bias; Discrimination; Disparity; Equality; Equity; Fairness; Justice; | ✔ |  |  |
| **570** | Love, CS | 2023 | Autonomy; Privacy; | ✔ | Beneficence; Harm; Non-maleficence; Safety; | ~ | Explainability; Transparency; Understandability; | ✔ | Accountability; Responsibility; | ~ | Bias; Discrimination; Equality; Fairness; Justice; | ✔ |  |  |
| **571** | Rueda, J, et al. | 2022 | Autonomy; Privacy; | ~ | Beneficence; Harm; Non-maleficence; Safety; Well-being; | ~ | Explainability; Explicability; Intelligibility; Interpretability; Transparency; Understandability; | ✔ | Accountability; Responsibility; | ✔ | Bias; Discrimination; Equality; Equity; Fairness; Justice; | ✔ | Environment; | ~ |
| **572** | Jotterand, F, et al. | 2020 | Autonomy; Informed consent; | ✔ | Beneficence; Safety; Well-being; | ~ | Transparency; | ~ | Accountability; Responsibility; | ~ |  |  |  |  |
| **573** | Lovejoy, CA, et al. | 2022 | Informed consent; Privacy; | ✔ | Safety; | ~ | Explainability; Interpretability; Transparency; | ✔ | Accountability; | ~ | Bias; Discrimination; | ✔ |  |  |
| **574** | Peirce, AG, et al. | 2020 | Autonomy; Human oversight; Privacy; | ~ | Harm; Safety; | ~ | Explainability; Transparency; Understandability; | ✔ |  |  | Bias; Discrimination; Justice; | ~ |  |  |
| **575** | Abi-Rafeh, J, et al. | 2023 | Confidentiality; Human oversight; Informed consent; Privacy; | ~ | Safety; | ~ | Transparency; | ~ | Accountability; Human oversight; | ~ | Bias; Equity; | ~ |  |  |
| **576** | Betzler, BK, et al. | 2023 | Informed consent; Privacy; | ✔ | Safety; | ~ |  |  | Liability; | ~ | Bias; Equity; | ~ |  |  |
| **577** | Romano, MF, et al. | 2023 | Autonomy; Confidentiality; Informed consent; Privacy; | ✔ | Harm; Safety; | ~ | Explainability; Interpretability; Transparency; Understandability; | ~ |  |  | Bias; Discrimination; Disparity; Fairness; | ✔ | Continuous monitoring; | ~ |
| **578** | Akinci D'Antonoli, T, et al. | 2023 | Human oversight; Privacy; | ✔ | Harm; Safety; | ✔ | Explainability; Interpretability; Transparency; | ✔ |  |  | Bias; Fairness; | ✔ | Continuous improvement; Environment; | ~ |
| **580** | Naik, N, et al. | 2022 | Informed consent; Privacy; | ~ | Harm; Safety; Well-being; | ~ | Explainability; Transparency; Understandability; | ~ | Accountability; Liability; Responsibility; | ✔ | Bias; Discrimination; Equity; Fairness; Inclusiveness; Justice; | ✔ |  |  |
| **581** | Jobson, D, et al. | 2022 | Informed consent; Privacy; | ✔ | Efficacy; Harm; Safety; | ~ | Transparency; Understandability; | ~ | Accountability; Liability; Responsibility; | ✔ |  |  |  |  |
| **582** | Sihlahla, I, et al. | 2023 | Confidentiality; Human oversight; Informed consent; Privacy; | ✔ | Beneficence; Efficacy; Harm; Safety; | ✔ | Explainability; Interpretability; Transparency; Understandability; | ✔ | Accountability; Human oversight; Human supervision; Liability; Redress; | ✔ | Bias; Discrimination; Disparity; Fairness; Inclusiveness; Justice; | ✔ | Continuous monitoring; | ~ |
| **583** | Abramoff, MD, et al. | 2020 | Autonomy; Human oversight; Informed consent; Privacy; | ~ | Beneficence; Efficacy; Harm; Non-maleficence; Safety; | ✔ | Transparency; Understandability; | ~ | Accountability; Liability; Responsibility; | ✔ | Bias; Disparity; Equity; Fairness; Justice; | ✔ |  |  |
| **584** | Ursin, F, et al. | 2023 | Autonomy; Human oversight; Informed consent; | ✔ | Harm; | ~ | Explainability; Explicability; Intelligibility; Interpretability; Transparency; Understandability; | ✔ | Accountability; Responsibility; | ~ | Bias; Discrimination; Equality; | ✔ |  |  |
| **586** | Yu, P, et al. | 2023 | Human oversight; Privacy; | ✔ | Efficacy; Harm; Safety; | ✔ | Explainability; Transparency; | ~ | Accountability; Contestability; Liability; Redress; Responsibility; | ~ | Bias; Disparity; Equality; Equity; Fairness; Inclusiveness; | ✔ |  |  |
| **588** | Hatherley, JJ | 2020 |  |  |  |  |  |  | Responsibility; | ✔ | Bias; | ~ |  |  |
| **589** | Cutillo, CM, et al. | 2020 | Privacy; | ~ | Beneficence; Efficacy; Safety; | ~ | Explainability; Interpretability; Transparency; Understandability; | ✔ |  |  | Bias; Disparity; Equality; Equity; Fairness; | ✔ | Continuous improvement; Continuous monitoring; | ~ |
| **591** | Kerr, WT, et al. | 2023 |  |  | Efficacy; Safety; | ~ | Explainability; | ~ | Liability; Responsibility; | ~ | Bias; Disparity; Equity; | ✔ |  |  |
| **592** | Rubinger, L, et al. | 2023 |  |  | Beneficence; Safety; | ~ | Explainability; Interpretability; Transparency; Understandability; | ✔ | Accountability; Human oversight; | ~ | Equality; Equity; Justice; | ~ | Continuous monitoring; | ~ |
| **593** | Vollmer, S, et al. | 2020 | Privacy; | ~ | Beneficence; Efficacy; Harm; Safety; | ~ | Explainability; Interpretability; Transparency; Understandability; | ✔ | Responsibility; | ~ | Bias; Disparity; Equality; Equity; Fairness; | ✔ | Continuous monitoring; | ✔ |
| **595** | Azencott, CA | 2018 | Confidentiality; Privacy; | ✔ |  |  |  |  |  |  |  |  |  |  |
| **597** | Fiste, O, et al. | 2022 | Privacy; | ~ | Public interest; Safety; | ~ | Interpretability; Transparency; | ~ |  |  | Fairness; | ~ |  |  |
| **599** | Bradley, GRE, et al. | 2023 |  |  | Harm; | ~ | Intelligibility; Interpretability; Transparency; Understandability; | ✔ | Accountability; Responsibility; | ~ |  |  |  |  |
| **600** | Wahab, H, et al. | 2023 | Informed consent; Privacy; | ~ |  |  | Explainability; Intelligibility; Interpretability; Transparency; Understandability; | ✔ | Accountability; Contestability; Liability; | ~ | Bias; Fairness; Justice; | ~ |  |  |
| **601** | Badrick, T, et al. | 2019 | Privacy; | ✔ | Harm; Safety; | ~ | Understandability; | ~ |  |  |  |  |  |  |
| **603** | Adlung, L, et al. | 2021 | Privacy; | ~ |  |  | Explainability; Transparency; Understandability; | ~ |  |  |  |  |  |  |
| **604** | Habehh, H, et al. | 2021 | Human oversight; Privacy; | ~ |  |  | Interpretability; Transparency; | ~ |  |  | Disparity; | ~ |  |  |
| **605** | Vayena, E, et al. | 2018 | Autonomy; Informed consent; Privacy; | ✔ | Efficacy; Harm; Safety; | ~ | Explainability; Interpretability; Transparency; | ✔ | Accountability; Human oversight; Liability; | ~ | Bias; Disparity; Equity; Fairness; | ✔ | Continuous improvement; Continuous monitoring; | ~ |
| **606** | Yoon, CH, et al. | 2022 | Privacy; | ~ | Efficacy; Harm; Safety; | ~ | Explainability; Explicability; Interpretability; Transparency; Understandability; | ✔ | Accountability; Liability; Redress; Responsibility; | ✔ | Bias; Equality; Equity; Justice; | ✔ |  |  |
| **607** | Adida, S, et al. | 2023 | Privacy; | ~ |  |  | Explainability; Interpretability; | ~ | Accountability; Human oversight; Liability; Responsibility; | ~ | Bias; | ✔ |  |  |
| **608** | de Miguel, I, et al. | 2020 | Autonomy; Confidentiality; Informed consent; Privacy; | ✔ | Harm; Safety; | ~ | Explainability; Intelligibility; Transparency; Understandability; | ✔ | Accountability; Responsibility; | ~ | Bias; Discrimination; Fairness; Justice; | ~ |  |  |
| **609** | Upadhyaya, DP, et al. | 2023 |  |  |  |  | Interpretability; Transparency; Understandability; | ✔ |  |  |  |  |  |  |
| **611** | Dabi, A, et al. | 2020 | Privacy; | ~ |  |  | Explainability; Interpretability; Transparency; Understandability; | ✔ | Responsibility; | ~ |  |  |  |  |
| **612** | Ostberg, NP, et al. | 2021 | Human oversight; Informed consent; Privacy; | ~ | Efficacy; | ~ | Interpretability; Understandability; | ✔ |  |  | Bias; Equity; | ✔ | Continuous monitoring; | ~ |
| **613** | Radovanovic, S, et al. | 2019 |  |  |  |  |  |  |  |  | Bias; Discrimination; Equality; Fairness; | ✔ |  |  |
| **614** | Pesapane, F, et al. | 2022 | Informed consent; | ~ |  |  | Interpretability; Understandability; | ~ | Accountability; Liability; | ~ | Bias; Equality; | ~ | Continuous monitoring; | ~ |
| **615** | Oliva, A, et al. | 2021 | Informed consent; Privacy; | ✔ | Harm; | ✔ | Explainability; Interpretability; Transparency; Understandability; | ✔ | Accountability; Human oversight; Liability; Responsibility; | ✔ | Discrimination; Equality; Fairness; | ~ | Environment; Sustainability; | ~ |
| **616** | Stewart, C, et al. | 2021 | Autonomy; Confidentiality; Human oversight; Informed consent; Privacy; | ✔ | Beneficence; Harm; Non-maleficence; Safety; Well-being; | ✔ | Explainability; Intelligibility; Transparency; Understandability; | ✔ | Accountability; Human supervision; Liability; Redress; Responsibility; | ✔ | Bias; Discrimination; Disparity; Equality; Equity; Fairness; Inclusiveness; Justice; | ✔ | Continuous monitoring; Environment; Sustainability; | ~ |
| **617** | Busch, F, et al. | 2023 |  |  |  |  |  |  |  |  | Bias; Disparity; Equality; Equity; Fairness; Inclusiveness; | ✔ |  |  |
| **620** | Corsello, A, et al. | 2023 | Informed consent; Privacy; | ~ | Harm; Safety; | ~ | Explainability; Transparency; Understandability; | ~ |  |  | Bias; Disparity; Fairness; | ~ |  |  |
| **621** | Hille, EM, et al. | 2023 | Autonomy; Confidentiality; Informed consent; | ✔ | Beneficence; Harm; Non-maleficence; | ~ | Explainability; Transparency; Understandability; | ~ | Accountability; Human supervision; Liability; Responsibility; | ✔ |  |  |  |  |
| **622** | Mainz, JT | 2023 |  |  |  |  | Transparency; | ~ |  |  |  |  |  |  |
| **623** | Tang, L, et al. | 2023 | Autonomy; Confidentiality; Informed consent; Privacy; | ✔ | Beneficence; Non-maleficence; Safety; Well-being; | ✔ | Explainability; Transparency; | ~ | Accountability; Liability; Responsibility; | ✔ | Bias; Discrimination; Disparity; Equality; Equity; Fairness; Justice; | ✔ |  |  |
| **624** | Keskinbora, KH. | 2019 | Autonomy; Informed consent; Privacy; | ✔ | Beneficence; Harm; Non-maleficence; Safety; Well-being; | ✔ | Interpretability; Transparency; Understandability; | ~ | Responsibility; | ~ | Bias; Discrimination; Equality; Fairness; Inclusiveness; Justice; | ~ | Continuous monitoring; | ~ |
| **625** | Loh, E | 2018 | Informed consent; Privacy; | ✔ | Safety; | ~ |  |  | Liability; Responsibility; | ~ | Bias; | ✔ |  |  |
| **627** | DeCamp, M, et al. | 2023 |  |  | Harm; | ~ | Explainability; Interpretability; Transparency; Understandability; | ✔ | Liability; Responsibility; | ~ | Bias; Disparity; Equity; Fairness; Inclusiveness; | ✔ |  |  |
| **629** | Fasterholdt, I, et al. | 2022 | Autonomy; Confidentiality; Human oversight; Privacy; | ~ | Beneficence; Harm; Safety; | ✔ | Transparency; | ~ | Accountability; Responsibility; | ~ | Equity; | ~ | Continuous monitoring; | ~ |
| **630** | Tran, BX, et al. | 2019 | Confidentiality; Privacy; | ~ | Safety; | ~ |  |  | Liability; | ~ | Bias; Equality; | ~ |  |  |
| **631** | Gravesteijn, BY, et al. | 2022 |  |  | Harm; Safety; | ✔ | Explainability; Interpretability; | ~ |  |  | Bias; | ~ |  |  |
| **632** | Hindocha, S, et al. | 2022 | Autonomy; Informed consent; | ✔ | Beneficence; Harm; Non-maleficence; Safety; Well-being; | ~ | Explainability; Transparency; Understandability; | ✔ | Accountability; Responsibility; | ✔ | Bias; Fairness; Justice; | ~ |  |  |
| **633** | Palmer, A, et al. | 2023 | Autonomy; Human oversight; Privacy; | ~ | Beneficence; Harm; Non-maleficence; Safety; Well-being; | ~ | Explicability; Transparency; | ~ | Accountability; Human oversight; Responsibility; | ~ | Bias; Equality; Equity; Fairness; Justice; | ~ | Continuous monitoring; Environment; | ~ |
| **634** | van de Sande, D, et al. | 2022 |  |  | Safety; | ~ | Transparency; Understandability; | ~ | Responsibility; | ~ | Bias; | ~ |  |  |
| **635** | Smallman, M | 2022 | Informed consent; Privacy; | ✔ | Beneficence; Harm; Non-maleficence; | ✔ | Transparency; | ✔ | Accountability; Liability; Responsibility; | ✔ | Bias; Discrimination; Equality; Equity; Fairness; Justice; | ✔ | Environment; Sustainability; | ✔ |
| **636** | Vo, V, et al. | 2023 | Autonomy; Human oversight; Privacy; | ✔ | Efficacy; Harm; Safety; Well-being; | ✔ | Explainability; Interpretability; Transparency; Understandability; | ✔ | Accountability; Human supervision; Liability; Responsibility; | ✔ | Bias; Disparity; Equity; Fairness; | ✔ |  |  |
| **637** | Pammi, M, et al. | 2023 | Autonomy; Privacy; | ~ | Beneficence; Harm; Non-maleficence; | ~ | Explainability; Interpretability; | ~ |  |  | Fairness; Justice; | ~ |  |  |
| **638** | Korkmaz Kirakli, E, et al. | 2023 |  |  |  |  |  |  | Liability; Responsibility; | ~ |  |  |  |  |
| **640** | Ibrahim, M, et al. | 2023 | Autonomy; Informed consent; Privacy; | ~ |  |  | Explainability; Explicability; Transparency; Understandability; | ~ |  |  | Bias; | ~ |  |  |
| **642** | Song, B, et al. | 2023 | Autonomy; Informed consent; Privacy; | ✔ | Safety; | ~ | Interpretability; Understandability; | ✔ | Human supervision; Responsibility; | ~ | Bias; Fairness; | ~ |  |  |
| **643** | Vandemeulebroucke, T, et al. | 2022 | Autonomy; Privacy; | ~ | Beneficence; Efficacy; Non-maleficence; | ~ |  |  |  |  | Justice; | ~ | Environment; | ✔ |
| **644** | Cresswell, K, et al. | 2023 | Autonomy; Confidentiality; Informed consent; Privacy; | ✔ | Safety; | ~ | Interpretability; Transparency; Understandability; | ✔ | Accountability; | ~ | Bias; Disparity; Equity; Fairness; Justice; | ✔ | Continuous monitoring; | ~ |
| **645** | Monsour, R, et al. | 2022 | Privacy; | ✔ |  |  | Explainability; Transparency; Understandability; | ✔ |  |  |  |  |  |  |
| **646** | McCradden, MD, et al. | 2023 |  |  | Beneficence; Efficacy; Harm; Safety; | ✔ | Explainability; Interpretability; Transparency; Understandability; | ✔ | Human oversight; | ~ | Bias; Disparity; Equity; Fairness; | ✔ | Continuous monitoring; | ✔ |
| **647** | Martiniussen, MA, et al. | 2023 |  |  | Beneficence; Harm; | ~ | Explainability; Transparency; Understandability; | ✔ | Responsibility; | ~ | Bias; | ✔ |  |  |
| **648** | Pot, M, et al. | 2021 |  |  | Beneficence; Harm; Safety; | ~ | Transparency; | ~ | Accountability; | ~ | Bias; Discrimination; Disparity; Equality; Equity; Fairness; Justice; | ✔ | Continuous monitoring; | ~ |
| **649** | Nichol, AA, et al. | 2023 | Privacy; | ~ | Efficacy; Harm; Safety; | ✔ |  |  | Accountability; Human oversight; Responsibility; | ✔ | Bias; | ~ |  |  |
| **651** | Zhang, C, et al. | 2023 | Autonomy; Privacy; | ✔ |  |  | Explainability; Interpretability; Transparency; Understandability; | ✔ | Accountability; Human supervision; | ~ | Bias; | ~ |  |  |
| **652** | Zicari, RV, et al. | 2021 | Autonomy; Human oversight; Informed consent; Privacy; | ✔ | Beneficence; Harm; Safety; Well-being; | ✔ | Explainability; Explicability; Interpretability; Transparency; Understandability; | ✔ | Accountability; Human supervision; Liability; Redress; Responsibility; | ✔ | Bias; Discrimination; Equality; Equity; Fairness; Inclusiveness; Justice; | ✔ | Continuous monitoring; Environment; Sustainability; | ~ |
| **653** | Herzog, C. | 2022 | Autonomy; Informed consent; Privacy; | ✔ | Beneficence; Efficacy; Harm; Non-maleficence; Well-being; | ✔ | Explainability; Explicability; Intelligibility; Interpretability; Transparency; Understandability; | ✔ | Accountability; Contestability; Human oversight; Responsibility; | ✔ | Bias; Discrimination; Fairness; | ~ |  |  |
| **654** | Grote, T, et al. | 2020 | Autonomy; Informed consent; Privacy; | ✔ | Beneficence; Efficacy; Harm; | ~ | Explainability; Intelligibility; Interpretability; Transparency; | ✔ | Accountability; Responsibility; | ✔ | Bias; Fairness; | ✔ |  |  |
| **655** | Holm, S | 2023 | Autonomy; Informed consent; | ~ | Safety; | ~ | Explainability; Interpretability; Understandability; | ✔ | Responsibility; | ✔ |  |  |  |  |
| **656** | Delshad, M, et al. | 2023 | Confidentiality; Informed consent; | ~ |  |  |  |  |  |  | Bias; | ~ |  |  |
| **657** | Solanki, P, et al. | 2023 | Autonomy; Confidentiality; Human oversight; Privacy; | ✔ | Beneficence; Harm; Non-maleficence; Safety; Well-being; | ✔ | Explainability; Interpretability; Transparency; Understandability; | ✔ | Accountability; Human supervision; Liability; Redress; Responsibility; | ✔ | Bias; Discrimination; Disparity; Equality; Equity; Fairness; Inclusiveness; Justice; | ✔ | Continuous monitoring; Ecological footprint; Environment; Sustainability; | ~ |
| **658** | Jiang, L, et al. | 2021 | Informed consent; Privacy; | ✔ | Harm; Safety; | ~ | Explainability; Interpretability; Transparency; Understandability; | ~ | Human supervision; Liability; Responsibility; | ✔ | Bias; Discrimination; Equality; Fairness; Justice; | ✔ |  |  |
| **660** | Cartus, AR, et al. | 2023 |  |  |  |  | Transparency; | ~ | Responsibility; | ~ | Fairness; | ~ |  |  |
| **661** | Sutton, RA, et al. | 2021 | Autonomy; Privacy; | ✔ | Harm; Safety; | ~ | Explainability; Interpretability; Transparency; Understandability; | ✔ | Responsibility; | ✔ | Equality; | ~ | Continuous monitoring; | ~ |
| **662** | Tan, L, et al. | 2020 | Informed consent; Privacy; | ~ | Beneficence; Harm; Safety; | ~ | Explainability; Transparency; | ✔ | Accountability; Contestability; Responsibility; | ~ | Bias; Discrimination; Equality; Fairness; | ~ |  |  |
| **663** | Baughan, N, et al. | 2022 | Privacy; | ~ | Beneficence; Efficacy; | ~ | Explainability; Interpretability; Understandability; | ✔ |  |  | Bias; Equity; | ~ |  |  |
| **664** | McCradden, MD, et al. | 2020 | Informed consent; | ~ | Efficacy; Harm; Non-maleficence; Safety; | ✔ | Explainability; Intelligibility; Interpretability; Transparency; | ~ | Accountability; Human oversight; | ✔ | Bias; Disparity; Equality; Fairness; Justice; | ✔ |  |  |
| **666** | Esmaeilzadeh, P, et al. | 2021 | Autonomy; Confidentiality; Privacy; | ✔ | Harm; Safety; Well-being; | ✔ | Transparency; Understandability; | ✔ | Accountability; Human supervision; Liability; Responsibility; | ✔ | Bias; Discrimination; Equity; Fairness; | ✔ | Continuous monitoring; | ~ |
| **668** | Ahun, E, et al. | 2023 | Autonomy; Confidentiality; Privacy; | ✔ | Beneficence; Safety; | ✔ | Transparency; | ~ | Accountability; Responsibility; | ✔ | Bias; Discrimination; Equity; Fairness; | ✔ |  |  |
| **669** | Li, B, et al. | 2022 | Privacy; | ✔ | Beneficence; Harm; Safety; | ✔ |  |  |  |  | Bias; | ✔ |  |  |
| **670** | Ray, TR, et al. | 2023 | Human oversight; Privacy; | ~ |  |  | Understandability; | ✔ |  |  | Bias; | ~ |  |  |
| **671** | Chen, Z, et al. | 2022 | Privacy; | ✔ |  |  |  |  |  |  |  |  |  |  |
| **672** | Al-Edresee, T | 2022 | Privacy; | ~ | Beneficence; | ~ | Explainability; Understandability; | ~ | Liability; Responsibility; | ~ |  |  |  |  |
| **673** | Pozzi, G, et al. | 2023 | Autonomy; | ~ |  |  |  |  | Responsibility; | ✔ | Bias; Equality; Fairness; Justice; | ✔ |  |  |
| **674** | Fehr, J, et al. | 2022 | Privacy; | ~ | Harm; Safety; Well-being; | ✔ | Interpretability; Transparency; | ✔ |  |  | Bias; Fairness; | ✔ |  |  |
| **675** | Bellini, V, et al. | 2021 | Privacy; | ~ |  |  |  |  |  |  | Bias; Fairness; | ~ |  |  |
| **676** | Bartlett, B | 2023 |  |  | Harm; Safety; | ~ | Explainability; Explicability; Interpretability; Transparency; Understandability; | ✔ | Accountability; Liability; Responsibility; | ✔ | Bias; Discrimination; | ✔ |  |  |
| **677** | Ray, PP, et al. | 2023 | Human oversight; Privacy; | ~ | Safety; | ~ | Understandability; | ~ | Accountability; Responsibility; | ~ | Bias; Equity; Fairness; | ~ | Continuous improvement; | ~ |
| **678** | Park, I, et al. | 2023 | Confidentiality; Human oversight; Privacy; | ~ | Safety; Well-being; | ~ |  |  | Accountability; Responsibility; | ~ | Bias; | ~ | Continuous monitoring; | ~ |
| **679** | Cheng, K, et al. | 2023 | Confidentiality; | ~ | Safety; | ~ |  |  | Liability; | ~ |  |  |  |  |
| **680** | Ma, H, et al. | 2019 | Confidentiality; Privacy; | ✔ |  |  |  |  |  |  |  |  |  |  |
| **681** | Zsidai, B, et al. | 2023 | Human oversight; | ~ | Harm; Safety; | ~ | Explainability; Interpretability; Transparency; | ✔ |  |  | Bias; Disparity; | ~ |  |  |
| **683** | Buchanan, C, et al. | 2020 | Privacy; | ~ | Harm; Safety; Well-being; | ~ |  |  | Responsibility; | ~ |  |  |  |  |
| **684** | Sharadin, N | 2023 | Autonomy; | ✔ | Harm; Safety; | ~ |  |  |  |  |  |  |  |  |
| **685** | Van Calster, B, et al. | 2019 |  |  |  |  | Transparency; Understandability; | ✔ |  |  |  |  | Continuous monitoring; | ~ |
| **686** | Sikstrom, L, et al. | 2023 |  |  | Harm; | ~ |  |  |  |  | Bias; Equity; Fairness; | ✔ |  |  |
| **687** | Alexander, JC, et al. | 2020 |  |  | Efficacy; Safety; | ~ | Explainability; Transparency; | ~ | Liability; | ~ | Bias; Equality; Fairness; | ✔ |  |  |
| **688** | Olczak, J, et al. | 2021 | Autonomy; Privacy; | ~ | Beneficence; Harm; Non-maleficence; Safety; | ~ | Explainability; Interpretability; Transparency; Understandability; | ✔ | Accountability; Liability; Responsibility; | ~ | Bias; Fairness; Justice; | ✔ |  |  |
| **689** | Moldoveanu, AC, et al. | 2022 | Autonomy; Human oversight; Privacy; | ~ |  |  | Explainability; Understandability; | ~ | Human supervision; Liability; | ~ |  |  |  |  |
| **690** | Braun, M, et al. | 2020 | Autonomy; Informed consent; Privacy; | ~ | Efficacy; Non-maleficence; Safety; | ~ | Explainability; Explicability; Transparency; Understandability; | ✔ | Accountability; Human supervision; Liability; Responsibility; | ✔ | Bias; Equity; Justice; | ~ |  |  |
| **692** | Murdoch, B | 2021 | Informed consent; Privacy; | ✔ |  |  | Interpretability; | ~ | Accountability; Human oversight; Liability; | ~ |  |  |  |  |
| **693** | Martinez-Martin, N, et al. | 2019 | Informed consent; | ~ |  |  | Explainability; Transparency; | ~ | Accountability; | ~ | Bias; Discrimination; | ✔ |  |  |
| **694** | Puiu, A, et al. | 2021 | Confidentiality; Privacy; | ✔ |  |  | Explainability; Interpretability; Transparency; | ✔ |  |  | Bias; | ~ |  |  |
| **695** | Khalid, N, et al. | 2023 | Confidentiality; Informed consent; Privacy; | ✔ |  |  | Explainability; Transparency; Understandability; | ~ |  |  | Bias; Fairness; | ~ |  |  |
| **696** | Thoret, E, et al. | 2021 |  |  |  |  | Explainability; Intelligibility; Interpretability; Understandability; | ✔ |  |  |  |  |  |  |
| **697** | Jiang, J, et al. | 2022 | Confidentiality; Privacy; | ~ |  |  | Interpretability; Understandability; | ~ |  |  | Bias; | ✔ |  |  |
| **698** | Mascarenhas, M, et al. | 2023 | Confidentiality; Informed consent; Privacy; | ✔ | Harm; Safety; | ~ | Explainability; Interpretability; Transparency; Understandability; | ✔ | Responsibility; | ✔ | Bias; Discrimination; Equality; | ✔ |  |  |
| **699** | Chekroud, AM, et al. | 2021 | Autonomy; Privacy; | ~ | Harm; Safety; | ~ | Explainability; Interpretability; Transparency; Understandability; | ✔ | Responsibility; | ~ | Bias; Discrimination; Fairness; | ✔ |  |  |
| **700** | Spector-Bagdady, K, et al. | 2022 | Autonomy; Informed consent; | ✔ |  |  |  |  | Human oversight; | ~ | Bias; Equity; Justice; | ✔ |  |  |
| **701** | Dankwa-Mullan, I, et al. | 2021 | Autonomy; Human oversight; Privacy; | ~ | Beneficence; Harm; Safety; Well-being; | ~ | Explainability; Transparency; Understandability; | ~ | Accountability; Responsibility; | ~ | Bias; Discrimination; Disparity; Equality; Equity; Fairness; Inclusiveness; Justice; | ✔ | Continuous monitoring; Environment; | ~ |
| **702** | Alonso, A, et al. | 2023 | Autonomy; Confidentiality; Informed consent; Privacy; | ✔ | Harm; Safety; | ~ | Explainability; Transparency; Understandability; | ~ | Accountability; Responsibility; | ~ | Bias; Disparity; Equity; | ~ |  |  |
| **703** | Balthazar, P, et al. | 2018 | Autonomy; Confidentiality; Informed consent; Privacy; | ✔ | Beneficence; Harm; Well-being; | ~ |  |  |  |  | Bias; Discrimination; Disparity; Justice; | ✔ |  |  |
| **705** | Mouchabac, S, et al. | 2020 | Autonomy; Confidentiality; Informed consent; Privacy; | ✔ | Beneficence; Harm; Non-maleficence; Safety; | ~ | Explainability; Transparency; Understandability; | ~ | Responsibility; | ~ | Discrimination; Equality; Equity; Justice; | ~ |  |  |
| **707** | Wu, C, et al. | 2023 | Autonomy; Confidentiality; Privacy; | ✔ | Efficacy; Harm; Public interest; Safety; | ✔ | Explainability; Transparency; Understandability; | ✔ | Accountability; Human supervision; Liability; Responsibility; | ✔ | Bias; Equality; Fairness; Justice; | ~ |  |  |
| **709** | Cahan, EM, et al. | 2019 | Informed consent; Privacy; | ~ | Efficacy; Harm; Well-being; | ~ | Transparency; | ~ |  |  | Bias; Discrimination; Disparity; Equity; Inclusiveness; | ✔ |  |  |
| **712** | McCrindle, B, et al. | 2021 |  |  |  |  | Explainability; Interpretability; Transparency; Understandability; | ✔ | Accountability; Human supervision; | ~ |  |  |  |  |
| **713** | Grote, T | 2022 | Autonomy; Human oversight; Informed consent; | ✔ | Beneficence; Harm; Non-maleficence; | ~ | Explicability; Interpretability; Transparency; Understandability; | ✔ | Accountability; | ~ | Bias; Discrimination; Disparity; Equality; Fairness; Justice; | ✔ | Sustainability; | ~ |
| **714** | Arbelaez Ossa, L, et al. | 2022 | Autonomy; | ~ | Harm; Safety; | ~ | Explainability; Intelligibility; Interpretability; Transparency; Understandability; | ✔ | Accountability; Contestability; Responsibility; | ~ | Bias; Discrimination; Fairness; | ~ |  |  |
| **715** | Wellnhofer, E | 2022 | Autonomy; Human oversight; Informed consent; Privacy; | ~ | Harm; Safety; Well-being; | ~ | Explainability; Explicability; Interpretability; Transparency; Understandability; | ✔ | Accountability; Human oversight; Liability; Responsibility; | ~ | Bias; Discrimination; Equality; Equity; Fairness; Inclusiveness; | ~ | Environment; | ~ |
| **716** | Kitsios, F, et al. | 2023 | Privacy; | ~ | Beneficence; Efficacy; Safety; Well-being; | ~ |  |  |  |  | Bias; Discrimination; Disparity; Equality; Fairness; Inclusiveness; | ~ | Sustainability; | ~ |
| **718** | Contino, G | 2023 | Autonomy; | ~ |  |  | Explainability; Interpretability; Transparency; | ~ |  |  | Bias; Equity; Fairness; | ~ |  |  |
| **719** | Najjar, R | 2023 | Autonomy; Confidentiality; Informed consent; Privacy; | ✔ | Harm; Safety; | ~ | Explainability; Explicability; Interpretability; Transparency; Understandability; | ✔ | Accountability; Human oversight; Liability; Responsibility; | ~ | Bias; Discrimination; Disparity; Equity; Justice; | ✔ | Continuous improvement; Continuous monitoring; | ~ |
| **720** | Chen, A, et al. | 2023 | Autonomy; Privacy; | ~ | Harm; Public interest; Safety; Well-being; | ~ | Interpretability; Transparency; Understandability; | ✔ | Accountability; Human supervision; Liability; Responsibility; | ✔ | Bias; Discrimination; Equity; Fairness; Inclusiveness; | ~ | Responsiveness; Sustainability; | ~ |
| **721** | Giansanti, D | 2022 | Autonomy; Confidentiality; Informed consent; Privacy; | ~ | Beneficence; Efficacy; Harm; Safety; | ~ | Transparency; | ~ | Human oversight; Liability; | ~ | Bias; Equity; Fairness; Justice; | ✔ |  |  |
| **722** | Harvey, HB, et al. | 2021 | Autonomy; Confidentiality; Human oversight; Informed consent; Privacy; | ✔ | Efficacy; Harm; Safety; | ~ |  |  | Human oversight; Liability; Responsibility; | ✔ | Bias; Discrimination; Disparity; | ~ |  |  |
| **723** | Minssen, T, et al. | 2020 | Privacy; | ~ | Safety; | ✔ | Transparency; Understandability; | ~ | Liability; | ~ | Bias; Discrimination; Equity; Fairness; Inclusiveness; | ✔ | Sustainability; | ~ |
| **724** | Iqbal, J, et al. | 2023 | Informed consent; Privacy; | ✔ | Efficacy; Harm; Safety; Well-being; | ~ | Transparency; Understandability; | ~ | Accountability; Liability; Responsibility; | ~ | Bias; Disparity; Fairness; Justice; | ~ |  |  |
| **725** | Kempt, H, et al. | 2022 | Informed consent; | ~ |  |  | Explainability; Explicability; Intelligibility; Interpretability; Transparency; Understandability; | ✔ | Responsibility; | ✔ |  |  |  |  |
| **726** | Carruthers, R, et al. | 2022 |  |  |  |  |  |  |  |  | Bias; Disparity; Equality; Equity; Fairness; | ✔ |  |  |
| **727** | Blumenthal-Barby, J, et al. | 2022 |  |  | Harm; | ~ |  |  | Human oversight; | ~ | Bias; Equity; | ~ |  |  |
| **728** | Funer, F, et al. | 2023 | Privacy; | ~ | Harm; Safety; | ~ | Explainability; Transparency; | ~ | Accountability; Liability; Responsibility; | ✔ |  |  |  |  |
| **729** | Sand, M, et al. | 2022 | Autonomy; Human oversight; Privacy; | ~ | Beneficence; Harm; Non-maleficence; Safety; | ✔ | Explainability; Interpretability; Transparency; Understandability; | ✔ | Accountability; Contestability; Liability; Responsibility; | ✔ | Bias; Discrimination; Fairness; | ~ | Continuous improvement; Continuous monitoring; | ✔ |
| **730** | Kempt, H, et al. | 2022 | Autonomy; | ~ | Harm; | ~ | Explainability; Intelligibility; Interpretability; | ✔ | Liability; Responsibility; | ✔ |  |  |  |  |
| **731** | Walsh, G, et al. | 2023 | Autonomy; Informed consent; Privacy; | ✔ | Beneficence; Harm; Non-maleficence; Safety; | ~ | Explainability; Transparency; Understandability; | ✔ | Accountability; Liability; Responsibility; | ~ | Bias; Discrimination; Equality; Equity; Fairness; Justice; | ✔ |  |  |
| **732** | Petersen, E, et al. | 2022 | Autonomy; Confidentiality; Human oversight; Privacy; | ✔ | Beneficence; Harm; Safety; | ✔ | Explainability; Explicability; Interpretability; Transparency; Understandability; | ✔ | Accountability; Contestability; Human oversight; Responsibility; | ~ | Bias; Discrimination; Disparity; Equality; Equity; Fairness; Inclusiveness; Justice; | ✔ | Climate; Continuous monitoring; | ✔ |
| **733** | Obasa, AE, et al. | 2023 | Autonomy; Human oversight; Informed consent; Privacy; | ✔ | Harm; Safety; Well-being; | ~ | Explainability; Explicability; Interpretability; Transparency; Understandability; | ✔ | Accountability; Human oversight; Liability; Responsibility; | ✔ | Bias; Equity; Inclusiveness; | ✔ | Sustainability; | ~ |
| **735** | Friedrich, AB, et al. | 2022 | Autonomy; | ~ | Beneficence; Efficacy; Harm; Safety; | ~ | Explainability; Understandability; | ✔ | Accountability; Liability; Responsibility; | ~ | Bias; Discrimination; | ✔ |  |  |
| **736** | Denecke, K, et al. | 2022 | Autonomy; Privacy; | ~ | Beneficence; Efficacy; Harm; Safety; | ✔ | Explainability; Intelligibility; Transparency; | ~ | Accountability; Liability; Responsibility; | ~ | Bias; Discrimination; Fairness; | ✔ |  |  |
| **737** | Al Kuwaiti, A, et al. | 2023 | Autonomy; Confidentiality; Informed consent; Privacy; | ~ | Beneficence; Efficacy; Harm; Non-maleficence; Safety; Well-being; | ~ | Explainability; Interpretability; Transparency; Understandability; | ✔ | Accountability; Liability; | ~ | Bias; Equity; Fairness; | ~ |  |  |
| **738** | Birkhoff, DC, et al. | 2021 | Privacy; | ~ | Safety; | ~ |  |  | Human supervision; | ~ |  |  |  |  |
| **739** | Kusunose, K | 2023 | Informed consent; Privacy; | ~ | Efficacy; Harm; | ~ |  |  | Liability; | ~ | Bias; | ~ |  |  |
| **740** | Xiao, D, et al. | 2023 | Human oversight; Privacy; | ~ | Beneficence; Harm; Safety; | ~ | Explainability; Understandability; | ~ | Responsibility; | ~ |  |  | Continuous monitoring; | ~ |
| **741** | Alowais, SA, et al. | 2023 | Autonomy; Confidentiality; Human oversight; Informed consent; Privacy; | ✔ | Efficacy; Safety; Well-being; | ~ | Transparency; | ~ | Accountability; Human supervision; Responsibility; | ~ | Bias; Equity; Fairness; | ~ |  |  |
| **742** | Yagi, M, et al. | 2023 | Informed consent; Privacy; | ~ | Beneficence; Safety; | ~ | Explainability; Interpretability; Transparency; Understandability; | ~ | Accountability; Human oversight; Responsibility; | ~ | Bias; Equity; Fairness; | ~ | Continuous monitoring; | ~ |
| **743** | Pierce, R, et al. | 2022 | Informed consent; | ~ | Beneficence; Efficacy; Harm; Non-maleficence; | ~ | Explainability; Interpretability; Transparency; | ✔ | Responsibility; | ~ | Bias; Disparity; Equity; | ✔ |  |  |
| **744** | Sveen, W, et al. | 2022 |  |  | Harm; | ~ |  |  |  |  | Bias; Discrimination; Disparity; Equality; Equity; Fairness; Justice; | ✔ |  |  |
| **745** | Willem, T, et al. | 2022 | Autonomy; Confidentiality; Informed consent; Privacy; | ✔ | Beneficence; Efficacy; Harm; Non-maleficence; Safety; | ✔ | Explainability; Interpretability; Transparency; Understandability; | ~ | Accountability; Liability; Responsibility; | ~ | Bias; Discrimination; Disparity; Equity; Fairness; Inclusiveness; Justice; | ✔ | Environment; | ~ |
| **746** | Valles-Peris, N, et al. | 2021 | Autonomy; | ~ | Efficacy; Well-being; | ✔ |  |  |  |  |  |  |  |  |
| **747** | Brereton, TA, et al. | 2023 | Autonomy; Informed consent; Privacy; | ~ | Efficacy; Harm; Safety; | ~ | Explainability; Interpretability; Transparency; Understandability; | ✔ | Accountability; Contestability; Human oversight; Liability; Responsibility; | ✔ | Bias; Disparity; Equality; Equity; Fairness; | ✔ | Continuous monitoring; | ~ |
| **748** | Jiang, VS, et al. | 2023 | Autonomy; Privacy; | ~ | Harm; Safety; | ✔ | Transparency; | ~ | Accountability; | ~ | Bias; Discrimination; Disparity; Equality; Fairness; | ✔ | Continuous monitoring; | ~ |
| **749** | Hunter, B, et al. | 2022 |  |  | Efficacy; Safety; | ~ | Explainability; | ~ |  |  | Bias; Fairness; | ✔ |  |  |
| **750** | Raturi, M, et al. | 2023 | Confidentiality; | ~ |  |  |  |  |  |  | Bias; | ~ |  |  |
| **751** | Mohsin, SN, et al. | 2023 | Confidentiality; Human oversight; Informed consent; Privacy; | ~ | Efficacy; Safety; | ~ | Explainability; Interpretability; Transparency; Understandability; | ~ |  |  | Bias; Fairness; | ~ | Continuous monitoring; | ~ |
| **752** | Landry, G, et al. | 2023 | Informed consent; Privacy; | ✔ | Harm; | ~ |  |  | Responsibility; | ~ | Bias; Discrimination; Equality; | ~ |  |  |
| **754** | Chlorogiannis, DD, et al. | 2023 | Human oversight; Privacy; | ~ | Harm; | ~ |  |  | Responsibility; | ~ | Bias; Discrimination; Equity; | ✔ |  |  |
| **755** | D'Amore, B, et al. | 2021 |  |  |  |  | Transparency; | ~ | Accountability; | ~ |  |  |  |  |
| **756** | Christou, CD, et al. | 2022 |  |  | Safety; Well-being; | ~ | Interpretability; Transparency; | ~ | Liability; Responsibility; | ✔ | Bias; Equality; | ✔ | Continuous monitoring; | ~ |
| **757** | Evans, BJ | 2023 | Autonomy; Confidentiality; Human oversight; Informed consent; Privacy; | ✔ | Beneficence; Harm; Non-maleficence; Safety; Well-being; | ✔ | Transparency; | ~ | Liability; Responsibility; | ~ | Bias; Discrimination; Disparity; Equality; Equity; Fairness; Inclusiveness; Justice; | ✔ |  |  |
| **758** | Hallows, R, et al. | 2022 | Autonomy; | ~ | Harm; Safety; Well-being; | ✔ | Transparency; | ~ |  |  | Bias; | ~ | Continuous monitoring; | ~ |
| **759** | Canales, C, et al. | 2020 | Autonomy; Human oversight; Informed consent; Privacy; | ✔ | Safety; | ~ | Explainability; Transparency; Understandability; | ✔ | Human oversight; Liability; Responsibility; | ✔ | Bias; Discrimination; Disparity; Equality; Equity; Inclusiveness; | ✔ | Continuous monitoring; | ~ |
| **760** | Bear Don't Walk, OJt, et al. | 2022 | Autonomy; Informed consent; Privacy; | ✔ | Beneficence; Harm; Non-maleficence; Well-being; | ✔ | Explicability; Intelligibility; Interpretability; Understandability; | ✔ | Accountability; Human oversight; Responsibility; | ~ | Bias; Discrimination; Disparity; Equality; Equity; Fairness; Justice; | ✔ |  |  |
| **763** | Kaissis, GA, et al. | 2020 | Confidentiality; Informed consent; Privacy; | ✔ |  |  | Interpretability; Transparency; | ~ |  |  | Bias; Fairness; | ~ | Continuous monitoring; | ~ |
| **764** | Begley, K, et al. | 2021 | Autonomy; | ~ | Beneficence; | ~ | Explainability; Interpretability; Transparency; Understandability; | ✔ |  |  | Bias; | ~ |  |  |
| **766** | Mirkin, S, et al. | 2023 | Informed consent; Privacy; | ✔ | Beneficence; Harm; Safety; | ~ | Transparency; | ~ | Accountability; Responsibility; | ~ |  |  |  |  |
| **767** | Benzinger, L, et al. | 2023 | Autonomy; | ✔ | Beneficence; Non-maleficence; | ~ | Explainability; Explicability; Transparency; Understandability; | ✔ | Accountability; | ~ | Bias; Equity; Fairness; Justice; | ✔ |  |  |
| **768** | Cortese, J, et al. | 2023 | Autonomy; Informed consent; Privacy; | ✔ | Beneficence; Harm; Non-maleficence; Safety; Well-being; | ✔ | Explainability; Explicability; Intelligibility; Interpretability; Transparency; Understandability; | ✔ | Accountability; Responsibility; | ~ | Bias; Fairness; Justice; | ✔ | Sustainability; | ~ |
| **769** | Luxton, DD | 2019 |  |  | Harm; Safety; | ~ | Explainability; Understandability; | ~ | Human oversight; Human supervision; Liability; Responsibility; | ✔ |  |  |  |  |
| **770** | Binkley, CE, et al. | 2022 |  |  |  |  |  |  |  |  | Bias; Disparity; Equality; Equity; | ✔ |  |  |
| **771** | Ahmad, MA, et al. | 2023 | Autonomy; | ~ | Beneficence; Non-maleficence; | ~ | Explainability; Explicability; Interpretability; Transparency; Understandability; | ✔ | Accountability; | ~ | Bias; Discrimination; Equality; Equity; Fairness; Justice; | ✔ | Continuous monitoring; | ~ |
| **772** | Currie, G, et al. | 2022 | Autonomy; Privacy; | ~ | Beneficence; Harm; Non-maleficence; | ~ | Explainability; Transparency; | ~ | Liability; | ~ | Bias; Discrimination; Disparity; Equality; Equity; Fairness; Inclusiveness; Justice; | ✔ |  |  |
| **773** | Garcha, I, et al. | 2023 | Privacy; | ~ |  |  |  |  | Human oversight; | ~ | Bias; Disparity; Equity; Inclusiveness; | ✔ |  |  |
| **774** | Vellido, A | 2019 | Autonomy; Confidentiality; Privacy; | ✔ | Beneficence; Non-maleficence; Safety; | ~ | Explainability; Interpretability; Transparency; Understandability; | ✔ | Responsibility; | ~ | Bias; Discrimination; Fairness; Justice; | ✔ |  |  |
| **776** | Lupton, M. | 2018 | Informed consent; | ~ | Harm; Safety; | ~ |  |  | Accountability; Liability; Redress; Responsibility; | ✔ |  |  |  |  |
| **777** | Villongco, C, et al. | 2020 | Informed consent; Privacy; | ~ | Beneficence; Harm; Safety; | ~ |  |  | Accountability; Human oversight; Responsibility; | ~ | Bias; Discrimination; Disparity; Equality; Fairness; Justice; | ✔ |  |  |
| **778** | Sujan, MA, et al. | 2022 | Autonomy; Human oversight; | ✔ | Harm; Safety; Well-being; | ✔ | Explainability; Understandability; | ~ | Accountability; Responsibility; | ✔ | Bias; Equity; Fairness; | ~ | Continuous monitoring; | ~ |
| **779** | Yang, L, et al. | 2022 | Confidentiality; Human oversight; Informed consent; Privacy; | ~ |  |  |  |  | Accountability; Responsibility; | ✔ |  |  |  |  |
| **782** | Witte, H, et al. | 2023 | Confidentiality; Informed consent; Privacy; | ✔ |  |  | Explainability; Transparency; | ~ |  |  | Bias; | ~ |  |  |
| **783** | Walsh, CG, et al. | 2020 | Privacy; | ~ | Efficacy; Harm; Safety; | ~ | Explainability; Interpretability; Transparency; Understandability; | ✔ | Accountability; Liability; Responsibility; | ~ | Bias; Discrimination; Disparity; Fairness; | ✔ |  |  |
| **784** | Ahluwalia, M, et al. | 2023 |  |  | Safety; | ~ |  |  |  |  | Bias; Disparity; Equity; Fairness; | ✔ | Continuous monitoring; | ~ |
| **786** | du Toit, C, et al. | 2023 | Informed consent; Privacy; | ~ | Harm; | ~ | Explainability; Interpretability; Transparency; Understandability; | ✔ |  |  | Bias; Discrimination; Equality; Equity; Fairness; | ✔ |  |  |
| **788** | Ahmed, MI, et al. | 2023 | Autonomy; Confidentiality; Human oversight; Informed consent; Privacy; | ✔ | Harm; Safety; Well-being; | ✔ | Explainability; Interpretability; Transparency; Understandability; | ✔ | Accountability; Liability; Responsibility; | ✔ | Bias; Discrimination; Equality; Equity; Fairness; Justice; | ✔ |  |  |
| **789** | Albahri, AS, et al. | 2023 | Autonomy; Confidentiality; Human oversight; Informed consent; Privacy; | ✔ | Harm; Safety; Well-being; | ✔ | Explainability; Explicability; Intelligibility; Interpretability; Transparency; Understandability; | ✔ | Accountability; Redress; Responsibility; | ~ | Bias; Discrimination; Equality; Equity; Fairness; Justice; | ✔ | Continuous monitoring; Energy efficiency; Environment; Sustainability; | ~ |
| **790** | Panjiyar, BK, et al. | 2023 |  |  | Safety; | ~ | Transparency; | ~ | Human oversight; | ~ | Bias; Fairness; | ~ | Continuous monitoring; | ~ |
| **791** | Vayena, E, et al. | 2022 |  |  |  |  | Interpretability; | ~ | Human oversight; | ✔ |  |  |  |  |
| **793** | Arnold, MH, et al. | 2021 | Autonomy; Confidentiality; Informed consent; Privacy; | ✔ | Beneficence; Efficacy; Harm; Non-maleficence; Safety; Well-being; | ✔ | Explainability; Explicability; Intelligibility; Transparency; | ~ |  |  | Bias; Discrimination; Disparity; Equality; Equity; Fairness; Justice; | ✔ |  |  |
| **794** | Hu, Z, et al. | 2022 | Informed consent; Privacy; | ✔ | Beneficence; Harm; Non-maleficence; Safety; | ✔ | Explainability; Interpretability; Transparency; | ~ | Human supervision; Liability; Responsibility; | ~ | Bias; Disparity; Equity; | ✔ |  |  |
| **795** | Müller, H, et al. | 2021 | Informed consent; | ~ | Harm; Well-being; | ~ | Explainability; Transparency; Understandability; | ✔ | Accountability; Responsibility; | ~ | Bias; Discrimination; Equity; Fairness; Justice; | ✔ |  |  |
| **796** | Pozzi, G | 2023 | Autonomy; | ~ | Beneficence; Harm; | ~ |  |  | Contestability; Responsibility; | ~ | Bias; Discrimination; Equality; Fairness; Justice; | ✔ |  |  |
| **797** | Rivard, L, et al. | 2023 | Privacy; | ~ | Efficacy; Safety; | ~ | Explainability; Transparency; | ~ | Accountability; Responsibility; | ✔ | Bias; Discrimination; Equality; Equity; Fairness; Inclusiveness; | ~ | Carbon; Climate; Continuous monitoring; Environment; Responsiveness; Sustainability; | ✔ |
| **798** | Quinn, TP, et al. | 2022 | Autonomy; Informed consent; | ✔ | Beneficence; Efficacy; Harm; Safety; | ✔ | Explainability; Intelligibility; Interpretability; Transparency; Understandability; | ✔ | Accountability; Liability; | ~ | Bias; Discrimination; Fairness; | ✔ |  |  |
| **799** | Starke, G, et al. | 2021 |  |  | Harm; Safety; Well-being; | ~ | Explainability; Interpretability; Transparency; Understandability; | ~ |  |  | Bias; Discrimination; Disparity; Fairness; Justice; | ✔ |  |  |
| **800** | Padhi, A, et al. | 2023 | Autonomy; Human oversight; Privacy; | ✔ | Efficacy; Safety; | ~ | Explainability; Interpretability; Transparency; Understandability; | ~ | Accountability; Human oversight; Responsibility; | ~ | Bias; Disparity; Equality; Equity; Fairness; | ✔ | Continuous monitoring; | ✔ |
| **801** | Economou-Zavlanos, NJ, et al. | 2023 | Human oversight; | ~ | Harm; Safety; | ~ | Interpretability; Transparency; | ~ | Accountability; Responsibility; | ~ | Bias; Disparity; Equity; Fairness; | ~ | Continuous monitoring; | ✔ |
| **802** | Liu, M, et al. | 2023 |  |  | Well-being; | ~ | Explainability; Interpretability; Understandability; | ~ |  |  | Bias; Disparity; Equality; Equity; Fairness; Justice; | ✔ | Continuous monitoring; | ~ |
| **803** | Kostick-Quenet, K, et al. | 2023 |  |  | Safety; | ~ | Explainability; Interpretability; Transparency; Understandability; | ✔ | Responsibility; | ~ | Bias; | ~ |  |  |
| **804** | Nickel, PJ | 2022 |  |  | Well-being; | ~ | Explainability; Transparency; Understandability; | ✔ | Accountability; Responsibility; | ✔ | Bias; Fairness; | ~ | Continuous monitoring; | ~ |
| **805** | Crigger, E, et al. | 2022 | Autonomy; Human oversight; Informed consent; Privacy; | ~ | Efficacy; Harm; Safety; Well-being; | ~ | Explainability; Explicability; Intelligibility; Transparency; Understandability; | ~ | Accountability; Human oversight; Liability; Responsibility; | ✔ | Bias; Discrimination; Disparity; Equity; Fairness; Inclusiveness; | ✔ | Continuous improvement; Continuous monitoring; | ✔ |
| **806** | Tripathi, S, et al. | 2022 |  |  | Safety; | ~ |  |  |  |  | Bias; Fairness; | ✔ |  |  |
| **807** | Rainey, C, et al. | 2022 |  |  | Safety; | ~ | Explainability; Intelligibility; Interpretability; Transparency; Understandability; | ✔ | Responsibility; | ~ | Bias; | ~ |  |  |
| **808** | Seyyed-Kalantari, L, et al. | 2021 |  |  | Harm; | ~ |  |  |  |  | Bias; Disparity; Equality; Equity; Fairness; | ✔ |  |  |
| **809** | Ciet, P, et al. | 2023 | Human oversight; Privacy; | ~ | Beneficence; Efficacy; Harm; Safety; Well-being; | ~ | Explainability; Explicability; Interpretability; Transparency; | ~ | Accountability; Liability; Responsibility; | ✔ | Bias; Equality; Equity; Fairness; Inclusiveness; | ✔ | Continuous monitoring; Environment; | ~ |
| **810** | Alhajahjeh, A, et al. | 2023 | Privacy; | ~ |  |  | Explainability; Interpretability; Transparency; Understandability; | ~ | Responsibility; | ~ | Bias; | ~ |  |  |
| **811** | Waters, MR, et al. | 2023 | Human oversight; Privacy; | ✔ | Safety; | ~ | Understandability; | ~ |  |  |  |  |  |  |
| **812** | Jeyaraman, M, et al. | 2023 | Autonomy; Confidentiality; Privacy; | ✔ | Safety; Well-being; | ~ | Explainability; Interpretability; Transparency; Understandability; | ✔ | Accountability; Liability; Responsibility; | ✔ | Bias; Disparity; Equality; Equity; Fairness; | ✔ |  |  |
| **814** | Rao, D | 2023 | Privacy; | ~ |  |  |  |  | Responsibility; | ~ |  |  |  |  |
| **815** | Pecqueux, M, et al. | 2022 |  |  | Safety; | ~ |  |  | Accountability; Liability; Responsibility; | ~ |  |  |  |  |
| **817** | Farmer, N, et al. | 2022 |  |  |  |  |  |  |  |  | Bias; Discrimination; Equity; | ✔ |  |  |
| **818** | Esmaeilzadeh, P | 2020 | Autonomy; Confidentiality; Privacy; | ✔ | Beneficence; Efficacy; Harm; Safety; Well-being; | ~ | Interpretability; Transparency; Understandability; | ✔ | Accountability; Human supervision; Liability; Responsibility; | ✔ | Bias; Discrimination; Equity; Fairness; | ✔ | Continuous improvement; Continuous monitoring; | ~ |
| **819** | Macri, R, et al. | 2023 | Autonomy; Confidentiality; Human oversight; Privacy; | ✔ | Beneficence; Efficacy; Harm; Non-maleficence; Safety; | ✔ | Explainability; Interpretability; Transparency; Understandability; | ✔ | Accountability; Contestability; Human supervision; Liability; Responsibility; | ~ | Bias; Discrimination; Equity; Fairness; Inclusiveness; Justice; | ✔ | Environment; | ~ |
| **820** | Seth, I, et al. | 2023 | Privacy; | ~ | Efficacy; Safety; | ~ |  |  |  |  |  |  |  |  |
| **821** | Stokes, K, et al. | 2022 | Informed consent; | ~ | Efficacy; | ~ | Interpretability; Transparency; | ~ |  |  | Bias; | ~ |  |  |
| **823** | Raman, R, et al. | 2021 | Privacy; | ~ | Efficacy; Harm; Safety; | ~ | Explainability; Interpretability; Transparency; Understandability; | ✔ |  |  | Bias; Discrimination; Equality; Equity; | ✔ | Continuous monitoring; | ~ |
| **824** | Abedi, V, et al. | 2020 | Autonomy; | ~ | Beneficence; Efficacy; Safety; | ~ |  |  |  |  | Bias; Fairness; | ~ |  |  |
| **826** | Ashton, JJ, et al. | 2023 | Human oversight; Informed consent; | ✔ |  |  | Interpretability; Transparency; | ~ |  |  |  |  |  |  |
| **829** | Fasterholdt, I, et al. | 2022 | Informed consent; Privacy; | ~ | Safety; | ~ | Explainability; Interpretability; Transparency; | ~ | Responsibility; | ~ | Bias; Discrimination; | ~ |  |  |
| **830** | Arora, A, et al. | 2023 | Privacy; | ~ | Harm; Safety; | ~ | Transparency; Understandability; | ✔ | Accountability; | ~ | Bias; Discrimination; Disparity; Equality; Equity; Fairness; Inclusiveness; | ✔ |  |  |
| **831** | Jayakumar, P, et al. | 2023 | Autonomy; Human oversight; Privacy; | ~ | Harm; Safety; Well-being; | ~ | Transparency; | ~ | Accountability; Liability; Responsibility; | ~ | Bias; Disparity; Equity; | ✔ | Continuous monitoring; | ~ |
| **832** | Hatherley, J, et al. | 2023 |  |  | Harm; Safety; Well-being; | ~ | Explainability; Interpretability; Transparency; Understandability; | ✔ | Accountability; Liability; Responsibility; | ~ | Bias; | ~ |  |  |
| **834** | The Lancet Digital Health | 2019 | Autonomy; Human oversight; Privacy; | ~ | Harm; Safety; Well-being; | ~ | Explainability; Transparency; | ~ | Accountability; | ~ | Bias; Discrimination; Fairness; | ~ | Environment; | ~ |
| **835** | Petkus, H, et al. | 2020 |  |  | Harm; Safety; | ~ | Explainability; Interpretability; Transparency; Understandability; | ~ | Liability; | ~ | Bias; Fairness; | ~ |  |  |
| **836** | Du-Harpur, X, et al. | 2020 | Autonomy; | ~ | Harm; Safety; | ~ | Explainability; | ~ | Accountability; Liability; Responsibility; | ~ | Bias; | ✔ |  |  |
| **837** | de Boer, B, et al. | 2021 | Autonomy; Confidentiality; Privacy; | ~ | Harm; Safety; | ~ | Explainability; Intelligibility; Interpretability; Transparency; Understandability; | ✔ | Accountability; Human supervision; Liability; Responsibility; | ✔ | Bias; Equality; Inclusiveness; | ✔ |  |  |
| **839** | McCradden, MD, et al. | 2023 | Autonomy; Human oversight; | ✔ | Harm; Safety; Well-being; | ~ | Explainability; Interpretability; Transparency; Understandability; | ~ | Accountability; Liability; Responsibility; | ✔ | Bias; Discrimination; Disparity; Equality; Equity; Fairness; Inclusiveness; Justice; | ✔ | Continuous monitoring; Environment; | ~ |
| **840** | Banja, JD, et al. | 2022 | Informed consent; | ~ | Harm; Safety; | ~ | Explainability; Interpretability; Transparency; | ✔ | Liability; Responsibility; | ✔ | Bias; Fairness; | ~ |  |  |
| **842** | Duran, JM, et al. | 2021 | Autonomy; | ✔ | Safety; | ~ | Explainability; Interpretability; Transparency; Understandability; | ✔ | Accountability; Responsibility; | ✔ | Bias; Discrimination; | ~ |  |  |
| **844** | Reading Turchioe, M, et al. | 2023 | Confidentiality; Privacy; | ~ | Harm; Safety; Well-being; | ✔ | Explainability; Interpretability; Transparency; | ~ | Responsibility; | ~ |  |  |  |  |
| **845** | Fiske, A, et al. | 2019 | Autonomy; Confidentiality; Privacy; | ✔ | Beneficence; Harm; Non-maleficence; | ✔ | Explainability; Transparency; Understandability; | ~ | Human supervision; Responsibility; | ✔ | Bias; Equality; Justice; | ✔ | Continuous monitoring; Responsiveness; | ~ |

eFigure 1. Geographic distribution of included studies

*The colour intensity on this world map represents the number of included studies. Dark blue corresponds to the highest number of included studies, light blue indicates an intermediate number, and grey indicates areas without any included studies. Most of the included studies originated from North America (United States of America & Canada), whereas the fewest were from Africa.*

eFigure 2. Adoption of the six WHO ethical principles across generative AI publications (N=60)
*Each bar shows the proportion of included papers that consider the principle in depth (light blue), mention it briefly (medium blue), or do not adopt the principle (dark blue). WHO1=protecting human autonomy; WHO2=promoting well-being and safety; WHO3=ensuring transparency and explainability; WHO4=fostering responsibility and accountability; WHO5=ensuring inclusiveness and equity; WHO6=promoting responsive and sustainable AI.*

AI = Artificial Intelligence; WHO = World Health Organization.

# eFigure 3. Adoption of the six WHO ethical principles across studies developing clinical AI applications (N=44)

*Each bar shows the proportion of included papers that consider the principle in depth (light blue), mention it briefly (medium blue), or do not adopt the principle (dark blue). WHO1=protecting human autonomy; WHO2=promoting well-being and safety; WHO3=ensuring transparency and explainability; WHO4=fostering responsibility and accountability; WHO5=ensuring inclusiveness and equity; WHO6=promoting responsive and sustainable AI.*

AI = Artificial Intelligence; WHO = World Health Organization.

# References

1.	Ethics and governance of artificial intelligence for health (2021).

2.	Fleuren LM, Thoral P, Shillan D, Ercole A, Elbers PWG. Machine learning in intensive care medicine: ready for take-off? *Intensive Care Med*. Jul 2020;46(7):1486-1488. doi:10.1007/s00134-020-06045-y
